# Influence of Cooling duration on Efficacy in Cardiac Arrest Patients (ICECAP): study protocol for a multicenter, randomized, adaptive allocation clinical trial to identify the optimal duration of induced hypothermia for neuroprotection in comatose, adult survivors of after out-of-hospital cardiac arrest

**DOI:** 10.1186/s13063-024-08280-w

**Published:** 2024-07-23

**Authors:** William J. Meurer, Florian F. Schmitzberger, Sharon Yeatts, Viswanathan Ramakrishnan, Benjamin Abella, Tom Aufderheide, William Barsan, Justin Benoit, Scott Berry, Joy Black, Nia Bozeman, Kristine Broglio, Jeremy Brown, Kimberly Brown, Noelle Carlozzi, Angela Caveney, Sung-Min Cho, Hangyul Chung-Esaki, Robert Clevenger, Robin Conwit, Richelle Cooper, Valentina Crudo, Mohamud Daya, Deneil Harney, Cindy Hsu, Nicholas J. Johnson, Imad Khan, Shaveta Khosla, Peyton Kline, Anna Kratz, Peter Kudenchuk, Roger J. Lewis, Chaitra Madiyal, Sara Meyer, Jarrod Mosier, Marwan Mouammar, Matthew Neth, Brian O’Neil, James Paxton, Sofia Perez, Sarah Perman, Cemal Sozener, Mickie Speers, Aimee Spiteri, Valerie Stevenson, Kavita Sunthankar, Joseph Tonna, Scott Youngquist, Romergryko Geocadin, Robert Silbergleit, ICECAP trial investigators, Sarah Abdallah, David Adler, Sachin Agarwal, Rachel Ambroch, Amer Ardati, Farhan Ayaz, Maranatha Ayodele, Meenakshi Balakrishnan, Aaron Barksdale, Torben K. Becker, David Beiser, Margaret Beyer, Ethan Brandler, Lauren Buck, Margaret Carlson, Jeffrey Caterino, Dong Chang, Wan-Tsu Chang, M. Riccardo Colella, Alexandrea Cronin, Anna Curtis, Marina Del Rios, Alan Denney, Kenneth W. Dodd, Brian Driver, Timothy Ellender, Jonathan Elmer, Dominique Evans, Octavio Falcucci, Jennifer Fowler, Madison Frazier, Megan Fuentes, David Gaieski, Nina Gentile, Emily Gilmore, Adit Ginde, Joshua Goldstein, Gillian Grafton, Matthew Greer, Valerie Griffeth, Vedant Gupta, Alex Hall, Grace Hall, Christopher Harden, Robert Harrison, Jason Haukoos, J. Claude Hemphill, Terra Hiller, Karen Hirsch, Benton Hunter, Cameron Hypes, Ahamed Idris, Nastasia James, Namita Jayaprakash, Elizabeth Jones, Christopher Jones, Gregory Kapinos, Sarah Katsandres, Adam Kessler, Joy Kim, Kathryn Kisken, Howard Klausner, Jennifer Klavens, William Knight, Michael Christopher Kurz, Jacob Labinski, Nancy Le, Christopher Lewandowski, Alexander Limkakeng, Carol Lynn Lyle, David Marks, Ellen Maruggi, Linda Mattrisch, Teresa May, Thomas Mazzocco, Brenna McKaig, Eric Melnychuk, Lisa H. Merck, Valerie Mika, Joseph Miller, Benjamin Miller, James Miner, Wazim Mohamed, Karli Molignoni, Cindy Montero, Kayla Muschong, Sung Min Na, Rahul Nanchal, Robert Neumar, Daniel Nishijima, Katherine Nugent, Robert E. O’Connor, Gloria Obialisi, Joseph Ornato, Alex Parker, Blair Alden Parry, Nivedita Patkar, Jonathan Paul, MaryAnn Peberdy, Lars-Kristofer Peterson, Tedra Porter, Matthew Prekker, Peter Pruitt, Yanina Purim-Shem-Tov, Michael Puskarich, Dominica Randazzo, Lauren Remboski, Joshua Reynolds, Jon Rittenberger, Christopher Roberts, Robert Rodriguez, Birendra Sah, Christian Sebat, Rebecca Sell, Reza Shaker, Shelby Shelton, Arun Sherma, Michael Silverman, Bonnie Siry, Bill Spurlock, Vasisht Srinivasan, Nicholas Stanley, Sidney Starkman, Edward Suh, Timothy Thayer, Michael Thomas, Kathryn Thompson, Juliana Tolles, Shahed Toossi, Sam Torbati, Abigail Vollmer, Henry Wang, Gabriel Wardi, Nicholas Wesner, Kathleen Wilson, Charles Wira, Nancy Wood, Kabir Yadav, Jenny Yang, Lara Zimmermann, Michael D. Zwank

**Affiliations:** 1 Emergency Medicine, Neurology, University of Michigan, TC B1-354, 1500 E. Medical Center Drive, Ann Arbor, MI 48109-5301 USA; 2 Emergency Medicine, University of Michigan, Ann Arbor, MI USA; 3 Public Health Sciences, Medical University of South Carolina, Charleston, SC USA; 4 Emergency Medicine, University of Pennsylvania, Philadelphia, PA USA; 5 Emergency Medicine, Medical College of Wisconsin, Milwaukee, WI USA; 6 Emergency Medicine, University of Cincinnati, Cincinnati, OH USA; 7 Berry Consultants, Austin, TX USA; 8 Emergency Medicine, Neuroscience, University of Michigan, Thermo Fisher Scientific, Ann Arbor, MI USA; 9 Berry Consultants, Oncology Statistical Innovation, Gaithersburg, MD USA; 10 National Institutes of Health, National Institute of Neurological Disorders and Stroke, Bethesda, MD USA; 11 Physical Medicine and Rehabilitation, Surgery, University of Michigan, Ann Arbor, MI USA; 12 Critical Care Medicine, Johns Hopkins Hospital, Anesthesia, Baltimore, MD USA; 13 The Queen’s Medical Center, University of Hawaii John A. Burns School of Medicine, Critical Care, Honolulu, HI USA; 14 Neurology, Indiana University, Indianapolis, IN USA; 15 Emergency Medicine, UCLA David Geffen School of Medicine, Los Angeles, CA USA; 16 Emergency Medicine, Oregon Health & Science University, Portland, OR USA; 17 Emergency Medicine, Division of Pulmonary, Critical Care, and Sleep Medicine, Medicine, University of Washington, Seattle, WA USA; 18 Neurology, University of Rochester Medical Center, Rochester, NY USA; 19 Emergency Medicine, University of Illinois Chicago, Chicago, IL USA; 20 Division of Cardiology, Medicine, University of Washington, Seattle, WA USA; 21 Emergency Medicine, Medicine, University of Arizona, Tucson, AZ USA; 22 Medicine, Critical Care Medicine, OHSU Portland Adventist Medical Center, Portland, OR USA; 23 Emergency Medicine, Wayne State University, Detroit, MI USA; 24 Emergency Medicine Research, University of Michigan, Ann Arbor, MI USA; 25 Emergency Medicine, Yale School of Medicine, New Haven, CT USA; 26 Surgery, University of Utah Health, Salt Lake City, UT USA; 27 Emergency Medicine, Spencer Eccles School of Medicine at the University of Utah, Salt Lake City, UT USA; 28 Neurology, Anesthesiology-Critical Care Medicine, Neurosurgery, Johns Hopkins University School of Medicine, Baltimore, MD USA

**Keywords:** Hypothermia, Induced, Cardiopulmonary Resuscitation, Neuroprotection; Out-of-Hospital Cardiac Arrest,, Bayesian adaptive trial

## Abstract

**Background:**

Cardiac arrest is a common and devastating emergency of both the heart and brain. More than 380,000 patients suffer out-of-hospital cardiac arrest annually in the USA. Induced cooling of comatose patients markedly improved neurological and functional outcomes in pivotal randomized clinical trials, but the optimal duration of therapeutic hypothermia has not yet been established.

**Methods:**

This study is a multi-center randomized, response-adaptive, duration (dose) finding, comparative effectiveness clinical trial with blinded outcome assessment. We investigate two populations of adult comatose survivors of cardiac arrest to ascertain the shortest duration of cooling that provides the maximum treatment effect. The design is based on a statistical model of response as defined by the primary endpoint, a weighted 90-day mRS (modified Rankin Scale, a measure of neurologic disability), across the treatment arms. Subjects will initially be equally randomized between 12, 24, and 48 h of therapeutic cooling. After the first 200 subjects have been randomized, additional treatment arms between 12 and 48 h will be opened and patients will be allocated, within each initial cardiac rhythm type (shockable or non-shockable), by response adaptive randomization. As the trial continues, shorter and longer duration arms may be opened. A maximum sample size of 1800 subjects is proposed. Secondary objectives are to characterize: the overall safety and adverse events associated with duration of cooling, the effect on neuropsychological outcomes, and the effect on patient-reported quality of life measures.

**Discussion:**

In vitro and in vivo studies have shown the neuroprotective effects of therapeutic hypothermia for cardiac arrest. We hypothesize that longer durations of cooling may improve either the proportion of patients that attain a good neurological recovery or may result in better recovery among the proportion already categorized as having a good outcome. If the treatment effect of cooling is increasing across duration, for at least some set of durations, then this provides evidence of the efficacy of cooling itself versus normothermia, even in the absence of a normothermia control arm, confirming previous RCTs for OHCA survivors of shockable rhythms and provides the first prospective controlled evidence of efficacy in those without initial shockable rhythms.

**Trial registration:**

ClinicalTrials.gov NCT04217551. Registered on 30 December 2019.

**Supplementary Information:**

The online version contains supplementary material available at 10.1186/s13063-024-08280-w.

## Administrative information

**Table:** 

Title {1}	**Influence of Cooling duration on Efficacy in Cardiac Arrest Patients (ICECAP): study protocol for a multicenter, randomized, adaptive allocation clinical trial to identify the optimal duration of induced hypothermia for neuroprotection in comatose, adult survivors of after out-of-hospital cardiac arrest**
Trial registration {2a and 2b}	ClinicalTrials.gov: NCT04217551
Protocol version {3}	Version 1, April 14th, 2020
Funding {4}	Supported by grants: UG3HL145269,UH3HL145269,U24HL145272,U24NS100659, U24NS100655 from The National Heart, Lung and Blood Institute (NHLBI) and the National Institute Of Neurological Disorders and Stroke (NINDS) This work was supported jointly by the National Institutes of Health Common Fund (Office of the Director) and the Food and Drug Administration, with funding administered by NINDS U01NS073476
Author details {5a}	**CORRESPONDING AUTHOR** William J Meurer, MD, MS, wmeurer@med.umich.edu, University of Michigan, Emergency Medicine, Neurology, Ann Arbor, MI TC B1-354 1500 E. Medical Center Drive 48109–5301 Florian F Schmitzberger, MD, MS, fschmitz@med.umich.edu, University of Michigan, Emergency Medicine, Ann Arbor, MI Sharon Yeatts, PhD, yeatts@musc.edu, Medical University of South Carolina, Public Health Sciences, Charleston, SC Viswanathan Ramakrishnan, Phd, Ramakris@musc.edu, Medical university of South Carolina, Public health sciences, Charleston, SC Benjamin Abella, MD MPhil, benjamin.abella@pennmedicine.upenn.edu, University of Pennsylvania, Emergency Medicine, Philadelphia, PA Tom Aufderheide, MD, MS, taufderh@mcw.edu, Medical College of Wisconsin, Emergency Medicine, Milwaukee, WI William Barsan, MD, Wbarsan@umich.edu, University of Michigan, Emergency Medicine, Ann Arbor, MI Justin Benoit, MD, MS, justin.benoit@uc.edu, University of Cincinnati, Emergency Medicine, Cincinnati, OH Scott Berry, PhD, scott@berryconsultants.net, Berry Consultants,, TX Joy Black, BSN, MS, joypink@med.umich.edu, University of Michigan, ThermoFisher Scientific, Emergency Medicine, Neuroscience, Ann Arbor, MI Nia Bozeman, BA, nbozeman@med.umich.edu, University of Michigan, Emergency Medicine, Ann Arbor, MI Kristine Broglio, M.S., KRBroglio@gmail.com, Berry Consultants, Oncology Statistical Innovation, Gaithersburg MD Jeremy Brown, MD, jeremy.brown@nih.gov, National Institutes of Health, National Institute of Neurological Disorders and Stroke, Bethesda, MD Kimberly Brown, MBA, brokimbe@med.umich.edu, University of Michigan, Emergency Medicine, Ann Arbor, MI Noelle Carlozzi, PhD, carlozzi@med.umich.edu, University of Michigan, Physical Medicine and Rehabilitation, Surgery, Ann Arbor, MI Angela Caveney, PhD, angif@umich.edu, University of Michigan, Emergency Medicine, Ann Arbor, MI Sung-Min Cho, DO, MHS, csungmi1@jhmi.edu, Johns Hopkins Hospital, Anesthesia, Critical Care Medicine, Baltimore, MD Hangyul Chung-Esaki, MD, hesaki@queens.org, The Queen’s Medical Center, University of Hawaii John A. Burns School of Medicine, Critical Care, Honolulu, HI Robert Clevenger, MPH, clevenge@musc.edu, Medical University of South Carolina, Public Health Sciences, Charleston, SC Robin Conwit, MD, Rconwit@iu.edu, Indiana University, Neurology, Indianapolis, IN Richelle Cooper, MD, MSHS, RCooper@mednet.ucla.edu, UCLA David Geffen School of Medicine, Emergency Medicine, Los Angeles, CA Valentina Crudo, MD, vcrudo@med.umich.edu, University of Michigan, Emergency Medicine, Ann Arbor, MI Mohamud Daya, MD, MSc, dayam@ohsu.edu, Oregon Health & Science University, Emergency Medicine, Portland, OR Deneil Harney, MPH, MSW, dkolk@umich.edu, University of Michigan, Emergency Medicine, Ann Arbor, MI Cindy Hsu, MD, PhD, hcindy@med.umich.edu, University of Michigan, Emergency Medicine, Ann Arbor, MI Nicholas J Johnson, MD, nickj45@uw.edu, University of Washington, Emergency Medicine, Division of Pulmonary, Critical Care, and Sleep Medicine, Medicine, Seattle, WA Imad Khan, MD, imad_khan@urmc.rochester.edu, University of Rochester Medical Center, Neurology, Rochester, NY Shaveta Khosla, PhD MPH, skhosl2@uic.edu, University of Illinois Chicago, Emergency Medicine, Chicago, IL Peyton Kline, MHA, BSPH, CCRP, peyton@musc.edu, Medical University of South Carolina, Public Health Sciences, Charleston, SC Anna Kratz, PhD, alkratz@med.umich.edu, University of Michigan, Physical Medicine and Rehabilitation, Ann Arbor, MI Peter Kudenchuk, MD, pkudenchuk@cardiology.washington.edu, University of Washington, Medicine, Division of Cardiology, Seattle, WA Roger J Lewis, MD, PhD, roger@berryconsultants.net, David Geffen School of Medicine at UCLA, Emergency Medicine, Los Angeles, CA Chaitra Madiyal, M.S., cmadiyal@gmail.com, University of Michigan, Emergency Medicine, Ann Arbor, MI Sara Meyer, BS, butlers@musc.edu, Medical University of South Carolina, Public Health and Sciences, Charleston, SC Jarrod Mosier, MD, jmosier@arizona.edu, University of Arizona, Emergency Medicine, Medicine, Tucson, AZ Marwan Mouammar, MD, Mmouammar@gmail.com, OHSU Portland Adventist Medical Center, Medicine, Critical Care Medicine, Portland, OR Matthew Neth, MD, neth@ohsu.edu, Oregon Health & Science University, Emergency Medicine, Portland, OR Brian O'Neil, MD, boneil@med.wayne.edu, Wayne State University, Emergency Medicine, Detroit, MI James Paxton, MD MBA, jpaxton@med.wayne.edu, Wayne State University, Emergency Medicine, Detroit, Michigan Sofia Perez, BS, persof@umich.edu, University of Michigan, Emergency Medicine Research, Ann Arbor, MI Sarah Perman, MD, MSCE, Sarah.perman@yale.edu, Yale School of Medicine, Emergency Medicine, New Have CT Cemal Sozener, MD, MEng, cemal@med.umich.edu, University of Michigan, Emergency Medicine, Ann Arbor, MI Mickie Speers, BSN, LRAES@med.umich.edu, University of Michigan, Emergency Medicine, Ann Arbor, MI Aimee Spiteri, AAS, abspiter@med.umich.edu, University of Michigan, Emergency Medicine, Ann Arbor, MI Valerie Stevenson, BAS, vwillis@umich.edu, University of Michigan, Emergency Medicine, Ann Arbor, MI Kavita Sunthankar, BSN, MSN, patelka90@gmail.com, Medical University of South Carolina, Data Coordination Unit, Charleston, SC Joseph Tonna, MD, MS, joseph.tonna@hsc.utah.edu, University of Utah Health, Surgery, Salt Lake City, UT Scott Youngquist, MD, MS, scott.youngquist@utah.edu, Spencer Eccles School of Medicine at the University of Utah, Emergency Medicine, Salt Lake City, UT Romergryko Geocadin, MD, rgeocad1@jhmi.edu, Johns Hopkins University School of Medicine, Neurology, Anesthesiology-Critical Care Medicine, Neurosurgery, Baltimore, MD Robert Silbergleit, MD, robie@med.umich.edu, University of Michigan, Emergency Medicine, Ann Arbor, MI For the ICECAP trial investigators (names in acknowledgments section)
Name and contact information for the trial sponsor {5b}	National Institute of Health: National Heart, Lung, and Blood Institute (NIH NHLBI): National Heart, Lung, and Blood Institute, Building 31, 31 Center Drive, Bethesda, MD 20892 National Institute of Health: National Institute of Neurological Disorders and Stroke (NIH NINDS): NIH Neurological Institute, P.O. Box 5801; Bethesda, MD 20824
Role of sponsor {5c}	The study sponsors do not have any role in any trial design decisions

## Introduction

### Background and rationale {6a}

The overarching goal of this investigation is to identify clinical strategies that will improve the neurological outcomes of comatose patients after out-of-hospital cardiac arrest. We hypothesize that longer durations of cooling may improve either the proportion of patients that attain a good neurological recovery or may result in better recovery among the proportion already categorized as having a good outcome. If the treatment effect of cooling is increasing across duration, for at least some set of durations, then this provides evidence of the efficacy of cooling itself versus normothermia, even in the absence of a normothermia control arm, confirming previous randomized controlled trials (RCTs) for survivors of shockable rhythms and provides the first prospective controlled evidence of efficacy in those without initial shockable rhythms.

#### Pre-clinical data on efficacy of cooling

After resuscitation from cardiac arrest, brain neurons experience damage and ultimately death through a variety of pathophysiological pathways [1]. The processes occur differentially over several time periods and involve both immediate necrosis and apoptosis. Clinically, in humans rapidly resuscitated from cardiac arrest, neuronal injury from brief ischemia and reperfusion tend to lead to damage that predominates through the apoptotic pathway. As such, a therapeutic window exists for neuroprotection in ischemic brain injury states such as global cardiac arrest.

In preclinical models of both global and focal ischemia, hypothermia is consistently one of the most effective treatments to reduce neuronal damage. In seminal work on this subject, rats were subjected to intra-ischemic brain temperatures of 36, 33, and 30 °C [2]. Release of glutamate and dopamine were substantially reduced, without affecting ischemia-induced cerebral blood flow reduction or free fatty acid accumulation. In a systematic review of various neuroprotectant strategies for focal ischemia in the preclinical space (the majority addressing drugs or biologics), hypothermia performed exceedingly well, and was one of only three treatments, out of 1026, to receive a perfect 10 on the Stroke Treatment Academic Industry Roundtable (STAIR) quality score [3].

The overall preclinical evidence on hypothermia for neuroprotection is extremely (perhaps uniquely) robust. An exhaustive review in 2006 reviewed preclinical data from 1026 experimental treatments for ischemic brain injury [3]. The authors compiled 7554 experimental results from 3500 papers. Hypothermia was the most thoroughly studied intervention, having been evaluated for efficacy in 244 studies, 105 of which were models of global cerebral ischemia (with the others being models of focal ischemia or hypoxia-glucose deprivation in cell culture). Hypothermia had the highest STAIR score of any neuroprotective strategy reflecting the reproducibility of efficacy across models, species, outcome metrics, and severity of injury. Preclinical investigations of hypothermia in cerebral ischemia have continued at a high rate with a PubMed search of “hypothermia cerebral ischemia” limited to animal investigations demonstrating an average of 58 publications per year since 2003, the end of the search period included in the 2006 review. Despite the robust study of hypothermia in animal models, the experimental space dedicated to the effects of varying durations of therapy are limited, largely due to the difficulty of clinically realistic modeling of multiple days of intensive care.

#### Pre-clinical data on duration of cooling

Preclinical models of global cerebral ischemia demonstrate that neuroprotection has a dose response with increasing efficacy with longer durations of hypothermia, and suggest potential mechanisms of benefit to explain this effect. Previous work compared 12 h of hypothermia versus 24 h in a gerbil model of 5 min of global cerebral ischemia and evaluated hippocampal CA1 cell counts at 30 days [4]. Animals were cooled to 32° and cooling was initiated 1 h after the period of ischemia. They demonstrated dramatically greater neuronal protection versus untreated controls (90%) with longer duration of hypothermia compared to the neuronal protection seen with the 12-h duration (15%). In a subsequent study, this group demonstrated that the histopathological findings in this model reflected behavioral deficits with 24 h of cooling even with initiation of therapy at either 1 or 4 h post ischemia [5]. In 2011, Che compared 24 to 48 h of hypothermia in a rat model of global cerebral ischemia from 10 min of cardiac arrest [6]. Cooling was initiated at 0, 1, 4, or 8 h after ischemia and animals were cooled to 33°. Hippocampal CA1 cell counts at 7 days in this model of more severe injury again showed improved neuronal preservation with longer durations of hypothermia, with 68% (+ / − 15%) preservation at 48 h compared to 42% (+ / − 22%) at 24 h (*p* < 0.0001).

It is less clear whether the duration response curve seen in these two studies between 12 and 24 h and 24 and 48 h also exists over much shorter (less clinically relevant) durations of hypothermia. Ye et al. compared 2, 5, and 8 h of cooling to 33° initiated 7 min after an 8-min cardiac arrest in a rat model and found no duration response in behavioral outcomes [7]. However, Zhang et al. compared 0.5, 1, 2, and 4 h of cooling to 32° initiated immediately after 20 min of 4-vessel occlusion in a rat model and found robust duration response on oxidative and cytokine markers of injury [8]. Unfortunately, both experiments only recovered for short durations and neither obtained histological outcomes, so only limited conclusions can be drawn.

Improved neuroprotection with increasing duration of hypothermia at 12, 24, and 48 h is reproducible across models of transient or permanent focal cerebral ischemia [9]. Benefit from prolonged durations of 48 h of hypothermia has also been confirmed in focal cerebral ischemia in aged rats [10]. Benefit was seen in anatomic, histopathologic, biochemical, and behavioral outcomes across these models.

Yenari et al. have speculated on the mechanisms for enhanced neuroprotection with prolongation of hypothermia and suggest that even longer durations may be needed to optimize recovery [11]. They note that in both global and focal models of cerebral ischemia there is an increase in neuronal neurogenesis when hypothermia is given for 24 h, but that this effect is not present in models of short durations of cooling. In rats with global forebrain ischemia, Silasi et al. reported a 60% increase in the number of BrdU/NeuN-positive dentate gyrus neurons at 4 weeks in rats receiving 24 h of hypothermia relative to normothermic rats (*p* < 0.0001) [12]. Similarly, Xiong et al. demonstrated neurogenesis, evidenced by significantly increased BrdU + stained immature and mature neurons at 2 weeks, after 24 h of hypothermia in a rat model of focal cerebral ischemia as compared to controls [13]. In contrast, in the rat global forebrain ischemia model, Lasarzik et al. found no evidence of alteration of post-ischemic neurogenesis on BrdU staining at 4 weeks in animals cooled to 33° for only 45 min, as compared to normothermic controls [14]. Increased efficacy with prolongation of hypothermia could be mediated by these and other regenerative mechanisms including not only neurogenesis, but neuronal connectivity, angiogenesis, and gliogenesis [11].

#### Clinical trials in humans

A number of large RCTs have evaluated therapeutic hypothermia and target temperature management (the latter broader term encompassing the use of advanced temperature management to enforce low normal targets or hypothermia) following cardiac arrest. The first two, utilizing surface cooling in ventricular fibrillation/pulseless ventricular tachycardia patients with OHCA (out of hospital cardiac arrest) were published in 2002. The Hypothermia After Cardiac Arrest (HACA) trial was a multicenter trial of cooling versus no cooling in 273 comatose survivors of out of hospital cardiac arrest [15]. HACA demonstrated improved neurological outcomes (55% versus 39%—*P* = 0.009) in the group receiving hypothermia to 33 °C for 24 h versus a group with no temperature control as measured by the Cerebral Performance Score of 1 or 2 at 6 months. In the same issue of the New England Journal of Medicine, a similar, smaller trial of 77 subjects by Bernard in Australia demonstrated a 49% rate of good neurological outcome in patients receiving hypothermia to 33 °C for 12 h as compared to 26% in the normothermic control group [16].

The Targeted Temperature Management (TTM) trial was a large, randomized controlled trial performed nearly 10 years later. TTM randomized OHCA patients with presumed cardiac etiology to a target of either 33 or 36 °C [17]. The target of 36 °C was chosen to avoid re-warming patients who usually presented to the ED with nominally lower body temperatures following cardiac arrest, and to prevent patients from developing hyperthermia which has previously been demonstrated to likely be injurious in numerous observational and animal studies. In addition, both treatment groups in this two-arm trial were exposed to an excellent prognostication protocol that provided safeguards against premature withdrawal of life support in potentially salvageable individuals. This extremely well conducted and conceived trial had 939 patients included in the final analysis; about a quarter had temperature management with an endovascular device (as this was left to the discretion of sites). About half of the patients in both groups had a favorable neurological outcome (measured by either the modified Rankin Scale (mRS) or the CPC) at 180 days. This finding closely matched the observed outcomes in the cooled groups of the HACA and Bernard trials, although the TTM trial included about 20% patients with non-shockable rhythms of presumed cardiac origin, and excluded those with shockable rhythms but not presumed to be of a cardiac cause. In this large trial, the safety of both regimens was effectively identical (of pre-specified serious adverse events, only hypokalemia was observed in a higher proportion of the 33 °C group). To many, the 36 °C group resembles normothermia, and the lack of benefit compared to 33° is interpreted as lack of overall benefit from cooling beyond using advanced temperature control devices to prevent hyperthermia. To many others, however, using advanced cooling devices to maintain a target of 36° is still cooling, albeit to a higher temperature (a lower dose of cooling). In this context, TTM is interpreted as showing that two doses of hypothermia are equally effective. This has reinforced the importance of having another study like ICECAP to more robustly confirm efficacy of cooling or to restore sufficient uncertainty in the larger clinical community to permit a future trial with a normothermic control arm.

Recently (subsequent to the funding of ICECAP), the HYPERION trial enrolled 584 comatose survivors of cardiac arrest from non-shockable rhythms and randomized to treatment with 24 h of targeted temperature management at 33 °C versus 37 °C in 25 French ICUs. This trial found a clinically and statistically significant improvement in favorable neurologic outcome in the 33 °C group (10.2%) as compared to the 37 °C group (5.7%), assessed on day 90 after randomization with the use of the Cerebral Performance Category (CPC) scale. These findings buttress the inclusion of patients with non-shockable rhythms cooled as part of standard care in the ICECAP trial and reinforce the need to confirm the efficacy finding and seek dose optimization in this patient cohort in the current trial [18].

The Targeted Hypothermia versus Targeted Normothermia after Out-of-Hospital Cardiac Arrest (TTM2) trial, published in 2021, is a very large comparison of outcomes in normothermia vs. hypothermia after OHCA, regardless of initial rhythm presentation [19]. It enrolled 1861 patients, 930 assigned to the hypothermia group with a target temperature of 33 °C, and 931 assigned to the normothermia group, with the aim of a temperature not higher than 37.5 °C. If the temperature in the normothermia group reached higher than 37.8 °C, a cooling device was implemented to return to the target temperature of 36.6 to 37.7 °C. Patients in the hypothermia group were kept at 33 °C for 28 h, with a median time to target temperature of 3 h; they were then rewarmed gradually to 37 °C in hourly increments of one third of a degree. After the intervention period, a normothermic target (36.6 to 37.7 °C) was maintained for 72 h after randomization. At 96 h or later after randomization, a physician blinded to the intervention performed a neurological assessment in patients that were still in the ICU, to evaluate criteria for a potentially poor neurological outcome. The primary outcome was death from any cause at 6 months, and the main secondary outcome was poor functional outcome, defined as a score of 4 to 6 on the mRS.

The study did not find a significant difference in the two groups, in death at 6 months or neurological performance as evaluated by the mRS. The hypothermia group showed a statistically significant higher rate of arrhythmias causing hemodynamic compromise compared to the normothermia group. The TTM2 trial was performed on a large sample of patients, to reach a power of about 90%, and its results, like those of the TTM trial, are of relevance. It is however debated whether the normothermia group can be truly considered such, since about 50% of patients in this group used a cooling device to maintain the target “normal” temperature. A third group with no temperature management was not included.

**Fig. 1:**
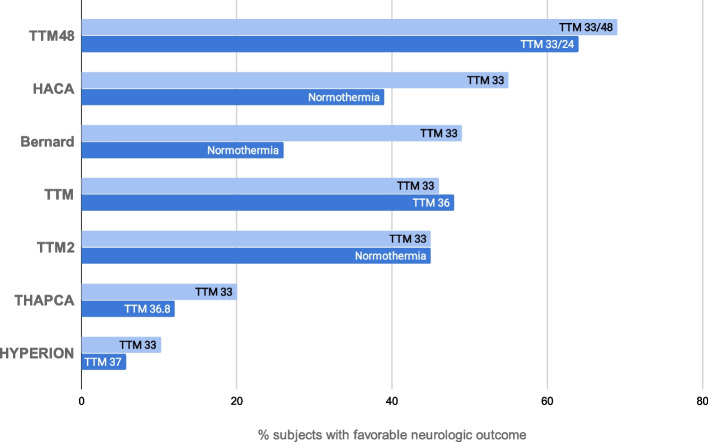
Summary of Human Cardiac Arrest Trials comparing duration of cooling with favorable neurologic outcome

Figure 1 gives a summary of human cardiac arrest trials.

#### The ICECAP design in context of current knowledge

Prior clinical trials have created a sometimes confusing, sometimes nihilistic context relevant to ICECAP. The HACA and Bernard trials published in 2002 compared 33 °C to normothermia and showed marked efficacy of cooling but had methodological flaws [16]. The European TTM trial published in 2015 compared 33 to 36 °C and found that 36 was neither more or less effective [17]. Outcomes in both arms were similar to the cooling arms in the prior trials. TTM also showed that 36 was neither safer nor easier than 33 °C. Nevertheless, TTM resulted in some clinicians rejecting the 33 °C target, but changes in practice using higher target temperatures have sometimes been problematic and associated with worse outcomes in observational studies [20]. The impact of HYPERION is not clear as this was just published. Two other related trials have also affected understanding. Into this milieu, THAPCA, a pediatric OHCA study comparing 33 to 36.5 °C was also published in 2015 [21]. THAPCA was a neutral study despite estimating an 8% absolute (66% relative) higher rate of survival with good neurological outcomes in the 33 °C arm. The TTM48 trial, published in 2017, compared 33 °C for 24 to 48 h [22]. TTM48 demonstrated outcomes far better than prior trials in both groups but also estimated 7% better survival and 5% better neurological outcomes in the longer 48-h arm, with no difference in adverse event rates. Published in 2021, TTM2 concluded that targeted hypothermia did not lead to a lower incidence of death by 6 months than targeted normothermia.

All of this has evoked confusion and frustration in the clinical community. Clinicians are left to wonder if depth of cooling is even important, and whether nothing ever works, or whether the trials are all just underpowered to detect meaningful differences. To the first question, we conclude that trials have found 33 to be as good or better than their control arms, such that it remains a promising standard target to be used in ICECAP. Despite a completed TTM2 trial, alternative depths are unlikely to prove scientifically or clinically impactful in the long run. To the latter question of nihilism, we offer a smarter study designed to be convincing and not ambiguous, regardless of the direction of its findings.

### Objectives {7}

ICECAP has two primary objectives, each evaluated in two populations of adult comatose survivors of cardiac arrest (those with initial shockable rhythms and those with PEA/asystole). First, to determine the shortest duration of cooling that provides the maximum treatment effect (good neurological recovery) as determined by a weighted 90-day mRS. Second, to determine whether increasing durations of cooling are associated with better outcomes or recovery implying efficacy of hypothermia to no cooling.

The secondary objectives of this project are to characterize:

- The overall safety and adverse events associated with duration of cooling
- The effect of duration of cooling on neuropsychological outcomes
- The effect of duration of cooling on patient-reported quality of life

### Trial design {8}

This study is a randomized, response-adaptive, duration (dose) finding, comparative effectiveness clinical trial with blinded outcome assessment. The design is based on a statistical model of response as defined by the primary endpoint, a weighted 90-day mRS, across the treatment arms. The design will fit patient outcome data to a duration response model (separately for shockable and non-shockable rhythms), in which the potentially non-linear association between durations of cooling and the primary endpoint are estimated. All conclusions about the treatment arms are based on this model. The functional form of the duration-response model is flexible and able to fit many different shapes for the duration-response curve. Specifically, it is parameterized to identify up to two change-points in the treatment effect across arms, allowing it to fit an increasing, decreasing, flat, plateau, or *U*-shape duration-response curve.

Subjects will initially be equally randomized between 12, 24, and 48 h of cooling. After the first 200 subjects have been randomized, additional treatment arms between 12 and 48 h will be opened and patients will be allocated, within each rhythm type, by response adaptive randomization. As the trial continues, shorter and longer duration arms may be opened. Specifically, a 6-h duration arm will be opened if the emerging duration-response curve from 12 h is flat. Similarly, a 60-h or 72-h duration arm will be opened if the emerging duration response curve shows an increasing treatment benefit through 48 h.

This trial will have frequent interim analyses to stop the trial early for futility if it is highly likely that no treatment arm offers a greater benefit than the 6-h duration arm.

## Methods: participants, interventions and outcomes

### Study setting {9}

The ICECAP trial is conducted by the Strategies to Innovate Emergency Care Clinical Trials (SIREN) Network (siren.network). The United States National Institutes of Health (NIH) provides funding for SIREN. Only sites within the USA are included. The sites include academic and community hospitals. After activation sites will be listed at http://clinicaltrials.gov. In addition, the study website (http://icecaptrial.org) lists the sites. The study was institutional review board (IRB) approved by Advarra (Columbia, MD), CR00412243, protocol Pro00041076.

### Eligibility criteria {10}

Sites are eligible if they are using servo-controlled devices (with an integrated temperature sensor) to deliver TTM to patients following cardiac arrest as usual care.

To be eligible, comatose adult survivors of OHCA must have already been rapidly cooled using a definitive temperature control method (endovascular or surface) within 4 h of cardiac arrest. These patients will be enrolled in the emergency department (ED) or intensive care unit (ICU).

#### Inclusion criteria

- Coma after resuscitation from out-of-hospital cardiac arrest
- Cooled to < 34 °C within 240 min of cardiac arrest
- Definitive temperature control device applied
- Age ≥ 18 years
- Informed consent from the patient’s legally authorized representative (LAR), including intent to maintain life support for 96 h
- Enrollment (randomization) within 6 h of initiation of cooling

#### Exclusion criteria

- Hemodynamic instability (systolic blood pressure < 80 mm Hg despite aggressive management)
- Pre-existing neurological disability or condition that confounds outcome determination
- Pre-existing terminal illness, unlikely to survive to outcome determination
- Planned early withdrawal of life support
- Presumed sepsis as etiology of arrest
- Prisoner

## Patient consent

### Who will take informed consent? {26a}

Trained study personnel will obtain informed consent to participate in the study. Because eligible patients for this study will be comatose and unable to consent to participate, the informed consent process will be conducted with the patient’s LAR as defined by prevailing local law or regulation. During this process, the LAR will receive a verbal explanation (in a language with which they have sufficient fluency) of the purpose of the study, the scientific basis for hypothermia as a neuroprotectant, the randomization process, the process of temperature management, and the follow-up examinations required. The specific risks of participating will be outlined. The LAR will be informed that the optimal duration of hypothermia has not yet been determined and that participation is completely voluntary and that declining to participate will not adversely affect their loved one’s care. All patient questions and concerns will be answered and addressed. Those choosing to enroll their loved one will sign a written informed consent document (paper or e-consent).

The LAR is also the surrogate decision maker in clinical practice regarding choices related to the timing of withdrawal of life support in the days following resuscitation. The timing of withdrawal of life support is a major confounder in the evaluation of duration of cooling after cardiac arrest. To reduce variability from this issue, the timing of potential withdrawal of life support under relevant scenarios must be discussed prior to enrollment, typically as part of the consent process. Only those patients whose LAR has indicated, as part of the informed consent process, intent to maintain life support for 96 h are enrolled.

### Additional consent provisions for collection and use of participant data and biological specimens {26b}

The overall consent form and process is inclusive of data collection. No biological specimen collection is part of the main ICECAP trial. Ancillary studies collecting additional biological specimens are anticipated.

## Interventions

### Explanation for the choice of comparators {6b}

#### Why study therapeutic hypothermia targeted to 33°?

Meta-analyses of clinical trials and observational studies of induced hypothermia in comatose survivors of cardiac arrest are largely in line with preclinical data [23, 24]. These analyses conclude that the accumulated human data are consistent with both neuroprotection and improved survival in comatose survivors of OHCA treated with therapeutic hypothermia, even when including patients with non-shockable initial presenting rhythms. Specifically, Schenone et al. found that use of therapeutic hypothermia “decreased the mortality (OR 0.51, 95%CI 0.41–0.64) and improved the odds of good neurological outcome (OR 2.48, 95%CI 1.91–3.22)” [24]. There are also corroborative human data from meta-analysis of clinical trials of therapeutic hypothermia in neonates with hypoxic ischemic encephalopathy, a pathology similar to that seen after cardiac arrest [25]. Cooling was associated with reduced mortality and less major disability. Although these analyses include observational and inferential data, taken together the existing data strongly support including patients with all presenting rhythms in a randomized trial large enough to directly address, rather than add to, uncertainty.

Some other specific observations about prior clinical trials of therapeutic hypothermia are important because they have created an often confusing and sometimes nihilistic context for the current proposal with the TTM2 trial adding additional uncertainty. Some trials identify cooling as “therapeutic hypothermia” and others as “target temperature management” with the latter broader term encompassing the use of advanced temperature management to enforce low normal targets or hypothermia.

#### Mechanisms of secondary neuronal injury provide rationale for importance of duration

Mechanistic experiments have elucidated many pathways by which hypothermia may act to reduce neuronal loss or promote recovery. Reviewing this work, Yenari and Han developed an illustrative map of the ischemic cascade that relates when various systems become most relevant after ischemia and which of these systems appear to be modified by hypothermia [26]. They demonstrate that many of the putative protective mechanisms by which hypothermia may act, especially apoptosis and inflammation, peak days after ischemia and reperfusion. Other mechanisms like neuro-, angio-, and gliogenesis only arise after several days and could only be potentially modified with prolonged experimental durations of cooling.

### Intervention description {11a}

#### Interventions, administration, and duration

**Fig. 2:**
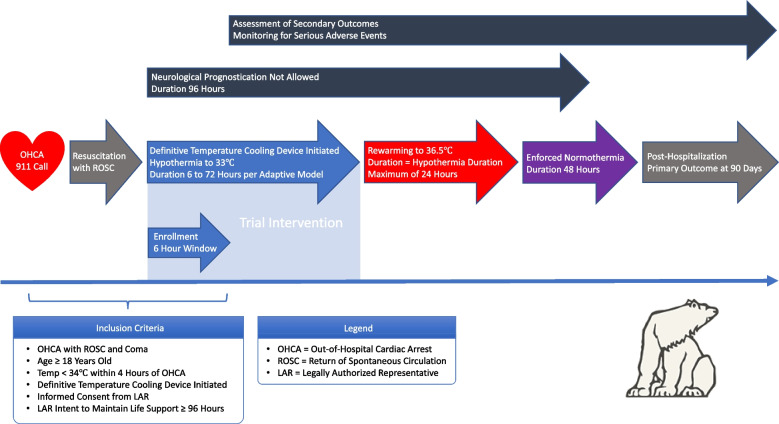
Per-patient inclusion and timeline in the trial

The intervention will be random allocation to duration of cooling after cardiac arrest. Figure 2 shows the per-patient inclusion in the trial. Cooling in the study will be by a definitive temperature control method to a target temperature of 33 °C. Any endovascular or surface cooling system with closed loop feedback will be allowed. Duration of cooling will be measured from the time that definitive cooling is initiated in the hospital, as indicated by placement and activation of a definitive cooling device. Cooling by other means may be initiated by EMS (emergency medical services) or in the emergency department as per local protocols for standard care. Eligibility will require that a temperature of < 34 °C be obtained by 240 min after the index cardiac arrest. After the allocated duration of cooling is completed, controlled rewarming will be performed. Rewarming to a temperature of 36.5 °C will occur over the shorter of 24 h or a rewarming period equal to the allocated duration of cooling. Definitive cooling devices may be used for maintenance of normothermia after rewarming is complete.

#### Definition of definitive device

Definitive device is defined as a closed loop feedback endovascular or surface cooling device that can be used to both induce and maintain therapeutic hypothermia.

### Criteria for discontinuing or modifying allocated interventions {11b}

#### Temporary cessation of cooling

In certain instances it may be necessary to disconnect the subject from the definitive cooling device such as during patient transport to and from diagnostic or therapeutic procedures. Interruptions in active temperature management should be minimized but brief periods of less than 1 h are allowed as required. For longer periods of potential interruption, the definitive cooling device should accompany the patient and be re-instituted during the procedure to avoid temperature excursions. Core temperatures should be documented every 30 min during interruptions in cooling.

#### Cessation of cooling

TTM should only be discontinued if the patient is waking up and following commands. The clinical team is discouraged from modifying the target temperature (33 °C) prior to the assigned duration, but can do so if a compelling clinical need exists.

### Strategies to improve adherence to interventions {11c}

The clinical monitoring team at the Clinical Coordinating Center (CCC) will review the temperature logs for adherence to the protocol. In addition, excursions from clinical standardization parameters (electrolytes, glucose) will be logged for each patient. The CCC will also evaluate other quality areas such as the timing and content of neurological prognostication. The trial monitors and leadership will give feedback to clinical sites. If necessary, sites will develop corrective action plans for repeated non-adherence to the protocol.

### Relevant concomitant care permitted or prohibited during the trial {11d}

Overall, the clinical teams may use any procedures or medications indicated for the treatment of the study participants. The clinical teams are strongly discouraged from altering the target temperature or duration.

#### Clinical standardization

A clinical standardization guideline will be followed to reduce the effects of practice variability subsequent to randomization. Key physiologic and practice variables will be tracked. Compliance with clinical standardization and deviation from physiologic targets will be reported back to study teams. Clinical standardization guidelines will include but may not be limited to avoiding hypotension, avoiding hypoxia, controlling rebound hyperthermia, treatment of seizures, treatment of shivering, management of sedation and paralysis, prognostic testing, and defining and treating infections. Clinical standardization guidelines define that neurologic prognostication leading to withdrawal of life support is only allowed after 96 h. Details related to neurological prognostication are provided in the clinical standardization guidelines.

### Provisions for post-trial care {30}

The ICECAP trial does not provide for post trial care. If additional medical care is required due to complications from temperature management, this will be managed as per local site procedure for routine ICU care. The ICECAP study will not provide compensation for injury attributable to the protocol, although participants and families retain all legal rights.

### Outcomes {12}

#### Primary efficacy outcome

The primary outcome measure will be the mRS at 90 days after return of spontaneous circulation. The mRS will be analyzed as a weighted score incorporating both the proportion of subjects achieving a good neurological outcome and degree of residual functional impairment among those with good neurological outcomes. The mRS will be determined primarily by a central assessor at the CCC by telephone or telepresence. In addition, this assessor must be blinded to the treatment group assignment for the subject.

#### Safety outcomes

The primary safety outcome is all cause mortality at 90 days. All-cause mortality is selected because it incorporates most severe irreversible safety consequences across many potential adverse events. Safety problems that are not reflected in either neurological recovery (the efficacy outcome measure) or mortality (the primary safety measure) do not generally reflect any permanent morbidity and are therefore secondary.

Secondary safety outcomes include active monitoring for serious adverse events (SAEs) throughout the trial. Specific SAEs are anticipated to be related to therapeutic hypothermia. These selected SAEs include pneumonia, other infections (including urinary tract infections and bacteremic sepsis), malignant cardiac arrhythmia (cardiac arrest, ventricular fibrillation, ventricular tachycardia, atrial arrhythmias with hemodynamic compromise), seizures, neurological worsening, electrolyte abnormalities, venous thrombotic disease, and coagulopathies. The occurrences of these safety outcomes by treatment arm will be reported in the periodic safety reports to the Data and Safety Monitoring Board (DSMB). We will also report counts and proportions of mortality for each treatment arm (by rhythm), along with the number of SAEs that are probably or definitely related to intervention.

#### Secondary efficacy measures—patient-reported outcomes

Quality of Life in Neurological Disorders (Neuro-QoL) is a set of self-report measures that assesses the health-related quality of life (HRQOL) of adults and children with neurological disorders.

Neuro-QOL consists of item banks and scales that evaluate symptoms, concerns, and issues that are relevant across disorders—along with measures that assess areas most relevant for specific patient populations.

The Neuro-QoL tool includes carefully developed and rigorously calibrated comprehensive item banks of patient-reported outcomes that are relevant to people with neurological disorders. The item banks include: Physical Health (e.g., Mobility; Fine Motor/ADL; Fatigue; Sleep Disturbance), Social Health (Ability to Participate in Social Roles & Activities; Satisfaction with Social Roles & Activities), Emotional Health (e.g., Depression, Anxiety, Stigma, Positive Affect & Well-Being; Emotional-Behavioral Dyscontrol), Cognitive Health (i.e., Cognitive Function; Communication).

Item pools for the Neuro-QoL measurement system were developed through a process of engaging patients and other stakeholders (e.g., medical providers) to identify possible domains and items of interest/importance through focus groups, individual interviews, and survey research. Existing items were identified, evaluated, and revised from existing items from the published literature. New items were written to fill identified construct gaps. Items were classified into domain-specific bins for conceptual and organizational purposes. Items were reviewed and revised using patient perspectives (e.g., cognitive interviews) and stakeholder judgment (expert item review) to assure understanding, relevance, and clarity. The process also included comprehensive cultural/linguistic review of items to ensure ease of translatability, universality of concepts and clarity of phrasing, and multi-step comprehensive translation of items into Spanish language.

#### Secondary measures—neuropsychological outcomes

Neuropsychological (NP) testing provides an opportunity to examine, with great sensitivity, potentially subtle but meaningful differences in outcomes between treatment groups.

The measures chosen include focused traditional measures that have proven reliability and validity for use in trials of patients with cardiac arrest [27]. In addition, we have selected measures that comprise the cognitive domain of the NIH Toolbox [28]. This particular combination of tests is designed to capitalize on both the advantages of using traditional paper and pencil tests as well as those advantages unique to the NIH Toolbox tests; including computerized administration (which allows precise and reliable timing), the availability of characterized composite scores, and the anticipation that the Toolbox cognitive battery will be commonly utilized in future neurological trials allowing for cross trial comparisons and aggregation of trial results.

Furthermore, this particular combination of tests has been carefully designed to be comprehensive, with special emphasis on measures of domains that have been found to be most significantly impacted in previous studies of cardiac arrest, namely learning, memory, attention and executive functioning [29, 30]. Select traditional paper and pencil tests have been chosen to both supplement and complement the standard NIH Toolbox measures. Specifically, the standard NIH Toolbox includes measures of episodic memory, executive functioning (specifically flexibility and inhibition), vocabulary comprehension, reading, processing speed, and working memory. The traditional paper and pencil measures, including Trail Making Test (attention and executive functioning, flexibility) and Stroop Test (executive functioning, inhibition), were chosen to complement the newer NIH Toolbox tests in domains of particular interest. Likewise, traditional tests, including the Rey Auditory Verbal Learning Test (verbal memory) and the Controlled Oral Word Association Test (verbal fluency), were chosen because these particular domains are not tested via the NIH Toolbox.

The NIH Toolbox tests can be subdivided into crystallized (i.e., general knowledge base) and fluid (i.e., thinking and reasoning) measures, providing information about both patients’ premorbid and current functioning. A fluid composite score will be obtained for fluid measures (i.e., those expected to change with injury). A stability composite score will be calculated for crystallized measures (i.e., those not expected to change with injury). The use of two distinct composite scores rather than combining all into a single composite measure will result in both greater sensitivity of the fluid composite as well as provide us with a separate estimate of premorbid functioning.

Neuropsychological testing has been limited to 1 h to enhance patient compliance and minimize patient fatigue. Patients who cannot tolerate the complete battery of tests and interviews in one session may be scheduled for a second session. Study participants will be evaluated 90 days following randomization. Study team members responsible for neuropsychological outcome assessment will be trained and certified per study procedures.

### Participant timeline {13}

Out-of-hospital cardiac arrest patients arrive in the emergency department where routine care is performed, which includes initiation of cooling to target within 4 h from the cardiac arrest. The study team is activated to assess eligibility by inclusion and exclusion criteria. If eligibility has been established, next of kin are contacted to obtain consent and the patient may be enrolled in the study if enrollment criteria are met. The patient’s cooling duration is randomized per protocol. Figure 2 gives an overview of the participant timeline.

### Sample size {14}

This trial will enroll a maximum of 1800 patients. Interim analyses for futility will begin when about 200 subjects have been enrolled and will be conducted every 50 subjects, or about monthly. If the trial is not stopped early for futility, it will continue to enroll to the maximum sample size. Extensive numerical simulations of the design were conducted over a range of potential scenarios to characterize the trial’s type I error and the power for the primary analysis provided by a maximum of 1800 patients. Sensitivity of operating characteristics to a range of sample sizes was also simulated.

There are two components of the primary analysis and we define power for each. The following procedure is used to define power as related to the selection of the target duration (objective A). For each simulated scenario, we define up to three durations as clinically accurate selections. These include the duration set in the scenario input as the shortest duration that achieves the maximum treatment effect and up to two more durations that are clinically very similar. To be considered sufficiently similar these durations must be within 12 h of the set optimal duration and must achieve at least 70% of the maximum treatment effect. We define power for this component of the primary analysis as the probability that any one of these three clinically acceptable durations is selected as the target duration. The following procedure is used to determine if the efficacy of cooling versus no cooling is implied (objective B). For each simulated scenario, we test whether the treatment effect for any duration is greater than for a shorter duration. In certain situations, the design may have convincing evidence of duration response, but may not be able to definitively choose a duration (e.g., a gently upsloping with plateau scenario). Conversely, the design may be able to choose a target duration, but may not be able to definitively demonstrate duration response (example: true target duration 12 h, but end trial results are insufficient to declare 12 h is superior to 6 h). We define power for this objective as the probability of concluding that there is a positive duration response curve in the simulated scenarios in which the scenario input includes any increase in treatment effect with increasing duration, regardless of the target duration and whether it is correctly selected.

Our reference scenario assumes a modest benefit of cooling at 18 h, followed by a plateau in the treatment effect through 72 h. This reference scenario is based upon conservative interpretation of the two randomized controlled trials that provide the basis for current therapeutic recommendations. These trials used 12 and 24 h durations of cooling respectively to achieve absolute increases of 16–23% in the proportion of patients with a good neurological outcome after cardiac arrest with an initial shockable rhythm compared to controls without cooling. In the reference scenario, we assume an approximate 16% treatment effect for both shockable and non-shockable rhythms. The assumed treatment effects for the reference scenario are detailed in the trial design and simulation report in the protocol appendix. In this scenario, the target duration is 18 h, but 24 and 30 h would also be considered clinically acceptable. They are each within 12 h of the target duration arm and offer the same treatment effect. With a maximum of 1800 patients, assuming 50% are in each rhythm type, this trial will select one of the three clinically acceptable target durations with 70% probability and will determine that the duration-response curve is positive with 31% probability. This trial will open enrollment to the 6 h duration arm with 58% probability and will stop for futility with only 3% probability.

Sensitivity of the power to changes in maximum sample size was determined by simulation of the reference scenario and four additional variations of the reference, altering target duration and rhythm type balance, for maximum sample sizes ranging from 1500 to 2300. In the reference scenario, the power for selection of duration at a maximal sample size is 80%, and the power for determination of a positive duration response is 77%. Variation in the operating characteristics with sample size was modest, and 1800 was selected as the most practicable maximum sample size that achieved approximately 80% or better power.

### Recruitment {15}

Hub and spoke hospitals from the SIREN network will be enriched with high-potential ancillary Hubs, including some former Resuscitation Outcomes Consortium sites. During planning, approximately 50 hospitals were anticipated to each enroll an average of 9 subjects per year. The enrollment period is anticipated to be 4 years (estimated accrual rate of 38 subjects per month).

## Assignment of interventions: allocation

### Sequence generation {16a}

**Fig. 3:**
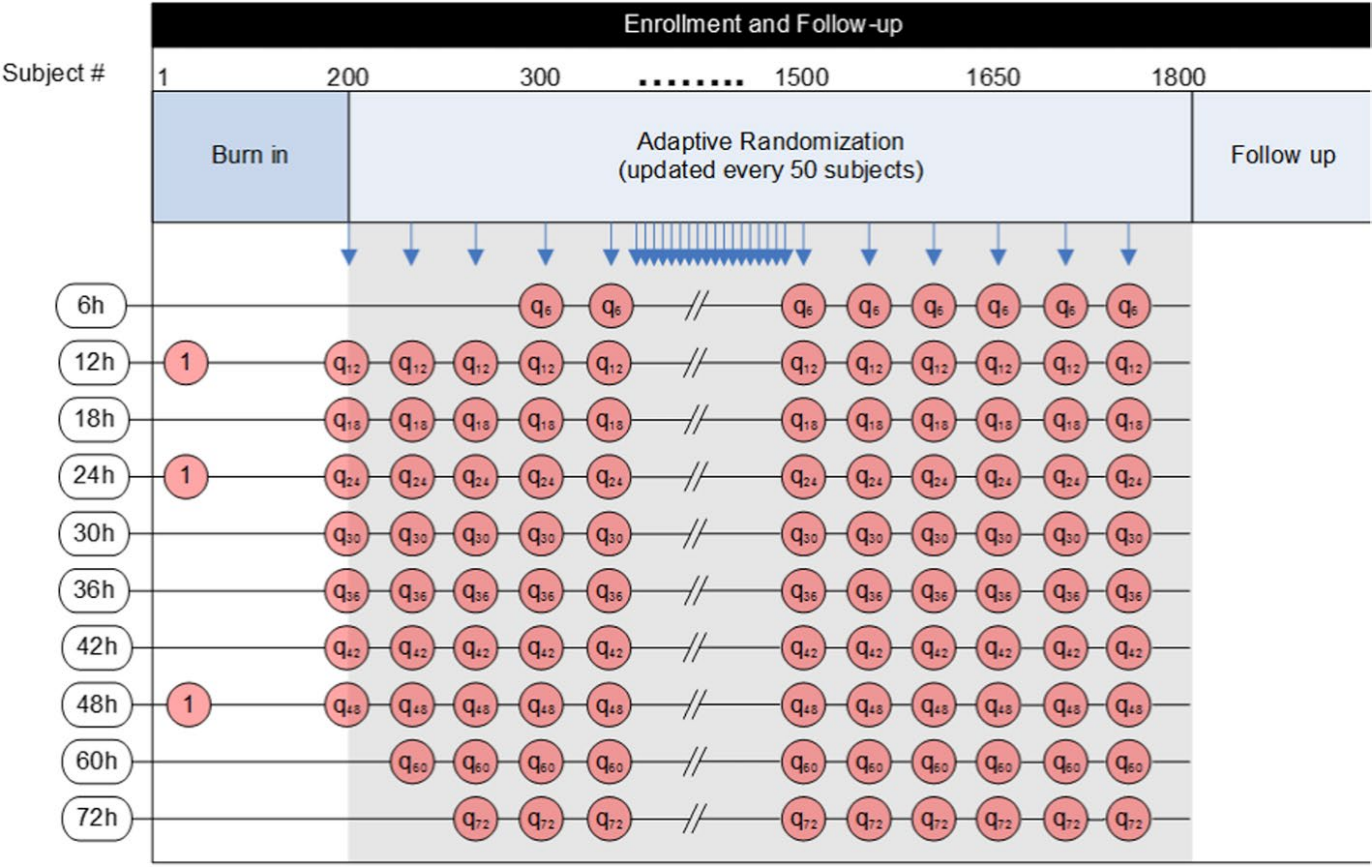
Sequence generation for adaptive randomization

Figure 3 shows the possible durations that will be tested in white boxes on the left side. They range from 6 to 72 h. The sequential columns of red circles represent progressive time and accrual in the trial. You can see that there are 3 arms in the burn-in period each with 1:1:1 allocation (each arm equally likely at one third each), but that shorter, longer, and interspersed arms are potentially added incrementally. The blue arrows at the top indicate pre-planned interim analyses about every 4 weeks or 50 subjects. At each look, a new batch of randomization vectors is assigned. If the 6-h window has opened, then there is the potential to stop for futility in that rhythm type.

### Concealment mechanism {16b}

Interventions are assigned when enrolling patients and are automatically reported by the web-based clinical trial management system WebDCU™.

### Implementation {16c}

A web-based Randomization Module in WebDCU™ will be used to randomize eligible patients. A study team member will log onto the web-based system using a unique username and confidential password. When a subject is deemed eligible, WebDCU™ will generate a unique subject ID without storing any personal identifying information. The study team member will then enter the required subject information, including presenting rhythm and inclusion/exclusion criteria. The computer program will check for accuracy and completion of this information prior to selecting the intervention assignment for that subject based on current randomization vectors. The subject is considered randomized at the time that WebDCU™ generates the study intervention assignment. An automatic email notification of randomization will be sent to the appropriate parties (e.g., the ICECAP study leadership, the NIH Program Officers, and the CCC and Data Coordinating Center (DCC) staff). If, under rare circumstances, the web system is not available, a call to the emergency randomization hotline to obtain a randomization assignment is possible.

## Assignment of interventions: blinding

### Who will be blinded {17a}

The primary outcome assessment in this trial will always be performed by a study team member blinded to treatment. Subjects themselves will be comatose during the intervention period. It is not practicable to blind the clinical care team or the subject’s family to the duration of cooling. Study procedures to prevent inadvertent unblinding include minimized contact between study team members involved in the study intervention and those performing follow-up at 3 months through the use of centralized outcomes assessment. Subjects and their family members will be instructed not to communicate any knowledge of the treatment group to the person assessing outcomes at any follow-up visit.

### Procedure for unblinding if needed {17b}

No mechanism for unblinding of the study team members performing primary outcome assessments is established.

## Data collection and management

### Plans for assessment and collection of outcomes {18a}

**Fig. 4:**
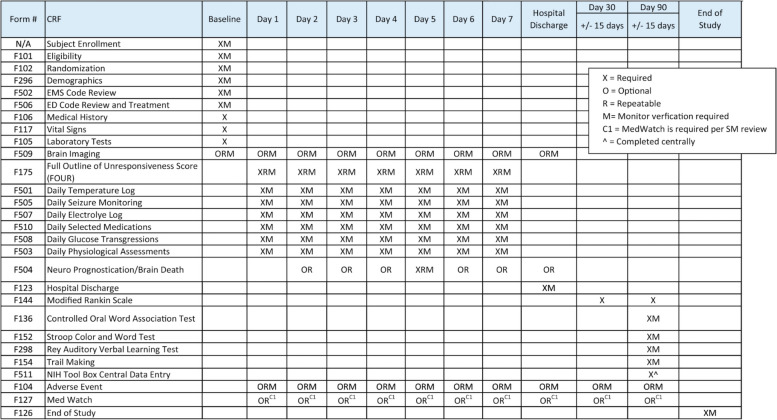
Timeline for data collection

Figure 4 outlines detailed data collection requirements. Study participants will be evaluated 90 days following randomization. Study team members responsible for neuropsychological outcome assessment will be trained and certified per study procedures.

### Plans to promote participant retention and complete follow-up {18b}

Given a patient population of critically ill subjects, initial data collection is the responsibility of the participating facility. Longer-term outcomes will be encouraged by direct contact with the patient or their caretaker with reminders about appointments being implemented.

### Data management {19}

Data management will be handled by the DCC, which is housed in the Data Coordination Unit, of the Department of Public Health Sciences, College of Medicine, Medical University of South Carolina (MUSC), using the WebDCU™ clinical trials management system. This user-friendly web-based system, developed by the DCC, will be used for subject randomization, data entry, data validation, project progress monitoring, subject tracking, tracking, user customizable report generation, and secure data transfer. All data entry activities will be conducted in coordination with the study PIs, the sites, and the CCC. Data validation procedure will be implemented in two stages. First, the automated data checks will flag items that fail a rule, and the rule violation message will appear on the data entry screen at the time of entry. The Study Coordinator at a site will see these rule violations and will be requested to address it. His/her choices are to (1) correct the entry immediately; (2) correct the entry at a later time; or (3) if the entered data are confirmed to be correct, dismiss the rule by checking that option provided by the WebDCU™ system. Any changes made to the data will have a full audit trail. Secondly, for some checks that are more complicated, additional consistency checks will be run periodically after data entry occurs at the site. All data items that fail the programmed consistency checks will be queried via the data clarification request (DCR) process initiated by the DCC data managers.

In addition to the study database, the DCC will provide the site staff password-protected access to a standard set of web-enabled tools, including subject visit calendar, subject accrual status, case report form completion status, and outstanding DCR status pertaining to their respective sites.

The entire study will be conducted using an electronic data acquisition method where all clinical data on enrolled subjects will be data entered (single-keyed) by the site personnel into WebDCU™. In order to provide user-friendly and easy-to-navigate interfaces, the WebDCU™ data capture screens are designed based upon individual Case Report Forms (CRFs). Prior to study start, the system is validated to ensure the data entry screens mirror the CRFs and that the pre-programmed data rules appropriately detect incorrect data. The data will be managed after data entry via data queries from the DCC.

The latest version of each CRF will be available as a PDF file for use as worksheets and source documents by study personnel. This process facilitates version control of these study-related documents, particularly since documents may evolve over the course of the study.

### Confidentiality {27}

The DCU employs several layers of data protection to ensure data security. The first part of security is physical protection of the hardware systems employed by the DCU. The facility housing the DCU hardware is protected 24/7 by multiple layers of security, including electronic building and facility access secured by magnetic locks, onsite-personnel, monitored and recorded closed-circuit television, person-traps, and mandatory identity logging of all outside visitors. By limiting access, ensuring only authorized personnel have access, and tracking all entry, we can ensure this risk is minimal.

The network and system security is ensured by implementing multiple layered firewalls and a network intrusion prevention system for identifying and blocking malicious network activity in real time. Vulnerability scans are also run daily to ensure server and network hardening preventing known application and OS vulnerabilities. Antiviral, Trojan, and worm protection is achieved by using Microsoft Forefront, updated on a daily basis. All communication with the web server and client is encrypted via SSL (secure socket layer) to make certain network traffic “sniffing” poses no threat.

#### Audit trail function for WebDCU™

To maintain electronic records in the database as adequate and accurate, WebDCU™ system tracks all changes made to any study patient-related and dynamically managed electronic records. This audit-trail information is created with a computer generated time-stamp and the user name in chronological order, when the original data is modified or deleted.

#### Data redundancy

The Volume Shadow Copy Service is enabled for all DCU file servers and web servers used in the storage of clinical trial-related documents and website files in order to provide a quick recovery solution of lost data. This allows for “point-in-time” copies of all edited files to be maintained in a hidden file space on the server. The copies or “snapshots” of edited files are taken 3 times daily.

### Backup (disaster recovery)

The databases housed in the WebDCU™ are backed up in two steps. The Microsoft® SQL server maintenance plans are set up to initiate the internal data integrity check-up procedures and to produce off-line backup copies of the database prior to IBM® Tivoli Storage Manager (TSM) backup. The TSM then delivers the full data backup to all DCU servers used in the storage of database at daily basis. The TSM completely backs up all system files (i.e., system registry, operating system, software) and user data files on the server. In the event of a weather-related emergency or other situations where the university implements emergency procedures, the DCU also begins emergency full backup of all servers and other procedures in accordance with the DCU’s Emergency Operation SOP (standard operating procedures).

### Plans for collection, laboratory evaluation, and storage of biological specimens for genetic or molecular analysis in this trial/future use {33}

The ICECAP main trial has no plans for collection and/or storage of biological specimens.

## Statistical methods

### Statistical methods for primary and secondary outcomes {20a}

Formal statistical methods were codified prior to the start of the trial and can be found in the protocol and appendices. This trial will enroll a maximum of 1800 patients. The primary endpoint is a weighted modified Rankin Scale Score (mRS) measured at 90 days after the return of spontaneous circulation. The design of this trial is based on a statistical model of the mean weighted 90-day mRS, i.e., the duration response curve. This trial will enroll patients with and without initially shockable rhythms. All subjects will have already been rapidly cooled at the time of enrollment as a condition of inclusion and will then be randomized to one of ten possible treatment arms for the duration of cooling. The ten possible treatment arms are 6, 12, 18, 24, 30, 36, 42, 48, 60, or 72 h of cooling.

Within each of the two rhythm type populations, patients will be adaptively randomized to a cooling duration. The trial will determine in each of two populations the shortest durations of cooling that provide the maximal treatment effect and whether increasing durations of cooling are associated with better neurological outcomes. In the absence of a normothermia control arm, an increasing treatment effect across some set of durations would imply efficacy of cooling versus no cooling. In this section, we provide an overview of the statistical design and operating characteristics.

The primary endpoint is the 90-day mRS. The primary analysis weights the 7 possible 90-day mRS values. Let M90 be the 90-day mRS. The weight for each possible mRS value is

$$W\left({M}_{90}\right)=\left\{\begin{array}{cc}10& {M}_{90}=0\\ \begin{array}{c}9\\ 8\\ \begin{array}{c}6\\ 0\end{array}\end{array}& \begin{array}{c}{M}_{90}=1\\ {M}_{90}=2\\ \begin{array}{c}{M}_{90}=3\\ {M}_{90}=\text{4,5},6\end{array}\end{array}\end{array}\right.$$

For each treatment arm, we model the mean weighted outcome. The primary analysis of the trial will model the mean weighted mRS for each treatment arm. The primary analysis is conducted on the intent to treat (ITT) population and is conducted separately for each rhythm type. The primary analysis will answer two questions. We will identify the most likely target duration, where the target duration is the shortest duration that achieves the maximum treatment effect (Objective A). We will also determine whether the efficacy of any duration is superior to any shorter duration of cooling (Objective B).

- Objective A: The most likely target duration for rhythm type *r* is h*, where h* is the treatment arm for which the posterior probability that *h* is the target duration is maximized.
- Objective B: The conclusion that cooling duration h* is effective in rhythm type *r* is made if the posterior probability that the mean weighted 90-day mRS for arm h* is greater than the mean weighted 90-day mRS for a duration shorter than h*, is greater than 0.975.

We model the mean weighted 90-day mRS across the ten treatment arms with a duration-response model. All conclusions about each treatment arm will be based on a duration-response model. The duration response model restricts the shape of the duration response curve to have 3 phases—an increasing phase, a plateau phase, and a decreasing phase. We create a parametric family for this inverted-U duration response model. For each rhythm type, a separate and identical instance of the model is used; therefore, we present the details for a single instance. Let θ represent the mean weighted mRS and h represents the treatment arm.

The duration-response model is:

$${\theta }_{h}=\left\{\begin{array}{ll} {\beta }_{0}+{\beta }_{1}{h}^{{\beta }_{3}} & h\le {\gamma }_{1}\\ {\beta }_{0}+{\beta }_{1}{\gamma }_{1}^{{\beta }_{3}} & {\gamma }_{1} < h\le {\gamma }_{2}\\ {\beta }_{0}+{\beta }_{1}{\gamma }_{1}^{{\beta }_{3}}-{\beta }_{2}{\left(h-{\gamma }_{2}\right)}^{{\beta }_{4}}& {\gamma }_{2} < h\end{array}\right.$$

We refer to the parameters *γ*_*1*_ and *γ*_*2*_ as the change-points. The parameter *γ*_*1*_ represents the change point between the increasing phase and the plateau phase. The duration response curve is “flat” between *γ*_*1*_ and the second change point *γ*_*2*_. *γ*_*2*_ represents the change point between this plateau phase and the decreasing phase, so the duration response curve is then decreasing after *γ*_*2*_. An important aspect of the model is that the change-points can be smaller than the minimum cooling duration, *h* = 1 (6 h), or greater than the maximum cooling duration, *h* = 10 (72 h), thus allowing the curve to be increasing, decreasing, or flat over the entire range of cooling. The model has the following constraints: *γ*_*1*_ < *γ*_*2*_ and *β*_*1,*_* β*_*2,*_* β*_*3,*_* β*_*4*_ > *0.*

The *γ*_*1*_ parameter is interpreted as the theoretical optimal duration of cooling, the shortest duration that achieves the maximum treatment effect. We define the *target duration* based on *γ*_*1*_ and* γ*_*2.*_ The target duration is the shortest duration greater than *γ*_*1*_, if *γ*_*1*_ is less than 72 h, or the longest duration if *γ*_*1*_ is greater than 72 h.

At each interim analysis, there will be subjects who have not yet reached 90 days and will therefore not have a final mRS outcome. We use the 30-day mRS value as possibly predictive of the 90-day mRS, allowing subjects with this earlier measurement to be included in the analyses of the 90-day measurement. This modeling is referred to as the longitudinal model. The longitudinal model allows for learning the relationship between the 30-day and 90-day mRS values as the accruing empirical data is used to determine the strength of the association between the two values for each treatment arm and rhythm type. Analyses of the 90-day mRS values are performed with multiple imputation from the longitudinal model for patients with an unknown 90-day mRS value.

The longitudinal model maps the 7 possible 30-day mRS values to the 7 possible 90-day mRS values. We use a Markovian structure for the “transitions” from the 30-day mRS state to the 90-day mRS state. The probability vectors have separate posterior distributions by treatment arm and rhythm type. The observed transitions for the same treatment arm *h* and rhythm type *r* contribute fully to that particular posterior distribution, while the transitions from other treatment arms and for other rhythm types contribute 1/4 of their full weight to the posterior distribution. Thus, there is borrowing of partial information from other treatment durations and the alternate rhythm type.

The first 200 patients will be equally randomized to the 12-, 24-, and 48-h arms. After this initial randomization period, adaptive randomization will begin. During the response adaptive randomization stage, separate allocation schemes are created for each rhythm type. Randomization probabilities to each treatment arm are weighted according to the posterior probability that each treatment arm is the target duration and randomization probabilities will be updated about monthly. The goal of the adaptive randomization is to allocate subjects to the arms most likely to be the target duration, but also to learn effectively about the duration-response curve.

### Interim analyses {21b}

Interim analyses begin after 200 patients have been enrolled and will occur after every 50 patients, or about monthly. At each interim analysis, the trial may stop for futility if no cooling duration greater than 6 h is found to be more effective than the 6 h duration. Futility will be assessed separately for each rhythm type. Therefore, the trial could be declared futile for one rhythm type, and yet continue to enroll subjects of the opposite rhythm type. If both rhythm types are not stopped for futility, the trial will continue to enroll to the maximum sample size of 1800 patients. Specifically, a rhythm type will stop for futility if

1. At least 50 patients have been randomized to the 6-h duration arm for that rhythm
2. There is at least a 50% probability that the 6-h duration is the target duration.

### Methods for additional analyses (e.g. subgroup analyses) {20b}

Further details of the pre-planned secondary analyses will be available in the full statistical analysis plan. Analyses for important subgroups (gender, age strata, pre-existing comorbidities including diabetes, malignancy, prior neurological disease) will be conducted within each rhythm stratum for the primary endpoint and secondary endpoints identified in the statistical analysis plan.

### Methods in analysis to handle protocol non-adherence and any statistical methods to handle missing data {20c}

The primary analyses will be based on the intent-to-treat (ITT) population. The ITT patient population will include all patients randomized, where patients will be included in the treatment arm to which they were randomized, regardless of the duration of cooling applied. Operational procedures are optimized to minimize losing subjects to follow-up and to prevent missingness of data. Previous experience in the network has demonstrated very low rates of missing data. Any subjects that are missing or withdraw from the study and have an unknown 90-day mRS will be included in the analyses of the primary endpoint with multiple imputation according to the longitudinal model previously described.

### Plans to give access to the full protocol, participant-level data, and statistical code {31c}

The full protocol is available on the study website. The study follows NIH policy for patient data repository use. Final statistical code will be available upon request.

## Oversight and monitoring

### Composition of the coordinating center and trial steering committee {5d}

Overall study organization including reporting relationships are per the established structures and standard operating procedures of the SIREN.

The SIREN Clinical Coordinating Center at the University of Michigan will provide overall project management for the trial. Participating sites will be involved through an amendment to the ongoing master agreement between the SIREN CCC and SIREN Hubs. Hubs are responsible for subcontracting with and organizing clinical spoke sites. The SIREN Data Coordinating Center will provide all data management and analytic functions under their own bundled award.

Daily management of the trial will be facilitated by weekly meetings of an operations working group and as a standing scheduled agenda item in weekly meetings of the SIREN operations committee. Strategic decision-making will take place in an executive committee incorporating all participants in the trial leadership.

The ICECAP clinical standardization team will work to refine and train clinical personnel in the consensus standard treatment strategies and will review transgression data.

The SIREN human subject protection working group will review and advise on the informed consent processes in this potentially vulnerable population.

A publications committee will coordinate and support communications about the trial in the published medical literature.

An ICECAP ancillary trial working group will solicit, coordinate, and develop protocols and applications as appropriate to address additional meritorious aims within the framework of the overall trial. Any proposed ancillary studies cannot interfere with the scientific purpose or successful completion of the parent trial. Proposed ancillary studies must be approved by the trial and SIREN leadership, the DSMB, and the NIH.

### Composition of the data monitoring committee, its role and reporting structure {21a}

The NINDS/NHLBI has appointed an independent Data and Safety Monitoring Board (DSMB) for trials conducted in the SIREN network, including the adult ICECAP trial. This DSMB has the responsibility of assuring the safety of trial participants, as well as the continued relevance of the research question, integrity of the data, and appropriateness of the treatment protocol for the ICECAP trial. The DSMB follows the guidelines described in the NIH issued policy on data and safety monitoring. Additionally, the DSMB meets at least once per year to review safety reports provided by the DCC. These reports include details on patient enrollment, baseline characteristics, adverse events, losses to follow-up, and data quality. At each DSMB meeting, the investigators present data with respect to mortality and occurrence of SAEs. The DSMB will monitor and compare rates of adverse events to identify any unexpected trends. After the first 200 subjects have been randomized, additional treatment arms will be opened and patients will be allocated, within each rhythm type, by response adaptive randomization. The implementation of these does not require DSMB approval; however, if requested, the DSMB will be notified of each interim analysis and the corresponding update. The DSMB may recommend stopping the trial at any time, based upon data from the semi-annual reports, the interim analysis report, or external data. The decision to recommend continuing or terminating the study for safety is vested in the DSMB.

### Adverse event reporting and harms {22}

Monitoring of safety is critically important, and among the most central responsibilities of the investigator. The definitions of adverse events (AEs) and serious adverse events (SAEs), expectedness, severity classification, and determination of relatedness are detailed in the extensive Safety Monitoring Plan in the Manual of Procedures.

All AEs occurring through the fourth study day and all serious adverse events (SAEs) occurring until participation in study has ended are recorded on the electronic AE CRF through the WebDCU™. The Hub PI or Study Coordinator or designee is responsible for entering any and all AEs and SAEs into the database as soon as he/she becomes aware of the event and updating the information (e.g., date of resolution, action taken) in a timely manner. Non-serious AEs are collected through the fourth study day. All non-serious AEs occurring through the fourth study day must be recorded on the electronic AE CRF within 5 days from the time it was discovered by the site study personnel. For SAEs, the data entry should occur within a timely manner after the discovery of the event.

The site PI is responsible for the monitoring and follow-up of AEs until resolution (or end of study for that subject) and appropriate documentation in the subject research record. In addition to performing protocol-specified follow-up, the participating PI must review all previously reported ongoing AEs to evaluate the current status. Upon completion of the study protocol by the subject, premature withdrawal from the study by the subject, or subject’s death, all information regarding each AE must be completed, if not done so earlier.

All serious adverse events (SAEs) occurring during a subject’s study participation will be recorded. Additionally, all current study data for that particular subject must be entered to allow for timely review by the medical safety monitors (MSMs). Medical safety monitoring will be conducted as detailed in the ICECAP manual of procedures (MoP). The Project Manager forwards all SAE to an internal quality reviewer, and then an independent MSM, within WebDCU™.

### Frequency and plans for auditing trial conduct {23}

The ICECAP site monitoring plan facilitates compliance with good clinical practice (GCP) guidelines, applicable FDA regulations (21 CFR 812 and 813), and the FDA’s “Guidance for Industry. Oversight of Clinical Investigations—A Risk-Based Approach to Monitoring”. ICECAP site monitoring will be managed by the SIREN CCC at the University of Michigan. The ICECAP Site Monitoring Plan will be updated regularly.

The on-site Monitor will verify data entered into WebDCU™ against source documents. Source documents are original documents, data, and records. Examples include hospital records, clinical and office charts, laboratory notes, evaluation checklists, recorded data from automated instruments, X-rays, study worksheets, and eCRFs (in the case of direct data entry). Monitors will query inaccuracies between the source documents and WebDCU™ database, including the omission of data, and will verify the informed consent of all study participants.

Source document verification may also be performed remotely by reviewing source documents that have been uploaded into WebDCU™ or via remote access to electronic medical records (EMR).

### Plans for communicating important protocol amendments to relevant parties (e.g., trial participants, ethical committees) {25}

As needed, the research network will conduct calls with all sites and submit protocol amendments to the central IRB of record with the FDA.

Any protocol changes impacting the clinical team will require re-training. The training can be planned as face-to-face training, but may also include teleconferences, review of video recording, or other options as deemed appropriate by the Study PI based on the nature of the protocol change. The goal of the training is to ensure that all clinical site personnel who are able to attend receive the same information and are trained the same way in study procedure changes, and with regard to data collection, to try to standardize the methods of data collection to help ensure comparability of data across sites.

### Dissemination plans {31a}

The ICECAP investigators and the SIREN Network are committed to active dissemination of study results and source data.

The primary results of the clinical trial will be disseminated by publication in the peer-reviewed medical literature. In accordance with the NIH Public Access Policy, the investigators will submit an electronic version of their final, peer-reviewed manuscripts (directly or through the publisher) to the National Library of Medicine’s PubMed Central, no later than 12 months after the official date of publication.

ICECAP will be registered and reported in clinicaltrials.gov on or ahead of all scheduled requirements.

The clinical trial will be registered and all required information submitted to clinicaltrials.gov within 6 months of notice of grant award and prior to subject enrollment. Results of the trial will be reported there within a year of trial completion. All submissions to clinicaltrials.gov will be performed consistent with the requirements for applicable clinical trials per FDAAA 801 requirements and NIH policy.

The final ICECAP informed consent document will include a specific statement informing participants that information about the clinical trial is posted at clinicaltrials.gov and that aggregate results will be posted there as well.

After completion of the study and dissemination of primary study results, a public use dataset will be made available through the NHLBI data repository managed by BioLINCC (https://biolincc.nhlbi.nih.gov/home/) or elsewhere as arranged with the Institute. The dataset will be prepared in accordance with the NHLBI Policy for Data Sharing from Clinical Trials and Epidemiological Studies, and in accordance with the Guidelines for NHLBI Data Set Preparation. All manuscripts, abstracts, and press releases using the study data must acknowledge ICECAP/SIREN investigators and the NHLBI as the study sponsor with the relevant grant numbers.

## Discussion

This multi-center, randomized, adaptive allocation clinical trial has the primary objectives to determine the shortest duration of cooling of adult comatose cardiac arrest survivors to provide the maximum treatment effect as well as to determine if increasing the duration of cooling leads to better outcomes.

ICECAP builds upon decades of robust preclinical research, all suggesting a strong neuroprotective effect of hypothermia, as well as multiple clinical studies. This trial is meant to fill in the main gaps in knowledge that remain after interpreting the major moderate to large RCTs in this space. HACA demonstrated statistically significant improvement in outcomes between 33 °C versus no temperature control (24 h) [15]. Similarly, the 2002 study by Bernard et al. showed benefits of hypothermia at 33 °C as compared to normothermia (12 h) [16]. TTM showed similar rates of good neurologic recovery between 33 and 36 °C, leaving some ambiguity about the true effects of hypothermia (36 h for their intervention period) [17]. Most recently the HYPERION trial included patients with non-shockable rhythms and showed significant improvement in favorable neurologic outcomes for hypothermia at 33 °C (24 h) [18]. TTM48 was a duration (dose) finding study and compared 33 °C for 24 to 48 h and suggests better outcomes with longer duration of cooling (48 h) [22]. TTM2 suggests no hypothermia [19].

This prior body of knowledge leaves some questions unanswered, in particular which duration of cooling might offer the most benefit to cardiac arrest patients. The study design of ICECAP is well positioned to provide answers to this question. Additionally, the adaptive design also allows for a shorter duration of cooling and may provide answers about the overall efficacy of cooling, if no benefit for longer durations can be established.

## Potential challenges and limitations

Given the nature and design of this study, some limitations should be considered. As a multi-center study, standardizing clinical practices is difficult. For instance, we allow differences in cooling techniques (endovascular or surface cooling system), similarly we expect differences in how quickly cooling will be achieved.

Based on prior studies, a targeted temperature of 33 °C has been selected. While this is standard clinical practice in many locations due to these prior results, the maximally beneficial duration of hypothermia may be different based on the targeted temperature.

As patient enrollment started during the COVID-19 pandemic, there may be changes to local EMS protocols or patients presenting with pathologies that do not fully represent out of hospital cardiac arrest in non-pandemic times.

## Trial status

Recruitment started on May 18, 2020. As of February 2024, enrollment included approximately 875 participants over roughly 64 sites. We estimate a completion date for enrollment within 5 years. The currently approved protocol version is 1.

### Supplementary Information

Supplementary Material 1.

Supplementary Material 2.

Supplementary Material 3.

## Data Availability

Study investigators will have access to the final trial dataset without contractual agreements that limit access. As per the U.S. public access requirement, public use datasets will be made available in de-identified form on a repository.

## Abbreviations

AE: Adverse event
CCC: Clinical Coordinating Center
CIRB: Central Institutional Review Board
CRF: Case Report Form
DCC: Data Coordinating Center
DCR: Data Clarification Request
DCU: Data Coordination Unit
DSMB: Data and Safety Monitoring Board
ED: Emergency department
EMS: Emergency Medical Services
FDA: Food and Drug Administration
GCP: Good Clinical Practice
HRQOL: Health-Related Quality Of Life
ICU: Intensive care unit
ITT: Intention to treat
IRB: Institutional Review Board
IV: Intravenous
LAR: Legally authorized representative
MoP: Manual of Procedures
mRS: Modified Rankin Scale
MSM: Medical Safety Monitor
MUSC: Medical University of South Carolina
NIH: National Institutes of Health
OHCA: Out of Hospital Cardiac Arrest
SAE: Serious adverse event
SIREN: Strategies to Innovate Emergency Care Clinical Trials Network
SSL: Secure Socket Layer
SOP: Standard operating procedures
