# Supplementary material for: Influence of Cooling duration on Efficacy in Cardiac Arrest Patients (ICECAP): study protocol for a multicenter, randomized, adaptive allocation clinical trial to identify the optimal duration of induced hypothermia for neuroprotection in comatose, adult survivors of after out-of-hospital cardiac arrest
Source: Trials. 2024 Jul 23;25:502. doi: 10.1186/s13063-024-08280-w (PMC11264458; doi:10.1186/s13063-024-08280-w)
Supplement: Supplementary file 3 — Supplementary Material 3. [file 13063_2024_8280_MOESM3_ESM.pdf]

Bayesian Adaptive Trial Design for the Influence of Cooling duration on Efficacy in Cardiac Arrest Patients (ICECAP)

Berry Consultants, LLC
Last Updated April 22, 2020

1.0 Background

This is a multicenter, randomized adaptive clinical trial in comatose adult survivors of out of hospital cardiac arrest that have already been rapidly cooled. A maximum of 1800 patients will be enrolled. The primary endpoint is a weighted modified Rankin Score (mRS) measured at 90 days after the return of spontaneous circulation. This trial will enroll patients with and without initial shockable rhythms. Within each rhythm type, patients will be adaptively randomized to a cooling duration. The primary objective of this trial is to characterize, for each rhythm type, the duration-response curve for cooling and to identify the duration that provides the maximum treatment effect, referred to as the *target* duration. This document describes the complete details of the adaptive trial design, including statistical model, design parameters, and the operating characteristics.

1.1 Treatment Arms

All subjects will have already been rapidly cooled (33 °C +/– 0.5°C) and will then be randomized to one of ten treatment arms for the duration of cooling. The ten treatment arms are 6, 12, 18, 24, 30, 36, 42, 48, 60, or 72-hours of cooling. For statistical modeling we label these arms as h=1,2,…,10.

1.2 Primary Endpoint

The primary endpoint is the 90-day modified Rankin score (mRS). The mRS is an integer-valued outcome from 0 to 6, with smaller values indicating a better neurological status. A summary of each of the outcomes is:

0 = No symptoms
1 = No significant disability; able to carry out all usual activities
2 = Slight disability; can look after own affairs, but unable to carry out all previous activities
3 = Moderate disability; require some help
4 = Moderately severe disability; unable to attend to own bodily needs without assistance
5 = Severe disability; bedridden and requiring constant nursing care and attention
6 = Dead

1.3 Primary Analysis

The primary endpoint is the 90-day mRS. The primary analysis weights the 7 possible 90-day mRS values. Let M_{90} be the 90-day mRS. The weight for each possible mRS value, M_{90}, is

$$W(M_{90}) = \begin{cases} 10 & M_{90} = 0 \\ 9 & M_{90} = 1 \\ 8 & M_{90} = 2 \\ 6 & M_{90} = 3 \\ 0 & M_{90} = 4,5,6 \end{cases}$$

For each treatment arm the mean weighted outcome is modeled. For any treatment arm, let $p_0 ... p_6$ be the probability of each outcome. The mean weight for the treatment arm is

$$\sum_{m=0}^{6} p_m W(m)$$

The primary analysis of the trial will model the mean weighted mRS for each treatment arm. Let $\theta_{h,r}$ be the mean weighted 90-day mRS for treatment arm *h* within rhythm type *r*. The rhythm types are labeled as *r* = 1 (shockable) and *r* = 2 (not shockable).

The primary analysis is conducted separately for each rhythm type and has two components. First, we identify the most likely target duration, where the target duration is the shortest duration that achieves the maximum treatment effect (target duration is further described in Section 2.3.1). Second, to determine if cooling is effective in increasing the rate of good neurological outcomes, in the absence of a control arm, we calculate whether the most likely target duration is superior to the shorter durations of cooling. If so, this indicates cooling is effective in that at least one part of the duration-response curve is increasing.

1. The treatment arm that is the most likely target duration for rhythm type *r* is *h** such that the posterior probability *h* is the target duration is maximized (see Section 2 for complete details).

2. The conclusion that cooling duration *h** is effective in rhythm type *r* is made if the posterior probability that the mean weighted 90-day mRS for arm *h** is greater than the mean weighted 90-day mRS for a duration shorter than *h**, is greater than 0.975. That is, for some *h* < *h**,

$$\Pr\left(\theta_{h^*,r} > \theta_{h,r} \mid \text{data}\right) > 0.975$$

The statistical modeling is further described in Section 2.

1.4 Analysis Population

All analyses will be based on the intent-to-treat ITT population. The ITT patient population will include all patients randomized, where patients will be included in the treatment arm to which they were randomized, regardless of the duration of cooling applied.

It is anticipated that subjects may initially be perceived to be of one rhythm type and later found to be of the other rhythm type. In the ITT analyses, these subjects will be included with their corrected rhythm type, but their treatment arm will remain as randomized.

Any subjects that are missing or withdraw from the study and have an unknown 90-day mRS will be included in the analyses of the primary endpoint with multiple imputation according to the longitudinal model described in Section 2.2.

1.5 Randomization

The first 200 subjects enrolled overall between the two rhythm types will be randomized equally (1:1:1) between three treatment arms: 12 hours, 24 hours, and 48 hours. After these first 200 patients have been enrolled, patients will be allocated to treatment arms (durations 12 to 48) based on response adaptive randomization (RAR). RAR will be applied separately within each rhythm type. During RAR, interim analyses will be conducted approximately every 50 enrollments in order to update the response adaptive randomization probabilities. The response adaptive randomization probabilities determined at each interim analysis will be applied for the newly enrolling subjects until the next interim analysis and update. Section 2 describes the calculation of the response adaptive randomization probabilities.

The 6, 60, and 72-hour duration treatment arms are initially closed to enrollment. The 6-hour duration treatment arm will be opened for a rhythm type *r* if there are more than100 subjects enrolled across all arms in that rhythm type and there is at least a 0.33 probability that 6 hours is the target duration for that rhythm type. The 60 and 72-hour duration treatment arms will be opened incrementally. These arms open on either rhythm if there is at least a 0.33 probability that the target duration for that rhythm type is at or above that next shorter duration.

1.6 Stopping Rules

Interim analyses begin after 200 patients have been enrolled and will occur about every 50 enrollments. At each interim analysis, the trial may stop for futility if no cooling duration greater than 6 hours is found to be more effective than the 6-hour duration. Futility will be assessed separately for each rhythm type. Therefore, the trial could be declared futile for one rhythm type, and yet continue to enroll subjects of the opposite rhythm type. If both rhythm types are not stopped for futility, the trial will continue to enroll to the maximum sample size of 1800 patients. Specifically, a rhythm type will stop for futility if

1. At least 50 patients have been randomized to the 6-hour duration arm for that rhythm
2. There is at least a 50% probability that the 6-hour duration treatment arm is the target duration

2.0 Statistical Modeling

This section describes the statistical models used in the adaptive design and the primary analysis. The modeling is Bayesian in nature. All conclusions about each treatment arm will be based on a duration-response model. We use an inverted U-shaped duration-response model for modeling the mean weighted 90-day mRS for each treatment arm. Identical instances of the model are used for each rhythm type. During the trial, at the interim analyses, patients with unknown 90-day mRS values are modeled using a longitudinal model based on earlier, 30-day mRS, values. This same longitudinal model is used to handle missing data for the primary analysis at the conclusion of the trial.

The inverted U-shaped duration-response model is described in Section 2.1, the longitudinal model is described in Section 2.2, the Bayesian quantities calculated at each interim analysis are described in Section 2.3, and the calculation of the response adaptive randomization probabilities is described in Section 2.4.

Let the observed weight for a patient *i* be W_i. The mean weighted 90-day mRS value for an individual duration, $\overline{W}_{h,r}$, are modeled as normally distributed:

$$[\overline{W}_{h,r}] \sim N(\theta_{h,r}, \frac{\sigma_r^2}{n_{h,r}})$$

where the treatment arm is *h* and the rhythm type is *r*. The patient-level utility responses are bounded between 0 and 10, and likely non-normally distributed, but the mean response at a duration, within a rhythm type will be almost exactly normally distributed for sample sizes ≥ 10. The variance components are modeled separately for each rhythm type, with weak prior distributions:

$$[\sigma_r^2] \sim \mathcal{IG}(2.5, 22.5) \text{ for } r = 1, 2$$

2.1 Duration Response Model for Weighted mRS

We model the mean weighted 90-day mRS across the ten treatment arms with a duration-response model. The duration response model is a critical aspect to the adaptive design and the primary analysis. The duration response model restricts the shape of the duration response curve to have 3 phases – an increasing phase, a plateau phase, and a

decreasing phase. We create a parametric family for this inverted-U duration response model. For each rhythm type a separate and identical instance of the model is used, therefore we present the details for a single instance.

The duration-response model is:

$$\theta_h = \begin{cases} \beta_0 + \beta_1 h^{\beta_3} & h \le \gamma_1 \\ \beta_0 + \beta_1 \gamma_1^{\beta_3} & \gamma_1 < h \le \gamma_2 \\ \beta_0 + \beta_1 \gamma_1^{\beta_3} - \beta_2 (h - \gamma_2)^{\beta_4} & \gamma_2 < h \end{cases}.$$

In addition, the values of θ_h will be restricted to be between 0 and 10. We refer to the parameters γ_1 and γ_2 as the change-points. The parameter γ_1 represents the change point between the increasing phase and the plateau phase. The duration response curve is "flat" between γ_1 and the second change point, γ_2. γ_2 represents the change point between this plateau phase and the decreasing phase, so the duration response curve is then decreasing after γ_2. An important aspect of the model is that the change-points can be smaller than the minimum cooling duration, *h*=1 (6 hours), or greater than the maximum cooling duration, *h*=10 (72-hours), thus allowing the curve to be increasing, decreasing, or flat over the entire range of cooling. The model has the following constraints: $\gamma_1 < \gamma_2$ and $\beta_1, \beta_2, \beta_3, \beta_4 > 0$. Figure 1 shows an example duration-response curve with parameters $\gamma_1=4.33$, $\gamma_2=6.33$, $\beta_0=3$, $\beta_1=0.32$, $\beta_2=0.30$, $\beta_3=1.2$, and $\beta_4=1$.

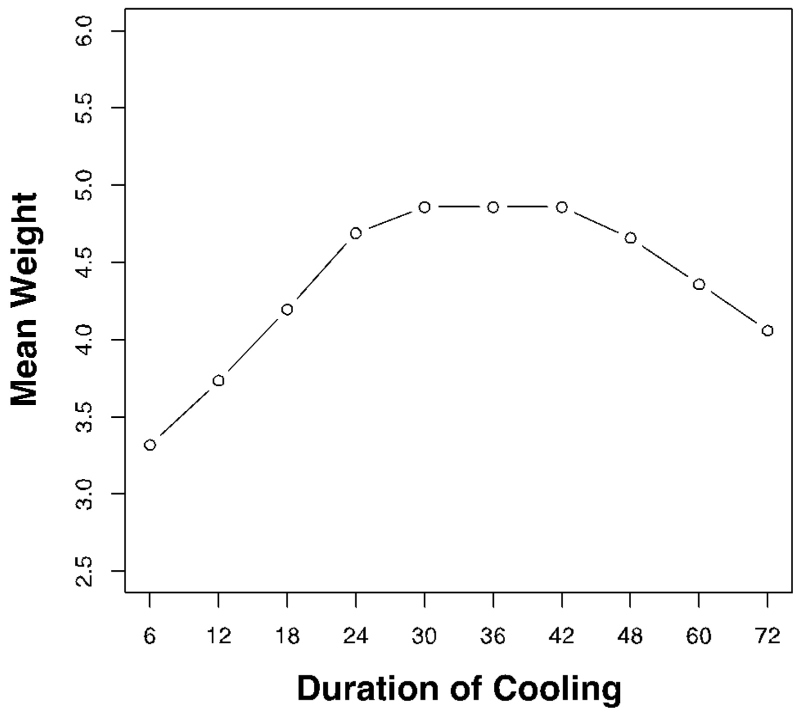

Figure 1: An example duration response curve

The γ_1 parameter is interpreted as the theoretical optimal duration of cooling, the shortest duration that achieves the maximum treatment effect. We define the *target duration* based on γ_1 and γ_2. The target duration is the shortest duration greater than γ_1, if γ_1 is less than 72-hours, or the longest duration if γ_1 is greater than 72-hours.

Because the modeling is done separately for each rhythm type, we add a subscript *r* on the parameters to notate the specific rhythm type. The prior distributions for the "change-points" are defined on the space where $\gamma_1^r < \gamma_2^r$ as follows:

$$\left[\gamma_1^r\right] \propto N\left(4, 10^2\right) \text{ for } r = 1,2$$

and

$$\left[\gamma_2^r\right] \propto N\left(8, 3^2\right) \text{ for } r = 1,2.$$

The following priors for each instance are utilized:

$$\left[\beta_0^r\right] \sim N\left(4, 4^2\right) \text{ for } r=1,2$$

$$\left[\beta_j^r\right] \propto 1 \text{ for } j=1,\ldots,4;\ r=1,2,$$

For the final analysis the selection of the most likely optimal duration within each rhythm type is selected using this model described above. For the determination of a positive treatment effect the posterior distribution using a hierarchical model is used. The following hierarchical prior structure is used:

$$\left[\gamma_1^r\right] \propto N\left(\mu_1, \tau^2\right) \quad \text{for } r = 1,2$$

and

$$\left[\gamma_2^r\right] \propto N\left(\mu_2, \tau^2\right) \quad \text{for } r = 1,2.$$

With hyperparameters

$$\left[\boldsymbol{\mu}_1\right] \sim N\left(4, 10^2\right)$$

and

$$\left[\boldsymbol{\mu}_2\right] \sim N\left(8, 3^2\right)$$

and

$$[\tau^2] \sim \mathcal{IG}(0.05, 0.001)$$

.

2.2 Longitudinal Modeling

At each interim analysis, and at the final analysis, there will be subjects who have an unknown 90-day mRS value. We use the 30-day mRS value as possibly predictive of the 90-day mRS, allowing subjects with this earlier measurement to be included in the analyses of the 90-day measurement. This modeling is referred to as the longitudinal model. The longitudinal model allows for learning the relationship between the 30-day and 90-day mRS values as the accruing empirical data is used to determine the strength of the association between the two values for each treatment arm and rhythm type. Analyses of the 90-day mRS values are performed with multiple imputation from the longitudinal model for patients with an unknown 90-day mRS value.

The longitudinal model maps the 7 possible 30-day mRS values to the 7 possible 90-day mRS values. We present a Markovian structure for the "transitions" from the 30-day mRS state (*k*) to the 90-day mRS state (*j*). The transition matrix, *P*, represents the matrix of probabilities for a subject transitioning from the 30-day mRS state (rows) to the 90-day mRS state (columns). For example, p_{32} is the probability a subject that is a 30-day mRS of 3 becomes a 2 at 90-days.

$$P = \begin{bmatrix} p_{00} & p_{01} & & \cdots & & p_{05} & p_{06} \\ p_{10} & p_{11} & & & & p_{15} & p_{16} \\ & & \ddots & & & & \\ \vdots & \vdots & & p_{kj} & & \vdots & \vdots \\ & & & & \ddots & & \\ & & & & & & \\ p_{60} & p_{61} & & & & p_{65} & p_{66} \end{bmatrix}$$

We place a Dirichlet prior distribution on each vector of probabilities from each state at 30 days to each state at 90 days:

$$(p_{k0} \quad \cdots \quad p_{k6})^T \sim Dirichlet((\alpha_{k0} \quad \cdots \quad \alpha_{k6})^T) \text{ for } k = 0, \ldots, 6$$

For all treatment arms and both rhythm types we assume the following prior weights for the transitions (included is the assumption that all transitions from a 30-day mRS of 6 are to a 90-day mRs of 6):

$$\alpha_{kj} = \begin{cases} 0.1, & k \neq 6 \\ 0, & k = 6, j \neq 6 \\ 1, & k = 6, j = 6 \end{cases}$$

Each prior weight can be interpreted as the number of subjects that have made that particular transition. Thus, these prior weights will be quickly overwhelmed by the accruing empirical data and yet provide reasonable estimates if the data are minimal for an mRS state.

The probability vectors have separate posterior distributions by treatment arm and rhythm type. The observed transitions for the same treatment arm *h* and rhythm type *r* contribute fully to that particular posterior distribution, while the transitions from other treatment arms and for other rhythm types contribute 1/4 of their full weight to the posterior distribution. Thus, there is borrowing of partial information from other treatment arms and the opposite rhythm type.

Let the number of subjects in the trial that transition from mRS state *k* at 30-days to mRS state *j* at 90-days, for treatment arm *h* and rhythm type *r*, be

$$T_{h,r}(k,j).$$

We model the posterior distribution for the probability vector from mRS state *k*, for treatment arm *h* and rhythm type *r* as

,

$$Z_{h,r} = \begin{pmatrix} P_{k0} \\ \vdots \\ P_{kj} \\ \vdots \\ P_{k6} \end{pmatrix} = Dir\begin{pmatrix} \alpha_{k0} + T_{h,r}(k,0) + \frac{1}{4} \sum_{h',r' \in (h,r)'} T_{h',r'}(k,0) \\ \vdots \\ \alpha_{kj} + T_{h,r}(k,j) + \frac{1}{4} \sum_{h',r' \in (h,r)'} T_{h',r'}(k,j) \\ \vdots \\ \alpha_{k6} + T_{h,r}(k,6) + \frac{1}{4} \sum_{h',r' \in (h,r)'} T_{h',r'}(k,6) \end{pmatrix}$$

where (*h*,*r*)’ refers to all treatment arms and the rhythm type that are different than the (*h*,*r*) pair.

2.3 Bayesian Quantities

The following Bayesian quantities are calculated for each analysis. Each of these quantities are calculated using the data from all subjects in the trial—those with complete 90-day mRS values and those with only 30-day mRS values.

2.3.1 Most Likely Target Duration

The target duration is defined as the shortest duration that achieves the maximum treatment effect. As described in Section 2.1, the target duration is defined based on the duration-response model as the shortest duration greater than γ_1, if γ_1 is less than 72-hours, or the longest duration if γ_1 is greater than 72-hours.

At each analysis the posterior probability that each treatment arm is the target duration is calculated for each rhythm type. The treatment arm with the highest posterior probability of being the target duration for a particular rhythm type is the most likely target duration for that rhythm type. We label the probability a treatment arm *h* is the target duration for rhythm type *r* as

$$\Pr_{h,r}$$

2.3.2 Posterior Variance

For each treatment arm *h* and rhythm type *r* we calculate $\theta_{h,r}$, the posterior mean weighted 90-day mRS and $V(\theta_{h,r})$, the posterior variance of $\theta_{h,r}$.

2.3.3 Posterior probability superior to smaller duration

Within each rhythm type *r*, and each duration *h*, we calculate the posterior probability that it has a superior mean weighted 90-day mRS as compared to each treatment arm with a shorter cooling duration. Because of the duration-response model, it is sufficient to calculate only the posterior probability that a treatment arm is superior to the 6-hour duration arm. We refer to the posterior probability that each treatment arm is superior to a shorter duration treatment arm as

$$\Pr\left(\theta_{h,r} > \theta_{1,r} \mid \text{data}\right).$$

This quantity is calculated using both independent priors and using the hierarchical priors.

2.4 Adaptive Randomization

During the response adaptive randomization stage, separate allocation schemes are created for each rhythm type. The specification of the vector of probabilities for randomization is defined in this section. Randomization probabilities to each treatment arm are weighted according to the posterior probability that each treatment arm is the target duration. The goal of the adaptive randomization is to allocate subjects to the arms most likely to be the target duration, but also to learn effectively about the duration-response curve.

The randomization probabilities for each treatment arm within each rhythm type are proportional to $\Pr_{h,r}$, the probability that a treatment arm is the most likely target duration for a rhythm type *r.*

$$q_{h,r} = \frac{\Pr_{h,r}\delta_{h,r}}{\sum_{l=1}^{10}\Pr_{l,r}\delta_{l,r}} \text{ for } h = 1,2,\ldots,10$$

The treatment arms for 6-hours (h=1), 60 hours (h=9), and 72 hours (h=10) will initially be closed, but may be later opened according to the rules described in Section 1.5. If a treatment arm is open we set $\delta_{h,r} = 1$, and if a treatment arm is closed we set $\delta_{h,r} = 0$. Therefore, the adaptive allocation scheme favors the arms most likely to be the target duration, but also favors arms with a greater variability (uncertainty) around the primary endpoint or a smaller sample size.

3.0 Section intentionally left blank

Reserved for future use.

4.0 Operating Characteristics

The operating characteristics of the design are calculated using simulation. We consider a variety of scenarios for the true duration-response curve. There are many possible performance measures for this design including the probability of selecting each duration, the probability cooling is considered effective, the mean number of patients allocated to each duration, and the probability of opening the 6, 60, or 72-hour durations. In order to calculate such properties of the design, we create a methodology for simulating virtual patients. This is described in Section 4.6.

Section 4.1 addresses the type I error of the design. We define Type I error as the probability, across all doses, that cooling is considered effective when in truth, the duration response curve is flat.

Sections 4.2, 4.3, and 4.4 address the probability of success, or power of the design when there is a positive duration-response relationship. These sections show the properties of the design for different assumed effect sizes and shapes of the duration-response curves. There are two objectives of this trial and we define the probability of success for each. The first objective is to select a target duration. For each scenario, we define clinically acceptable durations. First there is the optimal duration, which is the shortest duration that achieves the maximum treatment effect. We additionally identify durations that are considered clinically acceptable. These durations must be within 12 hours of the optimal duration and must achieve at least 70% of the maximum treatment effect. We define power for this objective as the probability any one of these clinically acceptable durations is selected as the target duration. The second objective is to determine if there is a positive duration-response curve (i.e. that cooling is effective). We define power for this objective in scenarios where there is positive effect of cooling, as the probability the duration-response curve is found to be positive, regardless of the selected target duration.

4.1 Final Analysis and Type I Error

This section discusses the final primary analysis and the threshold for success. The final primary analysis within each rhythm type is to test whether the duration-response is positive for a range of durations. The parametric structure of the duration-response model is one that forces the effect to be one of the following:

1. Decreasing effect over all durations
2. A plateau effect (same response) for all durations
3. A plateau effect for a range of durations then a negative effect for longer durations.
4. Increasing effect over all durations
5. Increasing effect for a range of durations then becoming a plateau
6. Increasing effect for a range of durations, then a plateau effect for a range of durations, then becoming a negative effect.

Because of these limited relationships, if any of the truths in 4-6 above are the reality this would entail that there is an increasing benefit for cooling for a range of doses. The

primary analysis will calculate the posterior probability that one of these relationships hold. In this modeling scenario this is equivalent to testing whether the most likely target duration is more effective than a smaller duration.

The trial design is Bayesian and hence the primary analysis is a conclusion using the posterior probability that the selected target duration is superior to smaller durations. The final analysis of a 97.5% cut-off for the posterior probability will be used and was selected as an appropriately judged threshold for success. This analysis is appropriate given the multiple interim analyses and unusual nature of investigating a positive duration response model with no control arm, no predefined maximum arm, and the possibility of a non-monotonic duration response.

We simulate different null scenarios to understand the type I error of the design and analysis. The design of this trial does not increase type I error. There is no early stopping for success in the trial, nor is there selection of patient subgroups. The adaptations used in this trial are response adaptive randomization (RAR) and futility stopping. Depending on the circumstances RAR may increase or decrease type I error. Imbalance in covariates can cause type I errors, but RAR will typically decrease type I errors. This is because under the null hypothesis where all arms are equal, an arm that performs better by chance will receive more subjects, allowing a greater ability to diagnose the lack of efficacy. In addition, futility stopping also decreases type I error. The question of type I error control is then whether the method for the primary analysis maintains control of type I error. We present simulations of the type I error rate for this design and analysis to understand the type I error.

Under the null hypothesis, that all durations have the same utility, the simulated type I error may depend on the relative probabilities of the mRS outcomes, the accrual rates, the longitudinal pattern of 30-day mRS to 90-day mRS, and the proportion of subjects in each rhythm type. We present several null scenarios to explore the null space and report the simulated type I error.

The following null cases are simulated. Each case has an assumed accrual rate, longitudinal pattern, proportion of subjects that are shockable, and distribution of mRS values. The medium (med) accrual rate is a maximum of 8.65 subjects per week. The fast accrual rate is 50% faster, reaching a maximum of 12.98 subjects per week, and the slow is 50% slower, reaching a maximum of 4.33 subjects per week. The slow accrual rate is used as the typical scenario, anticipating that slower accrual may inflate the type I error. The "Long" column reports the longitudinal pattern of the 30-day mRS value. The longitudinal pattern 1 is the pattern presented in Section 4.6, while a value of 2 means each value is equally likely to have occurred at 30-days given the 90-day value (except death is still expected in 50% of 90-day deaths). The "Prob Shock" column is the proportion of subjects enrolled with a shockable rhythm. The 7 mRS columns report the probability of each mRS outcome. The probabilities of each mRS outcome for each treatment arm are identical (hence no benefit to extended cooling). The simulation frequency of meeting the primary efficacy outcome, of a posterior probability of at least 97.5% for a positive duration-response model for each

rhythm type, regardless of the target duration selected, are reported in the last two columns. For scenarios 1-6, the two rhythm types are the same, and each with equal effects across all durations, so the average probability of a positive duration response is reported. In Scenarios 7 through 13 the results per rhythm type are important and are listed (shock/non-shock).

Null Case	Accrual	Long	Prob Shock	Probability of Each mRS Value							Probability Positive Duration Response
				0	1	2	3	4	5	6	
1	Slow	1	0.50	.20	.15	.15	.05	.05	.10	.30	0.0354
2	Slow	2	0.50	.20	.15	.15	.05	.05	.10	.30	0.0392
3	Med	1	0.50	.20	.15	.15	.05	.05	.10	.30	0.0454
4	Fast	1	0.50	.20	.15	.15	.05	.05	.10	.30	0.0374
5	Slow	1	0.50	.10	.10	.15	.10	.05	.10	.40	0.0318
6	Slow	1	0.50	.25	.20	.20	.10	.05	.10	.10	0.0444
7	Slow	1	0.55	.20	.15	.15	.05	.05	.10	.30	0.0352/0.0308
8	Slow	1	0.60	.20	.15	.15	.05	.05	.10	.30	0.0464/0.0376
9	Slow	1	0.65	.20	.15	.15	.05	.05	.10	.30	0.0436/0.0408
10	Slow	1	0.70	.20	.15	.15	.05	.05	.10	.30	0.0384/0.0256
11	Slow	1	0.75	.20	.15	.15	.05	.05	.10	.30	0.0392/0.0264
12	Slow	1	0.80	.20	.15	.15	.05	.05	.10	.30	0.0452/0.0248
13	Slow	1	0.85	.20	.15	.15	.05	.05	.10	.30	0.0424/0.0304

Each of the simulations is based upon 2000 simulated trials. The simulated type I error is typically smaller than 0.05 (a one-sided type I error). The different underlying distribution of mRS can have subtle effects on the behavior of the dose-response model. Cases 1 and 5 use the same assumptions, except for the distribution of mRS and have simulated type I errors of 0.0318 or 0.0454. The accrual rate and longitudinal pattern have little effect on the simulated type I error. Scenario 6 has a higher likelihood of good outcomes and has a higher type I error rate, 0.0444. The proportion of patients with shockable rhythms versus non-shockable rhythms does not have a large effect on the simulated type I error, unless the rate of subjects within one rhythm type is very small (< 20%), then the type I error is deflated for the smaller group.

Because of design assumptions regarding the distribution of the mRS, our team has a plan to determine the type I error under the actual observed distribution of mRS observed in the trial. By taking all outcomes in the trial, and then using numerical simulation to pull subjects randomly into arms we are creating an additional null scenario that is based on the actual observed distribution of mRS in the ICECAP trial. By observing how many trials are falsely positive, this will provide additional confirmation that type I error is well controlled.

Additional details for the post-trial numerical simulation plan to confirm type I error are as follows. At the conclusion of the trial, the design will be re-simulated using the actual subject data. Within each rhythm type all subjects will be placed in a list with their arm

assignment removed. Subjects will be randomly sampled or "bootstrapped" from this list and assigned a duration. This creates a setting where, theoretically, all durations have the same treatment effect as all subjects are drawn from the same pool. The post-simulation type I error will be reported as the type I error for the trial. The 97.5% threshold will be used for primary success, regardless.

The post-simulation type I error for the observed posterior probability that cooling is effective within each rhythm type will be reported. So, for example, it may be reported that the post-simulation type I error for an observed threshold of 99.4% within the shockable rhythm type is 0.004. The post-simulation type I error calculation details will be presented for understanding, while the posterior probability of a positive duration-response and the probability of each duration being the target duration will be the primary analysis summaries.

4.2 Power: Duration-response curves are the same in both rhythm types

In these scenarios, the duration response curve is the same in both rhythm types (shockable and non-shockable) and we explore different magnitudes of treatment effects and shapes for the duration-response curve. Each of the scenarios in this section assumes that 50% of patients will be in each rhythm type. Sensitivity to this assumption is presented in Section 4.4.

For each scenario, we present by rhythm type the assumed true mean weighted mRS score for each treatment arm, the probability each arm is selected as the target duration (Pr(Target)), the probability the arm is both selected as the target duration arm and is considered more effective versus a shorter duration (Pr(Target & Eff)), the mean number of patients allocated to each arm, and the total mean sample size. For each scenario, the clinically acceptable durations are shown in red italics. In section 4.6 we show, for each mean weighted mRS in these scenarios, the corresponding unique proportion of patients with each mRS outcome at 90 days. Operating characteristics are based on 2000 simulations of each scenario. They are summarized in the following table and then presented in detail for each scenario.

**Table 4.2 Section Summary**	**Shockable Rhythm**			**Non-Shockable Rhythm**		
	**SAT**	**Pos**	**Fut**	**SAT**	**Pos**	**Fut**
Small Effect at 18 hours	0.643	0.556	0.022	0.637	0.547	0.019
Strong Effect at 30 hours	0.78	0.935	0.003	0.781	0.938	0.003
U-shaped duration response	0.832	0.709	0.013	0.824	0.699	0.018
Increasing duration response	0.39	0.847	0.015	0.396	0.845	0.02
Larger treatment effect at 48 hours	0.777	0.994	0.002	0.763	0.993	0.002
Cooling longer than 6 hours is harmful	0.926	0	0.968	0.922	0	0.966
Treatment effect at 18 hours	0.802	0.778	0.003	0.792	0.774	0.003

SAT = Pr(Select Acceptable Target Duration)
Pos = Pr(Positive Duration Response)
Fut = Pr(Futility)

4.2.1 Small Treatment Effect at 18 Hours (Scenario 21.21)

In this scenario, increasing cooling duration provides a small treatment effect and the duration response curve plateaus at 18 hours. The 6-hour arm is enrolled to with 53% probability, the 60-hour arm is enrolled to with 14% probability, and the 72-hour arm is enrolled to with only 1% probability. The trial continues to completion with 98% probability (i.e. will stop early for futility only 2% of the time).

The 18 hours duration, and the two surrounding arms, 12- and 24-hours have the largest sample sizes. The adaptive randomization tends to place patients on these doses in order to identify where the treatment effect plateau begins.

The durations considered clinically acceptable are the 18-, 24-, and 30-hour durations. They are each within 12 hours of the 18-hour duration and offer the same treatment effect. One of these arms is selected with 64% probability in each rhythm type. In total, across all treatment arms, the positive duration-response curve is identified with 55% probability.

Table 4.2.1A: Summary Scenario Operating Characteristics

	Shockable	Non-Shockable
Pr(Select Acceptable Target Duration)	0.643	0.637
Pr(Positive Duration Response)	0.556	0.547
Pr(Futility)	0.022	0.019
Pr(Open 6 Hours)	0.540	0.523
Pr(Open 60 Hours)	0.138	0.147
Pr(Open 72 Hours)	0.014	0.012

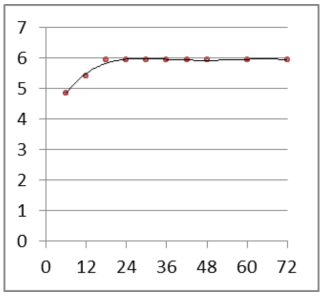

Table 4.2.2B: Detailed Scenario Operating Characteristics

	Shockable Rhythm				Non-Shockable Rhythm			
	Mean Weighted mRS	N	Pr (Target)	Pr (Target & Eff)	Mean Weighted mRS	N	Pr (Target)	Pr (Target & Eff)
6 Hour	4.85	29.6	0.076	0.000	4.85	28.0	0.088	0.000
12 Hour	5.39	156.6	0.168	0.056	5.39	155.6	0.160	0.062
18 Hour	*5.93*	*150.1*	*0.333*	*0.210*	*5.93*	*152.1*	*0.322*	*0.190*
24 Hour	*5.93*	*168.7*	*0.228*	*0.155*	*5.93*	*169.5*	*0.240*	*0.169*
30 Hour	*5.93*	*110.3*	*0.082*	*0.058*	*5.93*	*110.4*	*0.075*	*0.053*
36 Hour	5.93	94.2	0.046	0.032	5.93	94.4	0.047	0.032
42 Hour	5.93	82.0	0.038	0.025	5.93	82.1	0.033	0.021
48 Hour	5.93	100.2	0.024	0.016	5.93	100.5	0.028	0.017
60 Hour	5.93	7.3	0.004	0.004	5.93	7.6	0.006	0.002
72 Hour	5.93	0.5	0.000	0.000	5.93	0.4	0.000	0.000

4.2.2 Strong Treatment Effect at 30 Hours (Scenario 32.32)

In this scenario, increasing cooling duration provides a stronger treatment effect and the duration response curve plateaus at 30 hours. The 6-hour arm is enrolled to with 25% probability, the 60-hour arm is enrolled to with 49% probability, and the 72-hour arm is enrolled to with 10% probability. The trial continues to completion with 99% probability (i.e. will stop early for futility less than 1% of the time).

The 30-hour duration, and the two surrounding arms, 24- and 36-hours have the largest sample sizes. This allocation aids in identifying the treatment effect plateau. The 24-, 30-, 36- and 42-hour durations are considered clinically acceptable. These arms are selected as the target duration with approximately 78% probability in each rhythm type. The positive duration response curve is identified with 94% probability.

Table 4.2.2A: Summary Scenario Operating Characteristics

	Shockable	Non-Shockable
Pr(Select Acceptable Target Duration)	0.780	0.781
Pr(Positive Duration Response)	0.935	0.938
Pr(Futility)	0.003	0.003
Pr(Open 6 Hours)	0.256	0.246
Pr(Open 60 Hours)	0.484	0.490
Pr(Open 72 Hours)	0.101	0.095

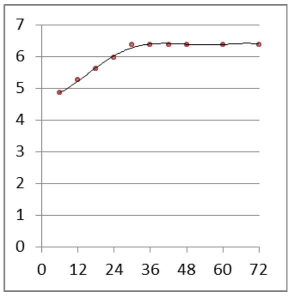

Table 4.2.2B: Detailed Scenario Operating Characteristics

	Shockable Rhythm				Non-Shockable Rhythm			
	Mean Weighted mRS	N	Pr (Target)	Pr (Target & Eff)	Mean Weighted mRS	N	Pr (Target)	Pr (Target & Eff)
6 Hour	4.85	9.8	0.015	0.000	4.85	9.1	0.015	0.000
12 Hour	5.27	96.4	0.021	0.018	5.27	97	0.023	0.017
18 Hour	5.62	90.5	0.078	0.071	5.62	90.5	0.077	0.071
24 Hour	*5.95*	*150.3*	*0.113*	*0.105*	*5.95*	*150.5*	*0.127*	*0.119*
30 Hour	*6.38*	*141.8*	*0.286*	*0.273*	*6.38*	*140.9*	*0.252*	*0.242*
36 Hour	*6.38*	*137.8*	*0.250*	*0.242*	*6.38*	*137.5*	*0.258*	*0.250*
42 Hour	*6.38*	*116.3*	*0.131*	*0.125*	*6.38*	*116.8*	*0.144*	*0.138*
48 Hour	6.38	125.4	0.080	0.076	6.38	125.9	0.073	0.070
60 Hour	6.38	28.2	0.025	0.024	6.38	28.6	0.028	0.028
72 Hour	6.38	3.2	0.002	0.002	6.38	3.5	0.004	0.004

4.2.3 U-Shaped Duration Response (Scenario 41.41)

In this scenario, increased cooling duration provides a strong treatment effect, and in contrast to the two scenarios above (Section 4.2.1 and 4.2.2), the duration-response curve is U-shaped. The target duration is 24 hours, the benefit of cooling plateaus between 24 and 36 hours, and then begins to decrease beyond 36 hours. The 6-hour arm is enrolled to with 53% probability. The duration-response model is able to identify the decrease in treatment benefit after 36 hours and as a result the 60-hour arm is enrolled to with only 1% probability and the 72-hour arm was never enrolled to in any of the simulated trials. The trial continues to completion with 98% probability (i.e. will stop early for futility less than 2% of the time).

The 18-, 24-, 30-, and 36-hour arms are considered clinically acceptable and are selected as the target duration with 83% probability. The positive duration response curve is identified with 70% probability.

Table 4.2.3A: Summary Scenario Operating Characteristics

	Shockable	Non-Shockable
Pr(Select Acceptable Target Duration)	0.832	0.824
Pr(Positive Duration Response)	0.709	0.699
Pr(Futility)	0.013	0.018
Pr(Open 6 Hours)	0.514	0.540
Pr(Open 60 Hours)	0.012	0.014
Pr(Open 72 Hours)	0.000	0.000

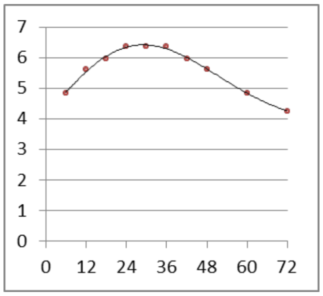

Table 4.2.3B: Detailed Scenario Operating Characteristics

	Shockable Rhythm				Non-Shockable Rhythm			
	Mean Weighted mRS	N	Pr (Target)	Pr (Target & Eff)	Mean Weighted mRS	N	Pr (Target)	Pr (Target & Eff)
6 Hour	4.85	24.9	0.046	0.000	4.85	26.1	0.051	0.000
12 Hour	5.62	157.5	0.120	0.062	5.62	158.2	0.122	0.062
18 Hour	*5.95*	*154.6*	*0.249*	*0.187*	*5.95*	*153.7*	*0.251*	*0.187*
24 Hour	*6.38*	*194.4*	*0.342*	*0.269*	*6.38*	*192.5*	*0.340*	*0.264*
30 Hour	*6.38*	*132.0*	*0.189*	*0.151*	*6.38*	*131.4*	*0.183*	*0.148*
36 Hour	*6.38*	*97.1*	*0.052*	*0.038*	*6.38*	*96.0*	*0.050*	*0.036*
42 Hour	5.95	65.6	0.003	0.002	5.95	65.1	0.003	0.002
48 Hour	5.62	75.1	0.000	0.000	5.62	74.8	0.000	0.000
60 Hour	4.85	0.4	0.000	0.000	4.85	0.4	0.000	0.000
72 Hour	4.25	0.0	0.000	0.000	4.25	0.0	0.000	0.000

4.2.4 Increasing Duration Response (Scenario 42.42)

In this scenario, increased cooling duration has a strong treatment effect and the duration-response curve is increasing across all arms. The optimal duration is 72 hours, though the difference in mean weighted mRS is small between the 60-hour and 72-hour duration arms. The 6-hour duration arm is enrolled to with 32% probability, and the 60-hour arm is enrolled to with 70% probability. The 72-hour arm can only be opened after the 60-hour arm has been opened, and therefore, the 72-hour arm is enrolled to with lower probability, 41% probability. The trial continues to completion with 98% probability (i.e. will stop early for futility less than 2% of the time). The 48-hour arm has the largest mean sample size, the result of additional criteria having to be met before patients can be enrolled to the 60- or 72- hour duration arms. However, the duration-response model is able to identify the increasing shape of the curve. The 72-hour and 60-hour duration arms are both considered clinically acceptable and they are selected as the target duration with 40% probability in each rhythm type. The positive duration response curve is identified with 85% probability.

Table 4.2.4A: Summary Scenario Operating Characteristics

	Shockable	Non-Shockable
Pr(Select Acceptable Target Duration)	0.390	0.396
Pr(Positive Duration Response)	0.847	0.845
Pr(Futility)	0.015	0.020
Pr(Open 6 Hours)	0.318	0.333
Pr(Open 60 Hours)	0.698	0.698
Pr(Open 72 Hours)	0.413	0.412

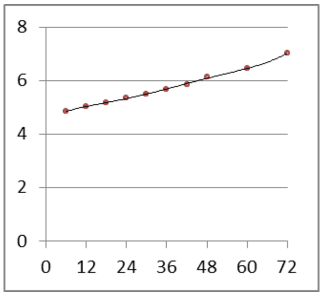

Table 4.2.4B: Detailed Scenario Operating Characteristics

	Shockable Rhythm				Non-Shockable Rhythm			
	Mean Weighted mRS	N	Pr (Target)	Pr (Target & Eff)	Mean Weighted mRS	N	Pr (Target)	Pr (Target & Eff)
6 Hour	4.85	17.6	0.048	0.000	4.85	19.0	0.047	0.000
12 Hour	5.015	113.0	0.072	0.043	5.015	113.0	0.058	0.035
18 Hour	5.18	85.3	0.051	0.039	5.18	85.5	0.056	0.042
24 Hour	5.345	121.7	0.057	0.047	5.345	121.6	0.057	0.048
30 Hour	5.51	95.0	0.060	0.053	5.51	94.7	0.065	0.054
36 Hour	5.675	103.3	0.064	0.058	5.675	102.6	0.070	0.063
42 Hour	5.84	111.5	0.087	0.074	5.84	110.8	0.089	0.078
48 Hour	6.14	150.4	0.171	0.154	6.14	149.5	0.162	0.142
60 Hour	*6.44*	*68.1*	*0.183*	*0.172*	*6.44*	*66.4*	*0.191*	*0.179*
72 Hour	*7.04*	*35.7*	*0.207*	*0.207*	*7.04*	*35.2*	*0.205*	*0.204*

4.2.5 Large Treatment Effect at 48 Hours (Scenario 53.53)

In this scenario, increased cooling duration has a large treatment effect and the duration-response curve plateaus at 48 hours. The 6-hour arm is enrolled to with 14% probability, the 60-hour arm is enrolled to with 94% probability, and the 72-hour arm is enrolled to with 70% probability. The trial continues to completion with greater than 99% probability (i.e. will stop early for futility less than 1% of the time). The 48-hour arm has the largest mean sample size. In previous scenarios with a plateau duration-response curve, the two arms neighboring the target duration also had the larger sample sizes, but in this scenario, the 60-hour arm has a smaller mean sample size because of the additional criteria that must be satisfied before enrolling patients to that arm.

The 42-, 48-, and 60-hour arms are considered clinically acceptable and they are selected with 77% probability. The positive duration-response curve is identified with 99% probability.

Table 4.2.5A: Summary Scenario Operating Characteristics

	Shockable	Non-Shockable
Pr(Select Acceptable Target Duration)	0.777	0.763
Pr(Positive Duration Response)	0.994	0.993
Pr(Futility)	0.002	0.002
Pr(Open 6 Hours)	0.145	0.130
Pr(Open 60 Hours)	0.942	0.931
Pr(Open 72 Hours)	0.701	0.685

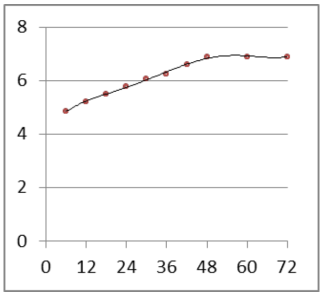

Table 4.2.5B: Detailed Scenario Operating Characteristics

	Shockable Rhythm				Non-Shockable Rhythm			
	Mean Weighted mRS	N	Pr (Target)	Pr (Target & Eff)	Mean Weighted mRS	N	Pr (Target)	Pr (Target & Eff)
6 Hour	4.85	4.5	0.003	0.000	4.85	4.2	0.004	0.000
12 Hour	5.2	70.3	0.003	0.003	5.2	70.0	0.001	0.001
18 Hour	5.51	52.2	0.022	0.022	5.51	53.1	0.021	0.021
24 Hour	5.77	98.1	0.035	0.035	5.77	99.7	0.033	0.033
30 Hour	6.08	83.3	0.050	0.049	6.08	83.9	0.051	0.050
36 Hour	6.24	106.1	0.066	0.066	6.24	106.9	0.072	0.072
42 Hour	*6.59*	*135.6*	*0.109*	*0.109*	*6.59*	*135.2*	*0.121*	*0.121*
48 Hour	*6.9*	*196.9*	*0.356*	*0.356*	*6.9*	*195.7*	*0.332*	*0.331*
60 Hour	*6.9*	*113.1*	*0.312*	*0.311*	*6.9*	*112.1*	*0.310*	*0.310*
72 Hour	6.9	39.8	0.044	0.044	6.9	39.4	0.054	0.054

4.2.6 Cooling Longer than 6 Hours is Harmful (Scenario 99.99)

In this scenario, increasing cooling duration is associated with a decrease in the mean weighted mRS. The 6-hour arm was enrolled to in more than 99% of simulated trials and the 60- and 72-hour duration arms were enrolled 0.2% or less of the simulated trials. Both rhythm types stop for futility over 96% of the time.

Table 4.2.6A: Summary Scenario Operating Characteristics

	Shockable	Non-Shockable
Pr(Select Acceptable Target Duration)	0.926	0.922
Pr(Positive Duration Response)	0.000	0.000
Pr(Futility)	0.968	0.966
Pr(Open 6 Hours)	0.998	0.998
Pr(Open 60 Hours)	0.002	0.001
Pr(Open 72 Hours)	0.000	0.000

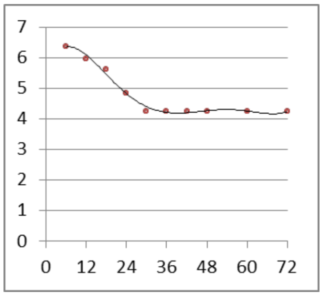

Table 4.2.6B: Detailed Scenario Operating Characteristics

	Shockable Rhythm				Non-Shockable Rhythm			
	**Mean Weighted mRS**	**N**	**Pr (Target)**	**Pr (Target & Eff)**	**Mean Weighted mRS**	**N**	**Pr (Target)**	**Pr (Target & Eff)**
6 Hour	*6.38*	*112.0*	*0.926*	*0.000*	*6.38*	*112.1*	*0.922*	*0.000*
12 Hour	5.95	198.2	0.069	0.000	5.95	201.9	0.077	0.000
18 Hour	5.62	98.1	0.005	0.000	5.62	99.3	0.002	0.000
24 Hour	4.85	86.2	0.000	0.000	4.85	86.9	0.000	0.000
30 Hour	4.25	38.6	0.000	0.000	4.25	39.4	0.000	0.000
36 Hour	4.25	31.0	0.000	0.000	4.25	31.7	0.000	0.000
42 Hour	4.25	23.3	0.000	0.000	4.25	23.9	0.000	0.000
48 Hour	4.25	48.9	0.000	0.000	4.25	49.4	0.000	0.000
60 Hour	4.25	0.1	0.000	0.000	4.25	0.1	0.000	0.000
72 Hour	4.25	0.0	0.000	0.000	4.25	0.0	0.000	0.000

4.2.7 Treatment Effect at 18 Hours (Scenario 31.31)

In this scenario, increasing cooling duration provides a strong treatment effect and the duration response curve plateaus at 18 hours. The 6-hour arm is enrolled to with 42% probability, the 60-hour arm is enrolled to with 16% probability, and the 72-hour arm is enrolled to with 2% probability. The trial continues to completion with greater than 99% probability (i.e. will stop early for futility less than 1% of the time).

The 24-hour duration, and the two shorter arms, 12- and 18-hours have the largest sample sizes. This allocation aids in identifying the treatment effect plateau. The 18-, 24-, and 30-hour durations are considered clinically acceptable. These arms are selected as the target duration with approximately 80% probability in each rhythm type. The positive duration-response curve is identified with 77% probability.

Table 4.2.7A: Summary Scenario Operating Characteristics

	Shockable	Non-Shockable
Pr(Select Acceptable Target Duration)	0.802	0.792
Pr(Positive Duration Response)	0.778	0.774
Pr(Futility)	0.003	0.003
Pr(Open 6 Hours)	0.425	0.420
Pr(Open 60 Hours)	0.171	0.158
Pr(Open 72 Hours)	0.017	0.020

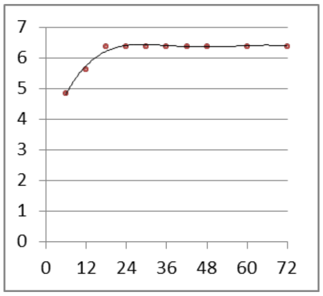

Table 4.2.7B: Detailed Scenario Operating Characteristics

	Shockable Rhythm				Non-Shockable Rhythm			
	Mean Weighted mRS	N	Pr (Target)	Pr (Target & Eff)	Mean Weighted mRS	N	Pr (Target)	Pr (Target & Eff)
6 Hour	4.85	16.8	0.038	0.000	4.85	16.3	0.038	0.000
12 Hour	5.615	133.2	0.059	0.035	5.615	132.2	0.062	0.034
18 Hour	*6.38*	*170.0*	*0.416*	*0.336*	*6.38*	*170.8*	*0.406*	*0.333*
24 Hour	*6.38*	*188.0*	*0.306*	*0.256*	*6.38*	*189.7*	*0.307*	*0.248*
30 Hour	*6.38*	*114.5*	*0.080*	*0.070*	*6.38*	*114.6*	*0.079*	*0.070*
36 Hour	6.38	92.3	0.045	0.037	6.38	92.3	0.049	0.042
42 Hour	6.38	78.6	0.030	0.026	6.38	78.1	0.030	0.025
48 Hour	6.38	97.4	0.018	0.013	6.38	96.7	0.024	0.017
60 Hour	6.38	9.0	0.007	0.006	6.38	8.4	0.005	0.004
72 Hour	6.38	0.4	0.000	0.000	6.38	0.6	0.000	0.000

4.3 Duration-response curves are different between the two rhythm types

In these scenarios, the duration response curve for shockable rhythms is as described in section 4.2.2. Therefore, increased cooling duration provides a strong treatment effect and the duration response curve plateaus at 30 hours. We explore combining this scenario with varying scenarios for non-shockable rhythms. Duration response modeling, allocation, and identification of the target duration is separate between the two rhythm types. Therefore, these operating characteristics change for one rhythm type very little based on the duration-response curve in the opposite rhythm type. However, the final inference on whether the duration response curve is positive is based on a hierarchical model across the two rhythm types. The probability that the duration response curve is identified as positive in one rhythm type may be affected by the duration-response curve in the opposite rhythm type.

**Table 4.3 Section Summary**	**Shockable Rhythm**			**Non-Shockable Rhythm**			
	**SAT**	**Pos**	**Fut**		**SAT**	**Pos**	**Fut**
Strong Effect at 30 hours	0.791	0.844	0.002	Small Effect at 18 hours	0.650	0.730	0.024
Strong Effect at 30 hours	0.785	0.864	0.004	U-shaped duration response	0.820	0.799	0.020
Strong Effect at 30 hours	0.786	0.927	0.002	Increasing duration response	0.418	0.854	0.019
Strong Effect at 30 hours	0.769	0.961	0.003	Larger treatment effect at 48 hours	0.758	0.978	0.001
Strong Effect at 30 hours	0.837	0.536	0.004	Cooling longer than 6 hours is harmful	0.913	0.000	0.920
Strong Effect at 30 hours	0.781	0.879	0.004	Treatment effect at 18 hours	0.797	0.852	0.006

SAT = Pr(Select Acceptable Target Duration)
Pos = Pr(Positive Duration Response)
Fut = Pr(Futility)

4.3.1 Non-Shockable Small Treatment Effect at 18 Hours (Scenario 32.21)

In this scenario, increased cooling duration provides a small treatment effect in the non-shockable rhythm type (as in Section 4.2.1). Therefore, there is borrowing between duration-response curves where one has a strong treatment effect and one has a smaller treatment effect. As compared to section 4.2, in shockable rhythms the probability that the duration response curve is identified as positive is decreased from 94% to 84%. In non-shockable rhythms, the probability that the duration response curve is identified is increased from 55% to 73%.

Table 4.3.1A: Summary Scenario Operating Characteristics

	Shockable	Non-Shockable
Pr(Select Acceptable Target Duration)	0.791	0.650
Pr(Positive Duration Response)	0.844	0.730
Pr(Futility)	0.002	0.024
Pr(Open 6 Hours)	0.232	0.512
Pr(Open 60 Hours)	0.460	0.134
Pr(Open 72 Hours)	0.093	0.015

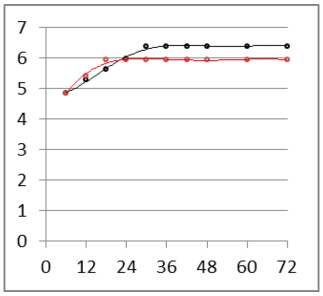

Table 4.3.1B: Detailed Scenario Operating Characteristics

	Shockable Rhythm				Non-Shockable Rhythm			
	**Mean Weighted mRS**	**N**	**Pr (Target)**	**Pr (Target & Eff)**	**Mean Weighted mRS**	**N**	**Pr (Target)**	**Pr (Target & Eff)**
6 Hour	4.85	8.9	0.020	0.000	4.85	27.5	0.075	0.000
12 Hour	5.27	96.4	0.019	0.008	5.39	155.5	0.162	0.095
18 Hour	5.62	91.2	0.060	0.050	*5.93*	*149.5*	*0.340*	*0.274*
24 Hour	*5.95*	*152.2*	*0.127*	*0.109*	*5.93*	*168.2*	*0.226*	*0.190*
30 Hour	*6.38*	*144.2*	*0.288*	*0.255*	*5.93*	*109.9*	*0.084*	*0.079*
36 Hour	*6.38*	*139.0*	*0.250*	*0.218*	5.93	94.3	0.046	0.040
42 Hour	*6.38*	*117.0*	*0.126*	*0.112*	5.93	82.1	0.038	0.029
48 Hour	6.38	125.3	0.088	0.072	5.93	100.5	0.021	0.018
60 Hour	6.38	27.2	0.022	0.019	5.93	7.2	0.007	0.004
72 Hour	6.38	3.4	0.001	0.001	5.93	0.5	0.000	0.000

4.3.2 U-Shaped Duration Response (Scenario 32.41)

In this scenario, in non-shockable rhythms, increased cooling duration provides a strong treatment effect, and the duration response curve is U-shaped (as in Section 4.2.3). As compared to section 4.2 above, in shockable rhythms the probability that the duration response curve is identified as positive is decreased from 94% to 86%. In non-shockable rhythms, the probability that the duration response curve is identified as positive is increased from 70% to 80%.

Table 4.3.2A: Summary Scenario Operating Characteristics

	Shockable	Non-Shockable
Pr(Select Acceptable Target Duration)	0.785	0.820
Pr(Positive Duration Response)	0.864	0.799
Pr(Futility)	0.004	0.020
Pr(Open 6 Hours)	0.252	0.541
Pr(Open 60 Hours)	0.486	0.015
Pr(Open 72 Hours)	0.088	0.000

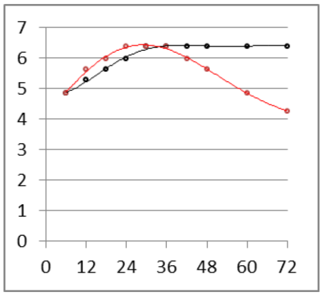

Table 4.3.2B: Detailed Scenario Operating Characteristics

	**Shockable Rhythm**				**Non-Shockable Rhythm**			
	**Mean Weighted mRS**	**N**	**Pr (Target)**	**Pr (Target & Eff)**	**Mean Weighted mRS**	**N**	**Pr (Target)**	**Pr (Target & Eff)**
6 Hour	4.85	9.5	0.016	0.000	4.85	25.7	0.064	0.000
12 Hour	5.27	97.3	0.020	0.011	5.62	155.4	0.116	0.084
18 Hour	5.62	90.6	0.067	0.054	*5.95*	*153.5*	*0.236*	*0.195*
24 Hour	*5.95*	*150.7*	*0.114*	*0.104*	*6.38*	*193.8*	*0.347*	*0.310*
30 Hour	*6.38*	*143.2*	*0.275*	*0.251*	*6.38*	*132.1*	*0.182*	*0.158*
36 Hour	*6.38*	*138.8*	*0.266*	*0.242*	*6.38*	*96.5*	*0.055*	*0.051*
42 Hour	*6.38*	*116.7*	*0.130*	*0.114*	5.95	65.2	0.002	0.002
48 Hour	6.38	125.5	0.082	0.065	5.62	74.5	0.000	0.000
60 Hour	6.38	27.6	0.027	0.021	4.85	0.5	0.000	0.000
72 Hour	6.38	3.1	0.002	0.002	4.25	0.0	0.000	0.000

4.3.3 Increasing Duration Response (Scenario 32.42)

In this scenario, for non-shockable rhythms, increased cooling duration has a strong treatment effect and the duration-response curve is increasing across all arms (as in Section 4.2.4). Operating characteristics are similar as compared to section 4.2 above.

Table 4.3.3A: Summary Scenario Operating Characteristics

	Shockable	Non-Shockable
Pr(Select Acceptable Target Duration)	0.786	0.418
Pr(Positive Duration Response)	0.927	0.854
Pr(Futility)	0.002	0.019
Pr(Open 6 Hours)	0.253	0.326
Pr(Open 60 Hours)	0.481	0.708
Pr(Open 72 Hours)	0.110	0.444

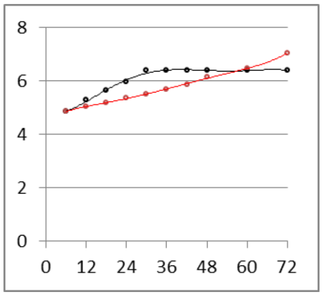

Table 4.3.3B: Detailed Scenario Operating Characteristics

	Shockable Rhythm				Non-Shockable Rhythm			
	Mean Weighted mRS	N	Pr (Target)	Pr (Target & Eff)	Mean Weighted mRS	N	Pr (Target)	Pr (Target & Eff)
6 Hour	4.85	9.6	0.013	0.000	4.85	17.5	0.050	0.000
12 Hour	5.27	96.8	0.017	0.011	5.015	109.8	0.058	0.033
18 Hour	5.62	91.4	0.071	0.063	5.18	84.4	0.059	0.052
24 Hour	*5.95*	*151.6*	*0.130*	*0.120*	5.345	119.9	0.055	0.048
30 Hour	*6.38*	*143.1*	*0.274*	*0.259*	5.51	92.8	0.053	0.049
36 Hour	*6.38*	*138.2*	*0.246*	*0.238*	5.675	101.8	0.062	0.056
42 Hour	*6.38*	*116.6*	*0.136*	*0.129*	5.84	110.8	0.088	0.081
48 Hour	6.38	126.0	0.082	0.076	6.14	150.4	0.156	0.136
60 Hour	6.38	27.9	0.031	0.030	*6.44*	*70.0*	*0.198*	*0.182*
72 Hour	6.38	3.5	0.001	0.001	*7.04*	*37.8*	*0.220*	*0.216*

4.3.4 Large Treatment Effects at 48 Hours (Scenario 32.53)

In this scenario, for non-shockable rhythms, increased cooling duration has a large treatment effect and the duration-response curve plateaus at 48 hours. In both rhythm types there is a high probabilities that the duration response curve is identified as positive change little as compared to section 4.2 above.

Table 4.3.4A: Summary Scenario Operating Characteristics

	Shockable	Non-Shockable
Pr(Select Acceptable Target Duration)	0.769	0.758
Pr(Positive Duration Response)	0.961	0.978
Pr(Futility)	0.003	0.001
Pr(Open 6 Hours)	0.247	0.148
Pr(Open 60 Hours)	0.477	0.931
Pr(Open 72 Hours)	0.110	0.682

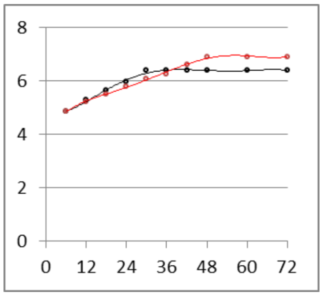

Table 4.3.4B: Detailed Scenario Operating Characteristics

	Shockable Rhythm				Non-Shockable Rhythm			
	Mean Weighted mRS	N	Pr (Target)	Pr (Target & Eff)	Mean Weighted mRS	N	Pr (Target)	Pr (Target & Eff)
6 Hour	4.85	9.6	0.018	0.000	4.85	4.6	0.003	0.000
12 Hour	5.27	97.2	0.021	0.018	5.2	71.1	0.006	0.005
18 Hour	5.62	91.6	0.073	0.068	5.51	53.7	0.030	0.029
24 Hour	*5.95*	*151.6*	*0.118*	*0.116*	5.77	99.9	0.039	0.037
30 Hour	*6.38*	*142.0*	*0.287*	*0.283*	6.08	84.8	0.054	0.054
36 Hour	*6.38*	*136.9*	*0.246*	*0.242*	6.24	107.2	0.058	0.057
42 Hour	*6.38*	*115.1*	*0.118*	*0.114*	*6.59*	*135.0*	*0.104*	*0.101*
48 Hour	6.38	125.0	0.089	0.089	*6.90*	*193.9*	*0.346*	*0.342*
60 Hour	6.38	28.2	0.029	0.029	*6.90*	*110.4*	*0.308*	*0.303*
72 Hour	6.38	3.5	0.002	0.002	6.90	38.8	0.051	0.050

4.3.5 Cooling Past 6 Hours in Harmful (Scenario 32.99)

In this scenario, increasing cooling duration results in decreasing weighted mean mRS in non-shockable rhythms. The trial stops for futility with 92% probability in non-shockable rhythms. The poor performance of additional cooling in non-shockable rhythms creates a stark interaction between duration and efficacy. This lowers the probability of determining that the duration-response is positive in shockable rhythms to 54% from 94% in the case when both arms have the same profile as the shockable rhythms.

Table 4.3.5A: Summary Scenario Operating Characteristics

	Shockable	Non-Shockable
Pr(Select Acceptable Target Duration)	0.837	0.913
Pr(Positive Duration Response)	0.536	0.000
Pr(Futility)	0.004	0.920
Pr(Open 6 Hours)	0.260	0.998
Pr(Open 60 Hours)	0.496	0.002
Pr(Open 72 Hours)	0.109	0.000

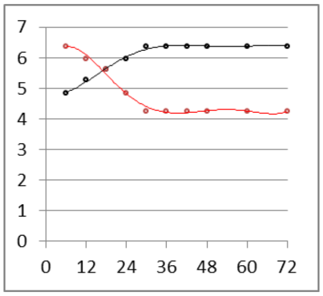

Table 4.3.5B: Detailed Scenario Operating Characteristics

	Shockable Rhythm				Non-Shockable Rhythm			
	Mean Weighted mRS	N	Pr (Target)	Pr (Target & Eff)	Mean Weighted mRS	N	Pr (Target)	Pr (Target & Eff)
6 Hour	4.85	12.2	0.006	0.000	*6.38*	*97.6*	*0.913*	*0.000*
12 Hour	5.27	106.2	0.008	0.001	5.95	185.8	0.085	0.000
18 Hour	5.62	113.4	0.048	0.017	5.62	91.4	0.002	0.000
24 Hour	*5.95*	*191.7*	*0.114*	*0.066*	4.85	84.1	0.000	0.000
30 Hour	*6.38*	*207.7*	*0.306*	*0.169*	4.25	37.8	0.000	0.000
36 Hour	*6.38*	*201.1*	*0.292*	*0.170*	4.25	30.5	0.000	0.000
42 Hour	*6.38*	*161.6*	*0.125*	*0.061*	4.25	23.0	0.000	0.000
48 Hour	6.38	158.5	0.074	0.034	4.25	48.9	0.000	0.000
60 Hour	6.38	41.0	0.025	0.016	4.25	0.1	0.000	0.000
72 Hour	6.38	5.6	0.002	0.002	4.25	0.0	0.000	0.000

4.3.6 Treatment Effect at 18 Hours (Scenario 32.31)

In this scenario, for non-shockable rhythms, the duration-response curve plateaus at 18 hours, while it plateaus as 30 hours for shockable rhythms. The operating characteristics for each scenario are similar to when the effects are the same in each groups – showing that small deviations in effect across durations for the 2 rhythm types does not have a detrimental effect.

Table 4.3.6A: Summary Scenario Operating Characteristics

	Shockable	Non-Shockable
Pr(Select Acceptable Target Duration)	0.781	0.797
Pr(Positive Duration Response)	0.879	0.852
Pr(Futility)	0.004	0.006
Pr(Open 6 Hours)	0.264	0.404
Pr(Open 60 Hours)	0.466	0.155
Pr(Open 72 Hours)	0.090	0.020

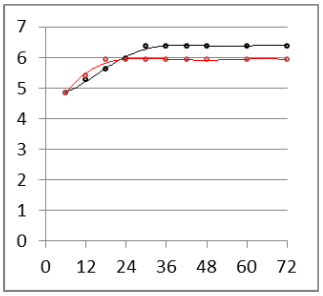

Table 4.3.6B: Detailed Scenario Operating Characteristics

	Shockable Rhythm				Non-Shockable Rhythm			
	Mean Weighted mRS	N	Pr (Target)	Pr (Target & Eff)	Mean Weighted mRS	N	Pr (Target)	Pr (Target & Eff)
6 Hour	4.85	10.0	0.018	0.000	4.85	16.3	0.036	0.000
12 Hour	5.27	97.8	0.023	0.012	5.39	133.5	0.068	0.050
18 Hour	5.62	92.8	0.070	0.061	*5.93*	*170.0*	*0.423*	*0.371*
24 Hour	*5.95*	*151.7*	*0.124*	*0.106*	*5.93*	*186.7*	*0.284*	*0.255*
30 Hour	*6.38*	*142.2*	*0.271*	*0.252*	*5.93*	*114.2*	*0.090*	*0.084*
36 Hour	*6.38*	*137.4*	*0.254*	*0.237*	5.93	93.2	0.043	0.040
42 Hour	*6.38*	*115.1*	*0.132*	*0.120*	5.93	79.2	0.030	0.029
48 Hour	6.38	124.5	0.082	0.071	5.93	97.1	0.022	0.020
60 Hour	6.38	26.8	0.023	0.018	5.93	8.0	0.004	0.003
72 Hour	6.38	3.0	0.002	0.002	5.93	0.6	0.000	0.000

4.4 Sensitivity Analysis on Proportion of Patients in Each Rhythm Type

In this section, we present a sensitivity analysis on the proportion of patients in each rhythm type. Section 4.2 and 4.3 assumed 50% of patients in each rhythm type. In this section, we assume 75% of patients will have shockable rhythms. In general, the mean sample size is increased in shockable rhythms and correspondingly, the probabilities of success increase. The mean sample size in non-shockable rhythms is decreased and correspondingly the probabilities of success decrease. Results are generalizable to the converse – assuming 75% of patients will have non-shockable rhythm.

	Shockable			Non-Shockable		
	**Selected Acceptable Target Duration**	**Positive Duration Response**	**Futility**	**Selected Acceptable Target Duration**	**Positive Duration Response**	**Futility**
Small Effect at 18 hours	0.730	0.592	0.034	0.520	0.402	0.002
Strong Effect at 30 hours	0.839	0.951	0.004	0.678	0.852	0.001
U-shaped duration response	0.891	0.746	0.025	0.660	0.545	0.003
Increasing duration response	0.607	0.899	0.016	0.139	0.768	0.001
Larger treatment effect at 48 hours	0.789	0.997	0.001	0.644	0.956	0.001
Cooling > 6 hours is harmful	0.921	0.000	0.992	0.921	0.000	0.892
Treatment effect at 18 hours	0.843	0.791	0.010	0.666	0.624	0.002

4.4.1 Small Treatment Effect at 18 Hours (Scenario 21.21)

As compared to Section 4.2.1, the mean sample size in shockable rhythms increases from 900 to 1335. The probability that a clinically acceptable duration is selected increases from 64% to 73%. The probability of identifying the positive duration response curve increases from 56% to 59%. In non-shockable rhythms, the mean sample size decreases from 900 to 465. The probability that a clinically acceptable duration is selected decreases from 64% to 52% due to the smaller sample size.

Table 4.4.1A: Summary Scenario Operating Characteristics

	**Shockable**	**Non-Shockable**
Pr(Select Acceptable Target Duration)	0.730	0.520
Pr(Positive Duration Response)	0.592	0.402
Pr(Futility)	0.034	0.002
Pr(Open 6 Hours)	0.530	0.438
Pr(Open 60 Hours)	0.167	0.099
Pr(Open 72 Hours)	0.015	0.007

Table 4.4.1B: Detailed Scenario Operating Characteristics

	**Shockable Rhythm**				**Non-Shockable Rhythm**			
	**Mean Weighted mRS**	**N**	**Pr (Target)**	**Pr (Target & Eff)**	**Mean Weighted mRS**	**N**	**Pr (Target)**	**Pr (Target & Eff)**
6 Hour	4.85	36.7	0.058	0.000	4.85	12.9	0.146	0.000
12 Hour	5.39	216.6	0.104	0.042	5.39	86.3	0.230	0.064
18 Hour	*5.93*	*238.9*	*0.390*	*0.247*	*5.93*	*74.8*	*0.289*	*0.142*
24 Hour	*5.93*	*261.7*	*0.252*	*0.170*	*5.93*	*84.1*	*0.168*	*0.097*
30 Hour	*5.93*	*164.4*	*0.088*	*0.060*	*5.93*	*57.0*	*0.063*	*0.041*
36 Hour	5.93	137.2	0.039	0.029	5.93	50.2	0.043	0.026
42 Hour	5.93	118.9	0.032	0.020	5.93	44.2	0.037	0.022
48 Hour	5.93	147.5	0.030	0.019	5.93	52.6	0.021	0.009
60 Hour	5.93	12.5	0.008	0.005	5.93	2.7	0.002	0.000
72 Hour	5.93	0.7	0.000	0.000	5.93	0.1	0.000	0.000

4.4.2 Strong Treatment Effects at 30 Hours (Scenario 32.32)

As compared to section 4.2.2, the mean sample size in shockable rhythms increases from 900 to 1350 while the probability a clinically acceptable duration is selected increases from approximately 78% to 84%. In total, the probability of identifying the positive duration response curve was already high, 94%, under the assumption of equal proportions in each rhythm type so the increase in sample size changes this little, to 95%. In non-shockable rhythms, the mean sample size decreases from 900 to 451 and the probability of selecting a clinically acceptable duration decreases from approximately 78% to 68%. The probability of identifying the positive duration-response decreases from 94% to 85%.

Table 4.4.2A: Summary Scenario Operating Characteristics

	**Shockable**	**Non-Shockable**
Pr(Select Acceptable Target Duration)	0.839	0.678
Pr(Positive Duration Response)	0.951	0.852
Pr(Futility)	0.004	0.001
Pr(Open 6 Hours)	0.209	0.258
Pr(Open 60 Hours)	0.536	0.330
Pr(Open 72 Hours)	0.122	0.052

Table 4.4.2B: Detailed Scenario Operating Characteristics

	**Shockable Rhythm**				**Non-Shockable Rhythm**			
	**Mean Weighted mRS**	**N**	**Pr (Target)**	**Pr (Target & Eff)**	**Mean Weighted mRS**	**N**	**Pr (Target)**	**Pr (Target & Eff)**
6 Hour	4.85	9.5	0.006	0.000	4.85	5.8	0.061	0.000
12 Hour	5.27	120.5	0.007	0.005	5.27	62.6	0.070	0.048
18 Hour	5.62	117.9	0.051	0.046	5.62	53.9	0.103	0.086
24 Hour	*5.95*	*219.2*	*0.096*	*0.090*	*5.95*	*77.1*	*0.128*	*0.117*
30 Hour	*6.38*	*229.8*	*0.302*	*0.290*	*6.38*	*64.7*	*0.220*	*0.204*
36 Hour	*6.38*	*225.8*	*0.309*	*0.300*	*6.38*	*61.9*	*0.198*	*0.190*
42 Hour	*6.38*	*181.8*	*0.132*	*0.126*	*6.38*	*54.5*	*0.132*	*0.123*
48 Hour	6.38	188.6	0.061	0.059	6.38	61.0	0.074	0.070
60 Hour	6.38	48.4	0.030	0.029	6.38	9.2	0.014	0.012
72 Hour	6.38	6.9	0.006	0.006	6.38	0.8	0.000	0.000

4.4.3 U-Shaped Duration Response (Scenario 41.41)

As compared to section 4.2.3, the mean sample size in shockable rhythms increases from 900 to 1340 and the probability of selecting a clinically acceptable duration increases from 83% to 89% and the probability of identifying the positive duration response increases from 71% to 75%. In non-shockable rhythms, the mean sample size decreases from 900 to 460. The probability that a clinically acceptable duration is selected decreases from 82% to 66% and the probability of identifying a positive duration response decreases from 70% to 55%.

Table 4.4.3A: Summary Scenario Operating Characteristics

	**Shockable**	**Non-Shockable**
Pr(Select Acceptable Target Duration)	0.891	0.660
Pr(Positive Duration Response)	0.746	0.545
Pr(Futility)	0.025	0.003
Pr(Open 6 Hours)	0.541	0.442
Pr(Open 60 Hours)	0.013	0.024
Pr(Open 72 Hours)	0.000	0.000

Table 4.4.3B: Detailed Scenario Operating Characteristics

	**Shockable Rhythm**				**Non-Shockable Rhythm**			
	**Mean Weighted mRS**	**N**	**Pr (Target)**	**Pr (Target & Eff)**	**Mean Weighted mRS**	**N**	**Pr (Target)**	**Pr (Target & Eff)**
6 Hour	4.85	32.8	0.028	0.000	4.85	12.4	0.110	0.000
12 Hour	5.62	216.0	0.080	0.045	5.62	87.2	0.222	0.086
18 Hour	*5.95*	*231.6*	*0.221*	*0.163*	*5.95*	*75.0*	*0.233*	*0.143*
24 Hour	*6.38*	*311.5*	*0.387*	*0.309*	*6.38*	*90.9*	*0.242*	*0.180*
30 Hour	*6.38*	*211.3*	*0.237*	*0.193*	*6.38*	*63.0*	*0.139*	*0.103*
36 Hour	*6.38*	*144.2*	*0.046*	*0.035*	*6.38*	*50.0*	*0.046*	*0.030*
42 Hour	5.95	90.1	0.001	0.001	5.95	37.8	0.006	0.003
48 Hour	5.62	102.0	0.000	0.000	5.62	43.2	0.001	0.000
60 Hour	4.85	0.5	0.000	0.000	4.85	0.4	0.000	0.000
72 Hour	4.25	0.0	0.000	0.000	4.25	0.0	0.000	0.000

4.4.4 Increasing Duration Response (Scenario 42.42)

As compared to section 4.2.4, the mean sample size in shockable rhythms decreases from 900 to 1343. The probability that a clinically acceptable duration is selected is increased from 39% to 61% and the probability of identifying a positive duration-response increases from 85% to 90%. In non-shockable rhythms, the mean sample size decreases from 900 to 457. The probability a clinically acceptable duration is selected is decreased from 40% to 14% and the probability of identifying a positive duration response decreases from 85% to 77%.

Table 4.4.4A: Summary Scenario Operating Characteristics

	**Shockable**	**Non-Shockable**
Pr(Select Acceptable Target Duration)	0.607	0.139
Pr(Positive Duration Response)	0.899	0.768
Pr(Futility)	0.016	0.001
Pr(Open 6 Hours)	0.284	0.321
Pr(Open 60 Hours)	0.817	0.425
Pr(Open 72 Hours)	0.622	0.166

Table 4.4.4B: Detailed Scenario Operating Characteristics

	**Shockable Rhythm**				**Non-Shockable Rhythm**			
	**Mean Weighted mRS**	**N**	**Pr (Target)**	**Pr (Target & Eff)**	**Mean Weighted mRS**	**N**	**Pr (Target)**	**Pr (Target & Eff)**
6 Hour	4.85	19.8	0.020	0.000	4.85	9.5	0.107	0.000
12 Hour	5.015	142.1	0.029	0.016	5.015	70.5	0.145	0.096
18 Hour	5.18	107.2	0.039	0.032	5.18	54.4	0.097	0.076
24 Hour	5.345	163.7	0.036	0.031	5.345	70.3	0.099	0.084
30 Hour	5.51	125.4	0.049	0.042	5.51	53.7	0.086	0.077
36 Hour	5.675	141.4	0.047	0.042	5.675	54.4	0.071	0.067
42 Hour	5.84	159.5	0.055	0.044	5.84	54.5	0.100	0.092
48 Hour	6.14	229.8	0.118	0.101	6.14	67.9	0.157	0.142
60 Hour	*6.44*	*139.6*	*0.171*	*0.156*	*6.44*	*16.8*	*0.102*	*0.096*
72 Hour	*7.04*	*114.8*	*0.436*	*0.433*	*7.04*	*4.8*	*0.037*	*0.037*

4.4.5 Large Treatment Effect at 48 Hours (Scenario 53.53)

As compared to section 4.2.5, the mean sample size in shockable rhythms increases from 900 to 1350. Power for this scenario was already quite good so the increase in sample size has little effect. In non-shockable rhythms, the mean sample size in non-shockable rhythms decreases from 900 to 450. The probability a clinically acceptable duration is selected is decreased from 76% to 64% and the probability of identifying the positive duration-response decreases slightly from over 99% to 96%.

Table 4.4.5A: Summary Scenario Operating Characteristics

	**Shockable**	**Non-Shockable**
Pr(Select Acceptable Target Duration)	0.789	0.644
Pr(Positive Duration Response)	0.997	0.956
Pr(Futility)	0.001	0.001
Pr(Open 6 Hours)	0.097	0.169
Pr(Open 60 Hours)	0.975	0.752
Pr(Open 72 Hours)	0.849	0.359

Table 4.4.5B: Detailed Scenario Operating Characteristics

	**Shockable Rhythm**				**Non-Shockable Rhythm**			
	**Mean Weighted mRS**	**N**	**Pr (Target)**	**Pr (Target & Eff)**	**Mean Weighted mRS**	**N**	**Pr (Target)**	**Pr (Target & Eff)**
6 Hour	4.85	3.8	0.001	0.000	4.85	3.3	0.024	0.000
12 Hour	5.2	83.7	0.001	0.000	5.2	49.9	0.027	0.023
18 Hour	5.51	58.3	0.020	0.020	5.51	39.9	0.045	0.044
24 Hour	5.77	124.3	0.037	0.037	5.77	62.3	0.064	0.062
30 Hour	6.08	100.6	0.032	0.032	6.08	51.4	0.080	0.079
36 Hour	6.24	143.2	0.035	0.035	6.24	57.6	0.103	0.102
42 Hour	*6.59*	*202.3*	*0.080*	*0.079*	*6.59*	*64.0*	*0.152*	*0.150*
48 Hour	*6.9*	*325.8*	*0.348*	*0.347*	*6.9*	*82.2*	*0.320*	*0.316*
60 Hour	*6.9*	*219.0*	*0.361*	*0.361*	*6.9*	*32.3*	*0.172*	*0.170*
72 Hour	6.9	88.3	0.086	0.086	6.9	7.6	0.012	0.011

4.4.6 Cooling More Than 6 Hours is Harmful (Scenario 99.99)

In this scenario, the increased proportion of patients in shockable rhythms matters little. The trial stops early for futility with high probability and the mean total sample size increases only slightly in each rhythm type.

Table 4.4.6A: Summary Scenario Operating Characteristics

	**Shockable**	**Non-Shockable**
Pr(Select Acceptable Target Duration)	0.921	0.921
Pr(Positive Duration Response)	0.000	0.000
Pr(Futility)	0.992	0.892
Pr(Open 6 Hours)	1.000	0.989
Pr(Open 60 Hours)	0.000	0.001
Pr(Open 72 Hours)	0.000	0.000

Table 4.4.6B: Detailed Scenario Operating Characteristics

	**Shockable Rhythm**				**Non-Shockable Rhythm**			
	**Mean Weighted mRS**	**N**	**Pr (Target)**	**Pr (Target & Eff)**	**Mean Weighted mRS**	**N**	**Pr (Target)**	**Pr (Target & Eff)**
6 Hour	*6.38*	*112.2*	*0.921*	*0.000*	*6.38*	*118.1*	*0.921*	*0.000*
12 Hour	5.95	230.7	0.075	0.000	5.95	209.4	0.077	0.000
18 Hour	5.62	102.7	0.004	0.000	5.62	115.2	0.002	0.000
24 Hour	4.85	102.1	0.000	0.000	4.85	80.3	0.000	0.000
30 Hour	4.25	38.2	0.000	0.000	4.25	45.1	0.000	0.000
36 Hour	4.25	30.6	0.000	0.000	4.25	36.3	0.000	0.000
42 Hour	4.25	22.5	0.000	0.000	4.25	28.0	0.000	0.000
48 Hour	4.25	64.1	0.000	0.000	4.25	37.0	0.000	0.000
60 Hour	4.25	0.0	0.000	0.000	4.25	0.0	0.000	0.000
72 Hour	4.25	0.0	0.000	0.000	4.25	0.0	0.000	0.000

4.4.7 Treatment Effect at 18 Hours (31.31)

As compared to section 4.2.7, the mean sample size in shockable rhythms increases from 900 to 1345. The probability a clinically acceptable duration is selected increases from approximately 80% to 84% the probability of identifying the positive duration-response curve increases from 78% to only 79%. In non-shockable rhythms, the mean sample size decreases from 900 to 454. The probability of selecting a clinically acceptable duration decreases from approximately 79% to 67% and the probability of identifying the positive duration response curve decreases from 77% to 62%.

Table 4.4.7A: Summary Scenario Operating Characteristics

	**Shockable**	**Non-Shockable**
Pr(Select Acceptable Target Duration)	0.843	0.666
Pr(Positive Duration Response)	0.791	0.624
Pr(Futility)	0.010	0.002
Pr(Open 6 Hours)	0.404	0.390
Pr(Open 60 Hours)	0.188	0.125
Pr(Open 72 Hours)	0.026	0.012

Table 4.4.7B: Detailed Scenario Operating Characteristics

	**Shockable Rhythm**				**Non-Shockable Rhythm**			
	**Mean Weighted mRS**	**N**	**Pr (Target)**	**Pr (Target & Eff)**	**Mean Weighted mRS**	**N**	**Pr (Target)**	**Pr (Target & Eff)**
6 Hour	4.85	20.0	0.025	0.000	4.85	9.8	0.074	0.000
12 Hour	5.615	178.2	0.036	0.023	5.615	77.3	0.150	0.060
18 Hour	*6.38*	*276.8*	*0.442*	*0.360*	*6.38*	*76.1*	*0.349*	*0.244*
24 Hour	*6.38*	*297.2*	*0.321*	*0.261*	*6.38*	*86.9*	*0.239*	*0.178*
30 Hour	*6.38*	*171.1*	*0.080*	*0.067*	*6.38*	*57.3*	*0.078*	*0.061*
36 Hour	6.38	133.9	0.042	0.036	6.38	49.4	0.046	0.036
42 Hour	6.38	111.8	0.024	0.021	6.38	43.1	0.037	0.028
48 Hour	6.38	139.7	0.021	0.017	6.38	51.5	0.022	0.013
60 Hour	6.38	15.0	0.007	0.006	6.38	3.4	0.006	0.003
72 Hour	6.38	1.4	0.001	0.001	6.38	0.2	0.000	0.000

4.5 Varying the Proportion of Patients in Each Rhythm Type and the Effect Size in Each Rhythm Type

In this section, we present a sensitivity analysis, varying the proportion of patients and the effect of cooling in each rhythm type. Sections 4.3 and 4.4 vary each of these separately, this section varies each simultaneously. In this section, we vary the rate of patients with shockable rhythms from 10%, 30%, 50%, 70%, and 90%. We pair the strong effect at 30 hours for shockable rhythms with varying effects for the non-shockable rhythm types.

We first present an overall summary of these simulations, then present each scenario separately with the full operating characteristics. We assume the default accrual rate for each scenario in this section. A total of 2500 simulations were conducted per scenario. Table 4.5 presents the probability of an acceptable target duration being selected and the probability of a positive duration response for each rhythm type. The mean sample sizes for each rhythm type are also summarized.

Table 4.5: The summary operating characteristics for various combinations of different treatment effects and enrollment proportions. The # column refers to the labeling of each scenario for the back-up software output. The % for each row presents the proportion of patient enrolled from each rhythm type. The Name column refers to the scenario name presented throughout Section 4. The operating characteristics presented for each rhythm type are N: The mean sample size; SAP: The proportion of times selecting an acceptable target (SAT) dose; and Pos: The proportion of trials a statistically significant positive trend is determined.

Shockable						Non-Shockable					
#	%	Name	N	SAT	Pos	#	%	Name	N	SAT	Pos
32	10	Strong 30	354.1	.606	.306	1	90	Null	1445.9	.913	.104
32	30	Strong 30	646.1	.744	.550	1	70	Null	1153.4	.903	.202
32	50	Strong 30	941.7	.824	.748	1	50	Null	858.1	.889	.259
32	70	Strong 30	1246.1	.850	.827	1	30	Null	535.5	.872	.295
32	90	Strong 30	1617.9	.866	.914	1	10	Null	181.7	.859	.334

Shockable						Non-Shockable					
#	%	Name	N	SAT	Pos	#	%	Name	N	SAT	Pos
32	10	Strong 30	201.5	.544	.442	21	90	Small 18	1598.5	.771	.672
32	30	Strong 30	551.1	.737	.715	21	70	Small 18	1248.8	.730	.742
32	50	Strong 30	904.2	.816	.844	21	50	Small 18	895.8	.671	.733
32	70	Strong 30	1260.2	.845	.903	21	30	Small 18	539.8	.567	.672
32	90	Strong 30	1617.6	.858	0.92	21	10	Small 18	182.4	.428	.542

Shockable						Non-Shockable					
#	%	Name	N	SAT	Pos	#	%	Name	N	SAT	Pos
32	10	Strong 30	193.2	.541	.486	41	90	U-Shaped	1606.8	.920	.799
32	30	Strong 30	544.9	.741	.746	41	70	U-Shaped	1255.1	.903	.832
32	50	Strong 30	902.1	.811	.855	41	50	U-Shaped	897.9	.841	.814
32	70	Strong 30	1258.7	.837	.915	41	30	U-Shaped	541.3	.747	.756
32	90	Strong 30	1617.6	.866	.924	41	10	U-Shaped	182.3	.519	.548

Shockable						Non-Shockable					
#	%	Name	N	SAT	Pos	#	%	Name	N	SAT	Pos
32	10	Strong 30	190.9	.534	.719	42	90	Increasing	1609	.598	.902
32	30	Strong 30	546.6	.732	.872	42	70	Increasing	1253.4	.479	.887
32	50	Strong 30	903.5	.817	.908	42	50	Increasing	896.5	.276	.828
32	70	Strong 30	1259.1	.852	.944	42	30	Increasing	540.9	.114	.758

32	90	Strong 30	1617.1	.860	.940	42	10	Increasing	182.9	.011	.575

**Shockable**						**Non-Shockable**					
**#**	**%**	**Name**	N	SAT	Pos	**#**	**%**	**Name**	N	SAT	Pos
**32**	**10**	**Strong 30**	180.4	.535	.773	**53**	**90**	**Large 48**	1619.4	.788	.996
**32**	**30**	**Strong 30**	540.5	.734	.926	**53**	**70**	**Large 48**	1259.5	.813	.993
**32**	**50**	**Strong 30**	899.3	.811	.961	**53**	**50**	**Large 48**	900.7	.783	.978
**32**	**70**	**Strong 30**	1258.2	.842	.973	**53**	**30**	**Large 48**	540.9	.661	.932
**32**	**90**	**Strong 30**	1618.3	.879	.950	**53**	**10**	**Large 48**	181.8	.310	.743

The following subsections present the individual operating characteristics for each of the scenarios presented in Table 4.5.

4.5.1 10% Shockable: Strong Treatment Effects at 30 Hours with 90% Non-shockable: Null Treatment Effect (Scenario 32.1.1)

Table 4.5.1A: Summary Scenario Operating Characteristics

	**Shockable**	**Non-Shockable**
Pr(Select Acceptable Target Duration)	0.606	0.913
Pr(Positive Duration Response)	0.306	0.104
Pr(Futility)	0.000	0.354
Pr(Open 6 Hours)	0.152	0.836
Pr(Open 60 Hours)	0.150	0.039
Pr(Open 72 Hours)	0.013	0.003

Table 4.5.1B: Detailed Scenario Operating Characteristics

	**Shockable Rhythm**				**Non-Shockable Rhythm**			
	**Mean Weighted mRS**	**N**	**Pr (Target)**	**Pr (Target & Eff)**	**Mean Weighted mRS**	**N**	**Pr (Target)**	**Pr (Target & Eff)**
6 Hour	4.85	3.4	0.102	0.000	*4.85*	*137.4*	*0.458*	*0.000*
12 Hour	5.27	47.8	0.120	0.006	*4.85*	*336.3*	*0.382*	*0.045*
18 Hour	5.62	47.0	0.126	0.022	*4.85*	*214.9*	*0.073*	*0.023*
24 Hour	*5.95*	*60.2*	*0.150*	*0.046*	4.85	230.2	0.039	0.016
30 Hour	*6.38*	*57.3*	*0.181*	*0.082*	4.85	144.7	0.019	0.008
36 Hour	*6.38*	*53.5*	*0.178*	*0.088*	4.85	126.6	0.010	0.004
42 Hour	*6.38*	*43.1*	*0.097*	*0.042*	4.85	108.8	0.008	0.004
48 Hour	6.38	37.7	0.040	0.018	4.85	143.2	0.011	0.003
60 Hour	6.38	4.0	0.004	0.003	4.85	3.6	0.000	0.000
72 Hour	6.38	0.2	0.000	0.000	4.85	0.2	0.000	0.000
		354.1		0.306		1445.9		0.104

4.5.2 30% Shockable: Strong Treatment Effects at 30 Hours with 70% Non-shockable: Null Treatment Effect (Scenario 32.1.3)

Table 4.5.2A: Summary Scenario Operating Characteristics

	**Shockable**	**Non-Shockable**
Pr(Select Acceptable Target Duration)	0.744	0.903
Pr(Positive Duration Response)	0.550	0.202
Pr(Futility)	0.003	0.294
Pr(Open 6 Hours)	0.294	0.831
Pr(Open 60 Hours)	0.274	0.034
Pr(Open 72 Hours)	0.039	0.001

Table 4.5.2B: Detailed Scenario Operating Characteristics

	**Shockable Rhythm**				**Non-Shockable Rhythm**			
	**Mean Weighted mRS**	**N**	**Pr (Target)**	**Pr (Target & Eff)**	**Mean Weighted mRS**	**N**	**Pr (Target)**	**Pr (Target & Eff)**
6 Hour	4.85	9.7	0.042	0.000	*4.85*	*105.5*	*0.432*	*0.000*
12 Hour	5.27	79.3	0.038	0.006	*4.85*	*263.7*	*0.395*	*0.104*
18 Hour	5.62	75.2	0.092	0.034	*4.85*	*172.3*	*0.076*	*0.037*
24 Hour	*5.95*	*111.1*	*0.138*	*0.075*	4.85	184.5	0.042	0.023
30 Hour	*6.38*	*103.8*	*0.259*	*0.164*	4.85	118.8	0.020	0.014
36 Hour	*6.38*	*97.9*	*0.222*	*0.140*	4.85	104.7	0.015	0.011
42 Hour	*6.38*	*79.5*	*0.125*	*0.078*	4.85	88.8	0.012	0.008
48 Hour	6.38	78.4	0.067	0.040	4.85	112.8	0.008	0.006
60 Hour	6.38	10.4	0.016	0.013	4.85	2.3	0.000	0.000
72 Hour	6.38	0.9	0.000	0.000	4.85	0.1	0.000	0.000
		646		0.55		1153		0.202

4.5.3 50% Shockable: Strong Treatment Effects at 30 Hours with 50% Non-shockable: Null Treatment Effect (Scenario 32.1.5)

Table 4.5.3A: Summary Scenario Operating Characteristics

	**Shockable**	**Non-Shockable**
Pr(Select Acceptable Target Duration)	0.824	0.889
Pr(Positive Duration Response)	0.748	0.259
Pr(Futility)	0.003	0.191
Pr(Open 6 Hours)	0.264	0.774
Pr(Open 60 Hours)	0.349	0.043
Pr(Open 72 Hours)	0.054	0.004

Table 4.5.3B: Detailed Scenario Operating Characteristics

	**Shockable Rhythm**				**Non-Shockable Rhythm**			
	**Mean Weighted mRS**	**N**	**Pr (Target)**	**Pr (Target & Eff)**	**Mean Weighted mRS**	**N**	**Pr (Target)**	**Pr (Target & Eff)**
6 Hour	4.85	10.6	0.016	0.000	*4.85*	*72.6*	*0.423*	*0.000*
12 Hour	5.27	100.5	0.020	0.006	*4.85*	*194.4*	*0.390*	*0.136*
18 Hour	5.62	97.1	0.062	0.036	*4.85*	*129.6*	*0.076*	*0.041*
24 Hour	*5.95*	*160.1*	*0.112*	*0.080*	4.85	138.6	0.054	0.039
30 Hour	*6.38*	*157.5*	*0.313*	*0.241*	4.85	90.7	0.020	0.014
36 Hour	*6.38*	*152.4*	*0.282*	*0.229*	4.85	79.6	0.015	0.011
42 Hour	*6.38*	*122.2*	*0.117*	*0.094*	4.85	67.0	0.012	0.010
48 Hour	6.38	120.8	0.064	0.050	4.85	83.4	0.009	0.006
60 Hour	6.38	18.9	0.013	0.010	4.85	2	0.002	0.001
72 Hour	6.38	1.6	0.002	0.001	4.85	0.1	0.000	0.000
		942		0.748		858		0.259

4.5.4 70% Shockable: Strong Treatment Effects at 30 Hours with 30% Non-shockable: Null Treatment Effect (Scenario 32.1.7)

Table 4.5.4A: Summary Scenario Operating Characteristics

	**Shockable**	**Non-Shockable**
Pr(Select Acceptable Target Duration)	0.850	0.872
Pr(Positive Duration Response)	0.827	0.295
Pr(Futility)	0.004	0.074
Pr(Open 6 Hours)	0.235	0.699
Pr(Open 60 Hours)	0.426	0.040
Pr(Open 72 Hours)	0.082	0.001

Table 4.5.4B: Detailed Scenario Operating Characteristics

	**Shockable Rhythm**				**Non-Shockable Rhythm**			
	**Mean Weighted mRS**	**N**	**Pr (Target)**	**Pr (Target & Eff)**	**Mean Weighted mRS**	**N**	**Pr (Target)**	**Pr (Target & Eff)**
6 Hour	4.85	10.9	0.010	0.000	*4.85*	*38.8*	*0.407*	*0.000*
12 Hour	5.27	117.4	0.008	0.004	*4.85*	*120.3*	*0.374*	*0.145*
18 Hour	5.62	114.3	0.045	0.031	*4.85*	*82.4*	*0.091*	*0.056*
24 Hour	*5.95*	*209.8*	*0.104*	*0.080*	4.85	87.7	0.048	0.034
30 Hour	*6.38*	*217.9*	*0.297*	*0.254*	4.85	58.6	0.030	0.024
36 Hour	*6.38*	*214.4*	*0.308*	*0.267*	4.85	51.6	0.021	0.016
42 Hour	*6.38*	*171.1*	*0.141*	*0.120*	4.85	43	0.018	0.012
48 Hour	6.38	170.8	0.067	0.056	4.85	51.9	0.011	0.008
60 Hour	6.38	33.6	0.019	0.015	4.85	1.1	0.000	0.000
72 Hour	6.38	3.8	0.000	0.000	4.85	0	0.000	0.000
		1264		0.827		536		0.295

4.5.5 90% Shockable: Strong Treatment Effects at 30 Hours with 10% Non-shockable: Null Treatment Effect (Scenario 32.1.9)

Table 4.5.5A: Summary Scenario Operating Characteristics

	**Shockable**	**Non-Shockable**
Pr(Select Acceptable Target Duration)	0.866	0.859
Pr(Positive Duration Response)	0.914	0.334
Pr(Futility)	0.003	0.001
Pr(Open 6 Hours)	0.195	0.327
Pr(Open 60 Hours)	0.459	0.028
Pr(Open 72 Hours)	0.105	0.002

Table 4.5.5B: Detailed Scenario Operating Characteristics

	**Shockable Rhythm**				**Non-Shockable Rhythm**			
	**Mean Weighted mRS**	**N**	**Pr (Target)**	**Pr (Target & Eff)**	**Mean Weighted mRS**	**N**	**Pr (Target)**	**Pr (Target & Eff)**
6 Hour	4.85	9.9	0.004	0.000	*4.85*	*3.6*	*0.378*	*0.000*
12 Hour	5.27	131.3	0.004	0.002	*4.85*	*41.5*	*0.366*	*0.150*
18 Hour	5.62	130.2	0.035	0.031	*4.85*	*29.7*	*0.115*	*0.070*
24 Hour	*5.95*	*264.7*	*0.096*	*0.084*	4.85	31.9	0.060	0.046
30 Hour	*6.38*	*301.1*	*0.338*	*0.315*	4.85	22.2	0.033	0.026
36 Hour	*6.38*	*292.1*	*0.302*	*0.284*	4.85	19.1	0.024	0.020
42 Hour	*6.38*	*220.4*	*0.130*	*0.117*	4.85	15.5	0.018	0.015
48 Hour	6.38	215.6	0.064	0.058	4.85	17.8	0.007	0.006
60 Hour	6.38	46.6	0.026	0.022	4.85	0.3	0.000	0.000
72 Hour	6.38	5.9	0.001	0.001	4.85	0	0.000	0.000
		1618		0.914		182		0.334

4.5.6 10% Shockable: Strong Treatment Effects at 30 Hours with 90% Non-shockable: Small Treatment Effect at 18 Hours (Scenario 32.21.1)

Table 4.5.6A: Summary Scenario Operating Characteristics

	**Shockable**	**Non-Shockable**
Pr(Select Acceptable Target Duration)	0.544	0.771
Pr(Positive Duration Response)	0.442	0.672
Pr(Futility)	0.000	0.040
Pr(Open 6 Hours)	0.150	0.536
Pr(Open 60 Hours)	0.109	0.111
Pr(Open 72 Hours)	0.005	0.008

Table 4.5.6B: Detailed Scenario Operating Characteristics

	**Shockable Rhythm**				**Non-Shockable Rhythm**			
	**Mean Weighted mRS**	**N**	**Pr (Target)**	**Pr (Target & Eff)**	**Mean Weighted mRS**	**N**	**Pr (Target)**	**Pr (Target & Eff)**
6 Hour	4.85	1.4	0.132	0.000	4.85	42.4	0.046	0.000
12 Hour	5.27	33.1	0.152	0.024	5.39	252.9	0.078	0.041
18 Hour	5.62	28.0	0.142	0.054	*5.93*	*304.0*	*0.399*	*0.277*
24 Hour	*5.95*	*36.2*	*0.156*	*0.099*	*5.93*	*329.9*	*0.287*	*0.207*
30 Hour	*6.38*	*29.5*	*0.164*	*0.103*	*5.93*	*203.9*	*0.085*	*0.065*
36 Hour	*6.38*	*27.2*	*0.138*	*0.092*	5.93	163.0	0.046	0.038
42 Hour	*6.38*	*22.1*	*0.086*	*0.053*	5.93	133.4	0.031	0.022
48 Hour	6.38	22.7	0.028	0.016	5.93	159.1	0.024	0.018
60 Hour	6.38	1.3	0.001	0.001	5.93	9.4	0.004	0.004
72 Hour	6.38	0.0	0.000	0.000	5.93	0.5	0.000	0.000
		201.5		0.442		1598.5		0.672

4.5.7 30% Shockable: Strong Treatment Effects at 30 Hours with 70% Non-shockable: Small Treatment Effect at 18 Hours (Scenario 32.21.3)

Table 4.5.7A: Summary Scenario Operating Characteristics

	**Shockable**	**Non-Shockable**
Pr(Select Acceptable Target Duration)	0.737	0.730
Pr(Positive Duration Response)	0.715	0.742
Pr(Futility)	0.002	0.034
Pr(Open 6 Hours)	0.271	0.551
Pr(Open 60 Hours)	0.252	0.101
Pr(Open 72 Hours)	0.033	0.008

Table 4.5.7B: Detailed Scenario Operating Characteristics

	**Shockable Rhythm**				**Non-Shockable Rhythm**			
	**Mean Weighted mRS**	**N**	**Pr (Target)**	**Pr (Target & Eff)**	**Mean Weighted mRS**	**N**	**Pr (Target)**	**Pr (Target & Eff)**
6 Hour	4.85	7.6	0.047	0.000	4.85	38.2	0.050	0.000
12 Hour	5.27	73.6	0.052	0.018	5.39	209.1	0.117	0.066
18 Hour	5.62	66.3	0.095	0.063	*5.93*	*228.5*	*0.367*	*0.287*
24 Hour	*5.95*	*96.4*	*0.139*	*0.099*	*5.93*	*248.8*	*0.273*	*0.222*
30 Hour	*6.38*	*84.4*	*0.230*	*0.188*	*5.93*	*157.7*	*0.090*	*0.076*
36 Hour	*6.38*	*80.5*	*0.235*	*0.191*	5.93	129.1	0.045	0.040
42 Hour	*6.38*	*66.4*	*0.133*	*0.104*	5.93	106	0.034	0.029
48 Hour	6.38	68	0.057	0.041	5.93	124.8	0.024	0.020
60 Hour	6.38	7.3	0.012	0.011	5.93	6.2	0.002	0.002
72 Hour	6.38	0.6	0.000	0.000	5.93	0.2	0.000	0.000
		551		0.715		1248		0.742

4.5.8 *50% Shockable: Strong Treatment Effects at 30 Hours with 50% Non-shockable: Small Treatment Effect at 18 Hours (Scenario 32.21.5)*

Table 4.5.8A: Summary Scenario Operating Characteristics

	**Shockable**	**Non-Shockable**
Pr(Select Acceptable Target Duration)	0.816	0.671
Pr(Positive Duration Response)	0.844	0.733
Pr(Futility)	0.004	0.026
Pr(Open 6 Hours)	0.259	0.560
Pr(Open 60 Hours)	0.354	0.078
Pr(Open 72 Hours)	0.049	0.005

Table 4.5.8B: Detailed Scenario Operating Characteristics

	**Shockable Rhythm**				**Non-Shockable Rhythm**			
	**Mean Weighted mRS**	**N**	**Pr (Target)**	**Pr (Target & Eff)**	**Mean Weighted mRS**	**N**	**Pr (Target)**	**Pr (Target & Eff)**
6 Hour	4.85	10.1	0.017	0.000	4.85	30.5	0.086	0.000
12 Hour	5.27	99.1	0.014	0.006	5.39	159.9	0.148	0.092
18 Hour	5.62	94.3	0.067	0.054	*5.93*	*159.2*	*0.361*	*0.294*
24 Hour	*5.95*	*156.3*	*0.128*	*0.112*	*5.93*	*173.5*	*0.229*	*0.193*
30 Hour	*6.38*	*149.2*	*0.270*	*0.241*	*5.93*	*111.7*	*0.081*	*0.074*
36 Hour	*6.38*	*142.8*	*0.285*	*0.248*	5.93	92.4	0.043	0.036
42 Hour	*6.38*	*115.4*	*0.133*	*0.112*	5.93	76.1	0.036	0.031
48 Hour	6.38	117.4	0.074	0.063	5.93	89	0.014	0.013
60 Hour	6.38	18	0.012	0.009	5.93	3.3	0.001	0.001
72 Hour	6.38	1.6	0.000	0.000	5.93	0.1	0.000	0.000
		904		0.844		896		0.733

4.5.9 *70% Shockable: Strong Treatment Effects at 30 Hours with 30% Non-shockable: Small Treatment Effect at 18 Hours (Scenario 32.21.7)*

Table 4.5.9A: Summary Scenario Operating Characteristics

	**Shockable**	**Non-Shockable**
Pr(Select Acceptable Target Duration)	0.845	0.567
Pr(Positive Duration Response)	0.903	0.672
Pr(Futility)	0.002	0.009
Pr(Open 6 Hours)	0.225	0.486
Pr(Open 60 Hours)	0.420	0.061
Pr(Open 72 Hours)	0.075	0.004

Table 4.5.9B: Detailed Scenario Operating Characteristics

	**Shockable Rhythm**				**Non-Shockable Rhythm**			
	**Mean Weighted mRS**	**N**	**Pr (Target)**	**Pr (Target & Eff)**	**Mean Weighted mRS**	**N**	**Pr (Target)**	**Pr (Target & Eff)**
6 Hour	4.85	10.4	0.008	0.000	4.85	18.1	0.132	0.000
12 Hour	5.27	117.2	0.008	0.006	5.39	102.3	0.204	0.123
18 Hour	5.62	116.3	0.052	0.045	*5.93*	*90.6*	*0.307*	*0.239*
24 Hour	*5.95*	*210.6*	*0.104*	*0.093*	*5.93*	*101.3*	*0.183*	*0.158*
30 Hour	*6.38*	*218.6*	*0.298*	*0.275*	*5.93*	*67.5*	*0.077*	*0.067*
36 Hour	*6.38*	*215.4*	*0.299*	*0.278*	5.93	57.2	0.042	0.040
42 Hour	*6.38*	*169.2*	*0.144*	*0.130*	5.93	46.9	0.034	0.028
48 Hour	6.38	168	0.066	0.058	5.93	54.2	0.019	0.015
60 Hour	6.38	31.4	0.020	0.017	5.93	1.6	0.002	0.002
72 Hour	6.38	3.2	0.001	0.001	5.93	0.1	0.000	0.000
		1260		0.903		540		0.672

4.5.10 90% Shockable: Strong Treatment Effects at 30 Hours with 10% Non-shockable: Small Treatment Effect at 18 Hours (Scenario 32.21.9)

Table 4.5.10A: Summary Scenario Operating Characteristics

	**Shockable**	**Non-Shockable**
Pr(Select Acceptable Target Duration)	0.858	0.428
Pr(Positive Duration Response)	0.920	0.542
Pr(Futility)	0.004	0.000
Pr(Open 6 Hours)	0.198	0.242
Pr(Open 60 Hours)	0.474	0.040
Pr(Open 72 Hours)	0.089	0.002

Table 4.5.10B: Detailed Scenario Operating Characteristics

	**Shockable Rhythm**				**Non-Shockable Rhythm**			
	**Mean Weighted mRS**	**N**	**Pr (Target)**	**Pr (Target & Eff)**	**Mean Weighted mRS**	**N**	**Pr (Target)**	**Pr (Target & Eff)**
6 Hour	4.85	10.2	0.004	0.000	4.85	2.2	0.206	0.000
12 Hour	5.27	134.7	0.005	0.002	5.39	37.2	0.280	0.141
18 Hour	5.62	132.9	0.046	0.042	*5.93*	*29.8*	*0.212*	*0.144*
24 Hour	*5.95*	*258.8*	*0.089*	*0.081*	*5.93*	*34*	*0.154*	*0.132*
30 Hour	*6.38*	*294.1*	*0.328*	*0.302*	*5.93*	*23.7*	*0.062*	*0.052*
36 Hour	*6.38*	*292.2*	*0.318*	*0.300*	5.93	20.1	0.040	0.036
42 Hour	*6.38*	*225.1*	*0.123*	*0.114*	5.93	16.4	0.032	0.028
48 Hour	6.38	218.4	0.069	0.062	5.93	18.6	0.013	0.009
60 Hour	6.38	46.6	0.017	0.015	5.93	0.4	0.001	0.000
72 Hour	6.38	4.6	0.002	0.002	5.93	0	0	0.000
		1617.6		0.92		182.4		0.542

4.5.11 10% Shockable: Strong Treatment Effects at 30 Hours with 90% Non-shockable: U-Shaped Duration Response(Scenario 32.41.1)

Table 4.5.11A: Summary Scenario Operating Characteristics

	Shockable	Non-Shockable
Pr(Select Acceptable Target Duration)	0.541	0.920
Pr(Positive Duration Response)	0.486	0.799
Pr(Futility)	0.000	0.021
Pr(Open 6 Hours)	0.155	0.494
Pr(Open 60 Hours)	0.097	0.003
Pr(Open 72 Hours)	0.005	0.000

Table 4.5.11B: Detailed Scenario Operating Characteristics

	Shockable Rhythm				Non-Shockable Rhythm			
	**Mean Weighted mRS**	**N**	**Pr (Target)**	**Pr (Target & Eff)**	**Mean Weighted mRS**	**N**	**Pr (Target)**	**Pr (Target & Eff)**
6 Hour	4.85	1.4	0.133	0.000	4.85	31.6	0.024	0.000
12 Hour	5.27	32.7	0.160	0.041	5.62	238.6	0.056	0.033
18 Hour	5.62	27.2	0.142	0.074	*5.95*	*282.8*	*0.176*	*0.138*
24 Hour	*5.95*	*34.6*	*0.153*	*0.108*	*6.38*	*412.4*	*0.414*	*0.348*
30 Hour	*6.38*	*28.4*	*0.169*	*0.122*	*6.38*	*279.5*	*0.272*	*0.232*
36 Hour	*6.38*	*25.4*	*0.139*	*0.091*	*6.38*	*171*	*0.058*	*0.047*
42 Hour	*6.38*	*20.7*	*0.080*	*0.037*	5.95	88.6	0.001	0.001
48 Hour	6.38	21.7	0.024	0.012	5.62	102.1	0.000	0.000
60 Hour	6.38	1	0.001	0.001	4.85	0.1	0.000	0.000
72 Hour	6.38	0	0.000	0.000	4.25	0	0.000	0.000
		193		0.486		1606		0.799

4.5.12 30% Shockable: Strong Treatment Effects at 30 Hours with 70% Non-shockable: U-Shaped Duration Response (Scenario 32.41.3)

Table 4.5.12A: Summary Scenario Operating Characteristics

	**Shockable**	**Non-Shockable**
Pr(Select Acceptable Target Duration)	0.741	0.903
Pr(Positive Duration Response)	0.746	0.832
Pr(Futility)	0.002	0.015
Pr(Open 6 Hours)	0.286	0.507
Pr(Open 60 Hours)	0.255	0.006
Pr(Open 72 Hours)	0.024	0.000

Table 4.5.12B: Detailed Scenario Operating Characteristics

	**Shockable Rhythm**				**Non-Shockable Rhythm**			
	**Mean Weighted mRS**	**N**	**Pr (Target)**	**Pr (Target & Eff)**	**Mean Weighted mRS**	**N**	**Pr (Target)**	**Pr (Target & Eff)**
6 Hour	4.85	8.1	0.041	0.000	4.85	30.4	0.026	0.000
12 Hour	5.27	73.1	0.052	0.028	5.62	200.2	0.069	0.047
18 Hour	5.62	66.2	0.100	0.072	*5.95*	*218.5*	*0.196*	*0.158*
24 Hour	*5.95*	*96.7*	*0.141*	*0.117*	*6.38*	*300.9*	*0.395*	*0.350*
30 Hour	*6.38*	*83.4*	*0.244*	*0.204*	*6.38*	*207.7*	*0.249*	*0.222*
36 Hour	*6.38*	*78*	*0.234*	*0.191*	*6.38*	*136.2*	*0.063*	*0.054*
42 Hour	*6.38*	*64.8*	*0.122*	*0.091*	5.95	75.8	0.003	0.002
48 Hour	6.38	67.2	0.060	0.038	5.62	85.1	0.000	0.000
60 Hour	6.38	7.1	0.006	0.005	4.85	0.2	0.000	0.000
72 Hour	6.38	0.4	0.000	0.000	4.25	0	0.000	0.000
		545		0.746		1255		0.832

4.5.13 50% Shockable: Strong Treatment Effects at 30 Hours with 50% Non-shockable: U-Shaped Duration Response (Scenario 32.41.5)

Table 4.5.13A: Summary Scenario Operating Characteristics

	Shockable	Non-Shockable
Pr(Select Acceptable Target Duration)	0.811	0.841
Pr(Positive Duration Response)	0.855	0.814
Pr(Futility)	0.004	0.014
Pr(Open 6 Hours)	0.264	0.525
Pr(Open 60 Hours)	0.370	0.006
Pr(Open 72 Hours)	0.057	0.000

Table 4.5.13B: Detailed Scenario Operating Characteristics

	Shockable Rhythm				Non-Shockable Rhythm			
	**Mean Weighted mRS**	**N**	**Pr (Target)**	**Pr (Target & Eff)**	**Mean Weighted mRS**	**N**	**Pr (Target)**	**Pr (Target & Eff)**
6 Hour	4.85	10	0.016	0.000	4.85	25.7	0.041	0.000
12 Hour	5.27	99	0.021	0.013	5.62	156.8	0.116	0.080
18 Hour	5.62	94.7	0.068	0.056	*5.95*	*156*	*0.225*	*0.186*
24 Hour	*5.95*	*154.7*	*0.112*	*0.101*	*6.38*	*200.1*	*0.344*	*0.304*
30 Hour	*6.38*	*146.7*	*0.284*	*0.257*	*6.38*	*138.1*	*0.213*	*0.190*
36 Hour	*6.38*	*142.7*	*0.277*	*0.246*	*6.38*	*96.5*	*0.059*	*0.052*
42 Hour	*6.38*	*116.2*	*0.138*	*0.115*	5.95	58.8	0.002	0.002
48 Hour	6.38	117.5	0.063	0.048	5.62	65.8	0.000	0.000
60 Hour	6.38	18.9	0.020	0.018	4.85	0.1	0.000	0.000
72 Hour	6.38	1.7	0.001	0.001	4.25	0	0.000	0.000
		902		0.855		898		0.814

4.5.14 70% Shockable: Strong Treatment Effects at 30 Hours with 30% Non-shockable: U-Shaped Duration Response (Scenario 32.41.7)

Table 4.5.14A: Summary Scenario Operating Characteristics

	**Shockable**	**Non-Shockable**
Pr(Select Acceptable Target Duration)	0.837	0.747
Pr(Positive Duration Response)	0.915	0.756
Pr(Futility)	0.003	0.006
Pr(Open 6 Hours)	0.234	0.505
Pr(Open 60 Hours)	0.418	0.010
Pr(Open 72 Hours)	0.080	0.000

Table 4.5.14B: Detailed Scenario Operating Characteristics

	**Shockable Rhythm**				**Non-Shockable Rhythm**			
	**Mean Weighted mRS**	**N**	**Pr (Target)**	**Pr (Target & Eff)**	**Mean Weighted mRS**	**N**	**Pr (Target)**	**Pr (Target & Eff)**
6 Hour	4.85	10.8	0.008	0.000	4.85	16.7	0.085	0.000
12 Hour	5.27	118.2	0.007	0.005	5.62	101.7	0.162	0.107
18 Hour	5.62	116.3	0.060	0.052	*5.95*	*93.2*	*0.241*	*0.196*
24 Hour	*5.95*	*209.4*	*0.094*	*0.084*	*6.38*	*112.7*	*0.289*	*0.257*
30 Hour	*6.38*	*217.7*	*0.312*	*0.289*	*6.38*	*77.2*	*0.161*	*0.140*
36 Hour	*6.38*	*213.7*	*0.299*	*0.281*	*6.38*	*58*	*0.056*	*0.050*
42 Hour	*6.38*	*169.1*	*0.132*	*0.122*	5.95	38.5	0.006	0.006
48 Hour	6.38	168	0.070	0.065	5.62	43.1	0.000	0.000
60 Hour	6.38	32.2	0.019	0.017	4.85	0.2	0.000	0.000
72 Hour	6.38	3.5	0.000	0.000	4.25	0	0.000	0.000
		1259		0.915		541		0.756

4.5.15 90% Shockable: Strong Treatment Effects at 30 Hours with 10% Non-shockable: U-Shaped Duration Response (Scenario 32.41.9)

Table 4.5.15A: Summary Scenario Operating Characteristics

	**Shockable**	**Non-Shockable**
Pr(Select Acceptable Target Duration)	0.866	0.519
Pr(Positive Duration Response)	0.924	0.548
Pr(Futility)	0.003	0.000
Pr(Open 6 Hours)	0.196	0.245
Pr(Open 60 Hours)	0.460	0.015
Pr(Open 72 Hours)	0.091	0.000

Table 4.5.15B: Detailed Scenario Operating Characteristics

	**Shockable Rhythm**				**Non-Shockable Rhythm**			
	**Mean Weighted mRS**	**N**	**Pr (Target)**	**Pr (Target & Eff)**	**Mean Weighted mRS**	**N**	**Pr (Target)**	**Pr (Target & Eff)**
6 Hour	4.85	9.7	0.006	0.000	4.85	2.1	0.201	0.000
12 Hour	5.27	132.7	0.002	0.001	5.62	37.5	0.267	0.137
18 Hour	5.62	134.0	0.042	0.034	*5.95*	*30.6*	*0.192*	*0.126*
24 Hour	*5.95*	*266.1*	*0.093*	*0.085*	*6.38*	*35.6*	*0.183*	*0.152*
30 Hour	*6.38*	*299.9*	*0.333*	*0.314*	*6.38*	*24.9*	*0.100*	*0.084*
36 Hour	*6.38*	*290.9*	*0.323*	*0.306*	*6.38*	*20.1*	*0.044*	*0.039*
42 Hour	*6.38*	*219.7*	*0.117*	*0.107*	5.95	14.8	0.010	0.008
48 Hour	6.38	214.1	0.065	0.058	5.62	16.6	0.002	0.002
60 Hour	6.38	45.0	0.017	0.017	4.850	0.1	0	0
72 Hour	6.38	5.5	0.002	0.002	4.250	0.0	0	0
		1617.6		0.924		182.3		0.548

4.5.16 10% Shockable: Strong Treatment Effects at 30 Hours with 90% Non-shockable: Increasing Duration Response (Scenario 32.42.1)

Table 4.5.16A: Summary Scenario Operating Characteristics

	**Shockable**	**Non-Shockable**
Pr(Select Acceptable Target Duration)	0.534	0.598
Pr(Positive Duration Response)	0.719	0.901
Pr(Futility)	0.000	0.020
Pr(Open 6 Hours)	0.159	0.273
Pr(Open 60 Hours)	0.112	0.824
Pr(Open 72 Hours)	0.006	0.634

Table 4.5.16B: Detailed Scenario Operating Characteristics

	**Shockable Rhythm**				**Non-Shockable Rhythm**			
	**Mean Weighted mRS**	**N**	**Pr (Target)**	**Pr (Target & Eff)**	**Mean Weighted mRS**	**N**	**Pr (Target)**	**Pr (Target & Eff)**
6 Hour	4.85	1.4	0.119	0.000	4.85	21.3	0.019	0.000
12 Hour	5.27	31.9	0.170	0.108	5.015	159.7	0.016	0.006
18 Hour	5.62	26.4	0.138	0.108	5.18	121.7	0.039	0.032
24 Hour	*5.95*	*34.2*	*0.148*	*0.125*	5.345	192.7	0.039	0.032
30 Hour	*6.38*	*27.5*	*0.166*	*0.145*	5.51	150.2	0.039	0.033
36 Hour	*6.38*	*25.1*	*0.126*	*0.114*	5.675	175.9	0.056	0.048
42 Hour	*6.38*	*21.1*	*0.094*	*0.084*	5.84	202.5	0.066	0.056
48 Hour	6.38	22.1	0.035	0.031	6.14	284.5	0.128	0.109
60 Hour	6.38	1.2	0.004	0.004	*6.44*	*167.8*	*0.159*	*0.151*
72 Hour	6.38	0.0	0.000	0.000	*7.04*	*132.7*	*0.439*	*0.435*
		190.9		0.719		1609.0		0.902

4.5.17 30% Shockable: Strong Treatment Effects at 30 Hours with 70% Non-shockable: Increasing Duration Response (Scenario 32.42.3)

Table 4.5.17A: Summary Scenario Operating Characteristics

	**Shockable**	**Non-Shockable**
Pr(Select Acceptable Target Duration)	0.732	0.479
Pr(Positive Duration Response)	0.872	0.887
Pr(Futility)	0.001	0.015
Pr(Open 6 Hours)	0.285	0.296
Pr(Open 60 Hours)	0.254	0.766
Pr(Open 72 Hours)	0.025	0.517

Table 4.5.17B: Detailed Scenario Operating Characteristics

	**Shockable Rhythm**				**Non-Shockable Rhythm**			
	**Mean Weighted mRS**	**N**	**Pr (Target)**	**Pr (Target & Eff)**	**Mean Weighted mRS**	**N**	**Pr (Target)**	**Pr (Target & Eff)**
6 Hour	4.85	8.1	0.042	0.000	4.85	20.4	0.030	0.000
12 Hour	5.27	72.8	0.052	0.040	5.015	139.6	0.028	0.017
18 Hour	5.62	66.1	0.100	0.086	5.18	107.7	0.046	0.033
24 Hour	*5.95*	*96.6*	*0.135*	*0.120*	5.345	159.9	0.052	0.046
30 Hour	*6.38*	*84.3*	*0.262*	*0.244*	5.51	126	0.052	0.047
36 Hour	*6.38*	*79.2*	*0.225*	*0.207*	5.675	143.4	0.058	0.051
42 Hour	*6.38*	*64.6*	*0.110*	*0.103*	5.84	161	0.085	0.074
48 Hour	6.38	67.3	0.066	0.064	6.14	219	0.170	0.150
60 Hour	6.38	7.1	0.009	0.008	*6.44*	*109.9*	*0.172*	*0.165*
72 Hour	6.38	0.5	0.000	0.000	*7.04*	*66.5*	*0.307*	*0.304*
		547		0.872		1253		0.887

4.5.18 50% Shockable: Strong Treatment Effects at 30 Hours with 50% Non-shockable: Increasing Duration Response (Scenario 32.42.5)

Table 4.5.18A: Summary Scenario Operating Characteristics

	Shockable	Non-Shockable
Pr(Select Acceptable Target Duration)	0.817	0.276
Pr(Positive Duration Response)	0.908	0.828
Pr(Futility)	0.004	0.020
Pr(Open 6 Hours)	0.281	0.352
Pr(Open 60 Hours)	0.336	0.580
Pr(Open 72 Hours)	0.050	0.306

Table 4.5.18B: Detailed Scenario Operating Characteristics

	Shockable Rhythm				Non-Shockable Rhythm			
	Mean Weighted mRS	N	Pr (Target)	Pr (Target & Eff)	Mean Weighted mRS	N	Pr (Target)	Pr (Target & Eff)
6 Hour	4.85	11.4	0.022	0.000	4.85	20.1	0.052	0.000
12 Hour	5.27	100.7	0.018	0.013	5.015	119.1	0.078	0.050
18 Hour	5.62	96	0.065	0.054	5.18	92.4	0.074	0.058
24 Hour	*5.95*	*156.7*	*0.132*	*0.122*	5.345	127.7	0.062	0.053
30 Hour	*6.38*	*149*	*0.288*	*0.266*	5.51	100.2	0.071	0.065
36 Hour	*6.38*	*142.7*	*0.271*	*0.258*	5.675	108.4	0.085	0.079
42 Hour	*6.38*	*114.1*	*0.126*	*0.120*	5.84	114	0.110	0.099
48 Hour	6.38	115.2	0.064	0.060	6.14	144.6	0.192	0.164
60 Hour	6.38	16.4	0.014	0.014	*6.44*	*49.7*	*0.165*	*0.150*
72 Hour	6.38	1.3	0.000	0.000	*7.04*	*20.2*	*0.111*	*0.110*
		904		0.908		897		0.828

4.5.19 70% Shockable: Strong Treatment Effects at 30 Hours with 30% Non-shockable: Increasing Duration Response (Scenario 32.42.7)

Table 4.5.19A: Summary Scenario Operating Characteristics

	**Shockable**	**Non-Shockable**
Pr(Select Acceptable Target Duration)	0.852	0.114
Pr(Positive Duration Response)	0.944	0.758
Pr(Futility)	0.004	0.006
Pr(Open 6 Hours)	0.226	0.355
Pr(Open 60 Hours)	0.413	0.396
Pr(Open 72 Hours)	0.082	0.146

Table 4.5.19B: Detailed Scenario Operating Characteristics

	**Shockable Rhythm**				**Non-Shockable Rhythm**			
	**Mean Weighted mRS**	**N**	**Pr (Target)**	**Pr (Target & Eff)**	**Mean Weighted mRS**	**N**	**Pr (Target)**	**Pr (Target & Eff)**
6 Hour	4.85	10.3	0.004	0.000	4.85	12.6	0.110	0.000
12 Hour	5.27	117.1	0.010	0.008	5.015	82.6	0.135	0.086
18 Hour	5.62	116.4	0.047	0.041	5.18	64.8	0.101	0.081
24 Hour	*5.95*	*211.4*	*0.111*	*0.106*	5.345	84.5	0.081	0.072
30 Hour	*6.38*	*220.7*	*0.309*	*0.297*	5.51	65	0.084	0.076
36 Hour	*6.38*	*213.9*	*0.293*	*0.279*	5.675	65.9	0.084	0.076
42 Hour	*6.38*	*167.9*	*0.139*	*0.132*	5.84	65	0.121	0.110
48 Hour	6.38	166.7	0.068	0.063	6.14	78.6	0.171	0.149
60 Hour	6.38	31.1	0.018	0.016	*6.44*	*17.3*	*0.086*	*0.080*
72 Hour	6.38	3.6	0.001	0.001	*7.04*	*4.6*	*0.028*	*0.028*
		1259		0.944		541		0.758

4.5.20 90% Shockable: Strong Treatment Effects at 30 Hours with 10% Non-shockable: Increasing Duration Response (Scenario 32.42.9)

Table 4.5.20A: Summary Scenario Operating Characteristics

	**Shockable**	**Non-Shockable**
Pr(Select Acceptable Target Duration)	0.860	0.011
Pr(Positive Duration Response)	0.940	0.575
Pr(Futility)	0.005	0.000
Pr(Open 6 Hours)	0.188	0.181
Pr(Open 60 Hours)	0.478	0.133
Pr(Open 72 Hours)	0.105	0.017

Table 4.5.20B: Detailed Scenario Operating Characteristics

	**Shockable Rhythm**				**Non-Shockable Rhythm**			
	**Mean Weighted mRS**	**N**	**Pr (Target)**	**Pr (Target & Eff)**	**Mean Weighted mRS**	**N**	**Pr (Target)**	**Pr (Target & Eff)**
6 Hour	4.85	9.5	0.004	0.000	4.85	1.6	0.219	0.000
12 Hour	5.27	130.9	0.003	0.002	5.015	34.3	0.245	0.133
18 Hour	5.62	131.2	0.042	0.036	5.18	26.2	0.126	0.091
24 Hour	*5.95*	*261.9*	*0.091*	*0.083*	5.345	31.3	0.093	0.080
30 Hour	*6.38*	*295.1*	*0.325*	*0.310*	5.51	23.3	0.072	0.064
36 Hour	*6.38*	*291.9*	*0.326*	*0.308*	5.675	22.1	0.080	0.071
42 Hour	*6.38*	*223.4*	*0.118*	*0.113*	5.84	19.7	0.089	0.078
48 Hour	6.38	218.7	0.066	0.064	6.14	22.4	0.065	0.050
60 Hour	6.38	48.5	0.024	0.024	*6.44*	*1.7*	*0.010*	*0.008*
72 Hour	6.38	6.1	0.001	0.001	*7.04*	*0.2*	*0.001*	*0.001*
		1617		0.940		183		0.575

4.5.21 10% Shockable: Strong Treatment Effects at 30 Hours with 90% Non-shockable: Large Treatment Effect at 48 (Scenario 32.53.1)

Table 4.5.21A: Summary Scenario Operating Characteristics

	**Shockable**	**Non-Shockable**
Pr(Select Acceptable Target Duration)	0.535	0.788
Pr(Positive Duration Response)	0.774	0.996
Pr(Futility)	0.000	0.000
Pr(Open 6 Hours)	0.160	0.082
Pr(Open 60 Hours)	0.100	0.974
Pr(Open 72 Hours)	0.007	0.821

Table 4.5.21B: Detailed Scenario Operating Characteristics

	**Shockable Rhythm**				**Non-Shockable Rhythm**			
	**Mean Weighted mRS**	**N**	**Pr (Target)**	**Pr (Target & Eff)**	**Mean Weighted mRS**	**N**	**Pr (Target)**	**Pr (Target & Eff)**
6 Hour	4.85	1.3	0.134	0.000	4.85	3.6	0.001	0.000
12 Hour	5.27	31.4	0.165	0.126	5.20	94.1	0.000	0.000
18 Hour	5.62	25.5	0.131	0.115	5.51	65.4	0.028	0.028
24 Hour	*5.95*	*32.7*	*0.147*	*0.132*	5.77	145.5	0.035	0.035
30 Hour	*6.38*	*25.4*	*0.170*	*0.158*	6.08	120.5	0.042	0.042
36 Hour	*6.38*	*23.2*	*0.134*	*0.126*	6.24	172.0	0.040	0.039
42 Hour	*6.38*	*19.2*	*0.084*	*0.082*	*6.59*	*251.2*	*0.082*	*0.082*
48 Hour	6.38	20.8	0.034	0.032	*6.90*	*412.1*	*0.359*	*0.359*
60 Hour	6.38	0.9	0.002	0.002	*6.90*	*260.9*	*0.347*	*0.345*
72 Hour	6.38	0.0	0.000	0.000	6.90	94.1	0.066	0.066
		180.4		0.773		1619.4		0.996

4.5.22 30% Shockable: Strong Treatment Effects at 30 Hours with 70% Non-shockable: Large Treatment Effect at 48 (Scenario 32.53.3)

Table 4.5.22A: Summary Scenario Operating Characteristics

	**Shockable**	**Non-Shockable**
Pr(Select Acceptable Target Duration)	0.734	0.813
Pr(Positive Duration Response)	0.926	0.993
Pr(Futility)	0.001	0.002
Pr(Open 6 Hours)	0.280	0.098
Pr(Open 60 Hours)	0.271	0.957
Pr(Open 72 Hours)	0.031	0.739

Table 4.5.22B: Detailed Scenario Operating Characteristics

	**Shockable Rhythm**				**Non-Shockable Rhythm**			
	**Mean Weighted mRS**	**N**	**Pr (Target)**	**Pr (Target & Eff)**	**Mean Weighted mRS**	**N**	**Pr (Target)**	**Pr (Target & Eff)**
6 Hour	4.85	7.9	0.042	0.000	4.85	3.6	0.002	0.000
12 Hour	5.27	72.2	0.050	0.042	5.2	83.3	0.002	0.001
18 Hour	5.62	64.1	0.099	0.095	5.51	59.4	0.017	0.017
24 Hour	*5.95*	*94.5*	*0.141*	*0.137*	5.77	122.7	0.030	0.030
30 Hour	*6.38*	*82.3*	*0.233*	*0.230*	6.08	104.9	0.037	0.036
36 Hour	*6.38*	*78.3*	*0.230*	*0.224*	6.24	145.5	0.054	0.054
42 Hour	*6.38*	*65*	*0.130*	*0.126*	*6.59*	*201.7*	*0.100*	*0.099*
48 Hour	6.38	67.9	0.066	0.064	*6.90*	*304.6*	*0.389*	*0.388*
60 Hour	6.38	7.7	0.009	0.009	*6.90*	*176.1*	*0.324*	*0.323*
72 Hour	6.38	0.5	0.000	0.000	6.90	57.8	0.045	0.045
		541		0.926		1260		0.993

4.5.23 50% Shockable: Strong Treatment Effects at 30 Hours with 50% Non-shockable: Large Treatment Effect at 48 (Scenario 32.53.5)

Table 4.5.23A: Summary Scenario Operating Characteristics

	**Shockable**	**Non-Shockable**
Pr(Select Acceptable Target Duration)	0.811	0.783
Pr(Positive Duration Response)	0.961	0.978
Pr(Futility)	0.004	0.001
Pr(Open 6 Hours)	0.270	0.150
Pr(Open 60 Hours)	0.366	0.890
Pr(Open 72 Hours)	0.058	0.601

Table 4.5.23B: Detailed Scenario Operating Characteristics

	**Shockable Rhythm**				**Non-Shockable Rhythm**			
	**Mean Weighted mRS**	**N**	**Pr (Target)**	**Pr (Target & Eff)**	**Mean Weighted mRS**	**N**	**Pr (Target)**	**Pr (Target & Eff)**
6 Hour	4.85	10.7	0.015	0.000	4.85	4.8	0.004	0.000
12 Hour	5.27	99	0.018	0.017	5.2	71.5	0.002	0.002
18 Hour	5.62	93.7	0.068	0.066	5.51	54.5	0.027	0.026
24 Hour	*5.95*	*153.4*	*0.112*	*0.110*	5.77	101.8	0.033	0.032
30 Hour	*6.38*	*147.5*	*0.283*	*0.276*	6.08	89	0.057	0.054
36 Hour	*6.38*	*141.9*	*0.272*	*0.265*	6.24	114.4	0.068	0.068
42 Hour	*6.38*	*115.4*	*0.144*	*0.142*	*6.59*	*143.8*	*0.133*	*0.130*
48 Hour	6.38	117.5	0.072	0.070	*6.90*	*197.2*	*0.390*	*0.384*
60 Hour	6.38	18.7	0.014	0.014	*6.90*	*96.3*	*0.260*	*0.256*
72 Hour	6.38	1.6	0.000	0.000	6.90	27.3	0.026	0.026
		899		0.961		901		0.978

4.5.24 70% Shockable: Strong Treatment Effects at 30 Hours with 30% Non-shockable: Large Treatment Effect at 48 (Scenario 32.53.7)

Table 4.5.24A: Summary Scenario Operating Characteristics

	Shockable	Non-Shockable
Pr(Select Acceptable Target Duration)	0.843	0.661
Pr(Positive Duration Response)	0.973	0.932
Pr(Futility)	0.006	0.000
Pr(Open 6 Hours)	0.239	0.191
Pr(Open 60 Hours)	0.408	0.714
Pr(Open 72 Hours)	0.079	0.326

Table 4.5.24B: Detailed Scenario Operating Characteristics

	Shockable Rhythm				Non-Shockable Rhythm			
	**Mean Weighted mRS**	**N**	**Pr (Target)**	**Pr (Target & Eff)**	**Mean Weighted mRS**	**N**	**Pr (Target)**	**Pr (Target & Eff)**
6 Hour	4.85	10.7	0.005	0.000	4.85	4.7	0.019	0.000
12 Hour	5.27	118.6	0.008	0.006	5.2	59	0.023	0.018
18 Hour	5.62	118.4	0.054	0.052	5.51	48	0.042	0.038
24 Hour	*5.95*	*212.6*	*0.117*	*0.116*	5.77	75.3	0.067	0.062
30 Hour	*6.38*	*217*	*0.301*	*0.295*	6.08	63.3	0.078	0.074
36 Hour	*6.38*	*211.8*	*0.299*	*0.293*	6.24	71.7	0.104	0.097
42 Hour	*6.38*	*167.2*	*0.126*	*0.122*	*6.59*	*79.6*	*0.173*	*0.169*
48 Hour	6.38	166.6	0.068	0.067	*6.90*	*99*	*0.352*	*0.334*
60 Hour	6.38	31.9	0.021	0.021	*6.90*	*34.1*	*0.136*	*0.132*
72 Hour	6.38	3.4	0.001	0.001	6.90	7	0.007	0.007
		1258		0.973		541		0.932

4.5.25 90% Shockable: Strong Treatment Effects at 30 Hours with 10% Non-shockable: Large Treatment Effect at 48 (Scenario 32.53.9)

Table 4.5.25A: Summary Scenario Operating Characteristics

	**Shockable**	**Non-Shockable**
Pr(Select Acceptable Target Duration)	0.879	0.310
Pr(Positive Duration Response)	0.951	0.742
Pr(Futility)	0.003	0.000
Pr(Open 6 Hours)	0.201	0.130
Pr(Open 60 Hours)	0.446	0.258
Pr(Open 72 Hours)	0.094	0.038

Table 4.5.25B: Detailed Scenario Operating Characteristics

	**Shockable Rhythm**				**Non-Shockable Rhythm**			
	**Mean Weighted mRS**	**N**	**Pr (Target)**	**Pr (Target & Eff)**	**Mean Weighted mRS**	**N**	**Pr (Target)**	**Pr (Target & Eff)**
6 Hour	4.85	10.0	0.005	0.000	4.85	1.0	0.112	0.000
12 Hour	5.27	132.0	0.003	0.002	5.20	28.7	0.129	0.085
18 Hour	5.62	134.4	0.037	0.036	5.51	23.2	0.110	0.084
24 Hour	*5.95*	*266.8*	*0.100*	*0.094*	5.77	30.8	0.118	0.104
30 Hour	*6.38*	*299.5*	*0.328*	*0.315*	6.08	24.3	0.110	0.101
36 Hour	*6.38*	*292.8*	*0.320*	*0.308*	6.24	23.9	0.111	0.100
42 Hour	*6.38*	*219.8*	*0.131*	*0.123*	*6.59*	*22.1*	*0.165*	*0.148*
48 Hour	6.38	213.5	0.056	0.054	*6.90*	*24.7*	*0.128*	*0.106*
60 Hour	6.38	44.1	0.0	0.018	*6.90*	*2.9*	*0.017*	*0.015*
72 Hour	6.38	5.4	0.0	0.000	6.90	0.2	0.000	0.000
		1618.3		0.950		181.8		0.743

4.6 Half-Null Scenarios

In this section we present scenarios in which one of the two rhythm types has no benefit, but the other rhythm type does have a benefit. In each of these cases we assume a 50% prevalence rate for each rhythm type. See Section 4.5 for a combining of the strong effect at 30-hours with a null scenario and varying rates in each rhythm type. We pair the null scenario (for non-shockable) with a scenario of positive effect in the shockable rhythm type. We summarize the operating characteristics for each run in Table 4.6 and present a summary of each scenario run in the following subsections.

An important note is that we do not view these scenarios as being a part of the null hypothesis for type I error control. For the type I error control we assume that each rhythm type must have no benefit of increasing durations. We say this because any analysis combining data across rhythm types would certainly claim a benefit on the population as a whole. The operating characteristics demonstrate much better behavior than a pooling across rhythm types, but does not claim control of type I error for each subpopulation.

Table 4.6: The summary operating characteristics for various combinations of different treatment effects and a null effect. The # column refers to the labeling of each scenario for the back-up software output. The Name column refers to the scenario name presented throughout Section 4. The operating characteristics presented for each rhythm type are N: the mean sample size; and Positive: The proportion of trials with a statistically significant positive trend is also reported.

#	Name	N	Positive	#	Name	N	Positive
**21**	**Small 18**	933.5	.347	**1**	**Null**	865.2	.110
**32**	**Strong 30**	941.7	.748	**1**	**Null**	858.1	.259
**41**	**U-Shaped**	935.8	.476	**1**	**Null**	862.6	.134
**42**	**Increasing**	936.5	.602	**1**	**Null**	862.1	.254
**53**	**Large 48**	941.5	.919	**1**	**Null**	858.5	.359

4.6.1 Shockable: Small Treatment Effects at 18 Hours with Non-shockable: Null Treatment Effect (Scenario 21.1.5)

Table 4.6.1A: Summary Scenario Operating Characteristics

	Shockable	Non-Shockable
Pr(Select Acceptable Target Duration)	0.671	0.887
Pr(Positive Duration Response)	0.347	0.110
Pr(Futility)	0.026	0.187
Pr(Open 6 Hours)	0.531	0.784
Pr(Open 60 Hours)	0.078	0.044
Pr(Open 72 Hours)	0.009	0.002

Table 4.6.1B: Detailed Scenario Operating Characteristics

	Shockable Rhythm				Non-Shockable Rhythm			
	Mean Weighted mRS	N	Pr (Target)	Pr (Target & Eff)	Mean Weighted mRS	N	Pr (Target)	Pr (Target & Eff)
6 Hour	4.85	30.2	0.085	0.000	*4.85*	*73.8*	*0.435*	*0.000*
12 Hour	5.39	164.5	0.146	0.024	*4.85*	*196.0*	*0.364*	*0.035*
18 Hour	*5.93*	*166.0*	*0.370*	*0.141*	*4.85*	*131.2*	*0.088*	*0.029*
24 Hour	*5.93*	*180.3*	*0.221*	*0.094*	4.85	139.5	0.045	0.016
30 Hour	*5.93*	*117.2*	*0.080*	*0.041*	4.85	91.3	0.022	0.012
36 Hour	5.93	97.3	0.042	0.020	4.85	80.1	0.019	0.008
42 Hour	5.93	80.8	0.032	0.017	4.85	67.5	0.017	0.005
48 Hour	5.93	93.0	0.020	0.008	4.85	83.7	0.010	0.004
60 Hour	5.93	3.9	0.004	0.002	4.85	2.1	0.000	0.000
72 Hour	5.93	0.3	0.000	0.000	4.85	0.1	0.000	0.000
		933.5		0.347		865.2		0.110

4.6.2 Shockable: Strong Treatment Effects at 30 Hours with Non-shockable: Null Treatment Effect (Scenario 32.1.5)

Table 4.6.2A: Summary Scenario Operating Characteristics

	**Shockable**	**Non-Shockable**
Pr(Select Acceptable Target Duration)	0.824	0.889
Pr(Positive Duration Response)	0.748	0.259
Pr(Futility)	0.003	0.191
Pr(Open 6 Hours)	0.264	0.774
Pr(Open 60 Hours)	0.349	0.043
Pr(Open 72 Hours)	0.054	0.004

Table 4.6.2B: Detailed Scenario Operating Characteristics

	**Shockable Rhythm**				**Non-Shockable Rhythm**			
	**Mean Weighted mRS**	**N**	**Pr (Target)**	**Pr (Target & Eff)**	**Mean Weighted mRS**	**N**	**Pr (Target)**	**Pr (Target & Eff)**
6 Hour	4.85	10.6	0.016	0.000	*4.85*	*72.6*	*0.423*	*0.000*
12 Hour	5.27	100.5	0.020	0.006	*4.85*	*194.4*	*0.390*	*0.136*
18 Hour	5.62	97.1	0.062	0.036	*4.85*	*129.6*	*0.076*	*0.041*
24 Hour	*5.95*	*160.1*	*0.112*	*0.080*	4.85	138.6	0.054	0.039
30 Hour	*6.38*	*157.5*	*0.313*	*0.241*	4.85	90.7	0.020	0.014
36 Hour	*6.38*	*152.4*	*0.282*	*0.229*	4.85	79.6	0.015	0.011
42 Hour	*6.38*	*122.2*	*0.117*	*0.094*	4.85	67	0.012	0.010
48 Hour	6.38	120.8	0.064	0.050	4.85	83.4	0.009	0.006
60 Hour	6.38	18.9	0.013	0.010	4.85	2	0.002	0.001
72 Hour	6.38	1.6	0.002	0.001	4.85	0.1	0.000	0.000
		942		0.748		858		0.259

4.6.3 Shockable: U-Shaped Duration Response Non-shockable: Null Treatment Effect (Scenario 41.1.5)

Table 4.6.3A: Summary Scenario Operating Characteristics

	**Shockable**	**Non-Shockable**
Pr(Select Acceptable Target Duration)	0.864	0.888
Pr(Positive Duration Response)	0.476	0.134
Pr(Futility)	0.018	0.202
Pr(Open 6 Hours)	0.511	0.790
Pr(Open 60 Hours)	0.008	0.042
Pr(Open 72 Hours)	0.000	0.003

Table 4.6.3B: Detailed Scenario Operating Characteristics

	**Shockable Rhythm**				**Non-Shockable Rhythm**			
	**Mean Weighted mRS**	**N**	**Pr (Target)**	**Pr (Target & Eff)**	**Mean Weighted mRS**	**N**	**Pr (Target)**	**Pr (Target & Eff)**
6 Hour	4.85	25.9	0.042	0.000	*4.85*	*75*	*0.437*	*0.000*
12 Hour	5.62	157.2	0.089	0.024	*4.85*	*195.4*	*0.369*	*0.054*
18 Hour	*5.95*	*161*	*0.217*	*0.104*	*4.85*	*130.3*	*0.082*	*0.032*
24 Hour	*6.38*	*211.5*	*0.374*	*0.196*	4.85	139.1	0.047	0.022
30 Hour	*6.38*	*148.4*	*0.222*	*0.123*	4.85	91.1	0.026	0.013
36 Hour	*6.38*	*102.7*	*0.051*	*0.027*	4.85	79.4	0.015	0.007
42 Hour	5.95	61.8	0.003	0.002	4.85	67	0.015	0.005
48 Hour	5.62	67.2	0.001	0.000	4.85	83.4	0.009	0.000
60 Hour	4.85	0.2	0.000	0.000	4.85	1.8	0.000	0.000
72 Hour	4.25	0	0.000	0.000	4.85	0.1	0.000	0.000
		936		0.476		862		0.134

4.6.4 Shockable: Increasing Duration Response Non-shockable: Null Treatment Effect (Scenario 42.1.5)

Table 4.6.4A: Summary Scenario Operating Characteristics

	**Shockable**	**Non-Shockable**
Pr(Select Acceptable Target Duration)	0.302	0.897
Pr(Positive Duration Response)	0.602	0.254
Pr(Futility)	0.018	0.185
Pr(Open 6 Hours)	0.347	0.770
Pr(Open 60 Hours)	0.604	0.027
Pr(Open 72 Hours)	0.329	0.000

Table 4.6.4B: Detailed Scenario Operating Characteristics

	**Shockable Rhythm**				**Non-Shockable Rhythm**			
	**Mean Weighted mRS**	**N**	**Pr (Target)**	**Pr (Target & Eff)**	**Mean Weighted mRS**	**N**	**Pr (Target)**	**Pr (Target & Eff)**
6 Hour	4.85	20.1	0.054	0.000	*4.85*	*72.4*	*0.426*	*0.000*
12 Hour	5.015	120.9	0.070	0.008	*4.85*	*196.5*	*0.388*	*0.136*
18 Hour	5.18	94.9	0.062	0.028	*4.85*	*130.9*	*0.083*	*0.045*
24 Hour	5.345	130.4	0.061	0.024	4.85	139.2	0.046	0.032
30 Hour	5.51	104.1	0.076	0.047	4.85	91.4	0.022	0.016
36 Hour	5.675	112.7	0.076	0.049	4.85	79.9	0.011	0.008
42 Hour	5.84	118.7	0.110	0.065	4.85	67.1	0.016	0.012
48 Hour	6.14	150.6	0.190	0.114	4.85	83.3	0.006	0.005
60 Hour	*6.44*	*57.4*	*0.162*	*0.132*	4.85	1.3	0.000	0.000
72 Hour	*7.04*	*26.8*	*0.140*	*0.136*	4.85	0	0.000	0.000
		937		0.602		862		0.254

4.6.5 Shockable: Large Treatment Effect at 48 Hours Non-shockable: Null Treatment Effect (Scenario 53.1.5)

Table 4.6.5A: Summary Scenario Operating Characteristics

	**Shockable**	**Non-Shockable**
Pr(Select Acceptable Target Duration)	0.792	0.898
Pr(Positive Duration Response)	0.919	0.359
Pr(Futility)	0.001	0.182
Pr(Open 6 Hours)	0.158	0.784
Pr(Open 60 Hours)	0.901	0.034
Pr(Open 72 Hours)	0.600	0.002

Table 4.6.5B: Detailed Scenario Operating Characteristics

	**Shockable Rhythm**				**Non-Shockable Rhythm**			
	**Mean Weighted mRS**	**N**	**Pr (Target)**	**Pr (Target & Eff)**	**Mean Weighted mRS**	**N**	**Pr (Target)**	**Pr (Target & Eff)**
6 Hour	4.85	5.3	0.003	0.000	*4.85*	*73.4*	*0.445*	*0.000*
12 Hour	5.2	74.2	0.004	0.002	*4.85*	*194.8*	*0.362*	*0.202*
18 Hour	5.51	58.1	0.017	0.014	*4.85*	*130.8*	*0.091*	*0.070*
24 Hour	5.77	105.1	0.039	0.036	4.85	138.8	0.041	0.032
30 Hour	6.08	92.5	0.042	0.035	4.85	90.8	0.025	0.021
36 Hour	6.24	119.2	0.068	0.062	4.85	79	0.013	0.012
42 Hour	*6.59*	*150.4*	*0.140*	*0.128*	4.85	66.3	0.014	0.013
48 Hour	*6.9*	*203.7*	*0.387*	*0.354*	4.85	82.8	0.008	0.006
60 Hour	*6.9*	*102.3*	*0.265*	*0.256*	4.85	1.7	0.002	0.002
72 Hour	6.9	30.7	0.034	0.034	4.85	0.1	0.000	0.000
		942		0.919		859		0.359

4.7 Sample Size Justification

In order to determine the maximum sample size for this trial, we considered power for various scenarios across a range of total sample sizes from 1500 to 2300 patients. We considered power for selecting a clinically acceptable duration and for determining if cooling is effective. We primarily examined three reference scenarios where the maximum treatment effect was the same in each scenario, but the treatment effect plateaus in a different location.

This section reviews each of these simulated scenarios through the sample size range. At the end of this section, we then graphically illustrate the effect of varying sample size from 1,500 to 2,300 on the probability of identifying a positive duration response and the probability of detecting the best duration in simulations of these 3 reference scenarios, and two illustrative scenarios of affect sizes smaller than those the trial is designed to detect.

In Scenario 1 below, the target duration is the 18-hour duration arm. This corresponds to the scenario described in section 4.2.7 above. In Scenario 2 below, the target duration is the 30-hour duration arm. This corresponds to the scenario described in section 4.2.2 above. In Scenario 3, the target duration is the 48-hour duration arm, corresponding to section 4.4.5. The scenario in terms of the mean weighted mRS across each arm is shown with the clinically acceptable durations in red italics. Results for the total sample size of 1800 patients are in bold.

**Scenario 1 (Both Rhythm Types)**		**Total Sample Size**	**Power**	
			**Clinically Acceptable Duration**	**Positive Duration-Response**
Arm	Mean Weighted mRS	1500	0.762	0.694
		1600	0.774	0.709
		1700	0.780	0.741
6 Hour	4.85	**1800**	**0.797**	**0.776**
12 Hour	5.615	1900	0.800	0.798
18 Hour	*6.38*	2000	0.815	0.808
24 Hour	*6.38*	2100	0.820	0.819
30 Hour	*6.38*	2200	0.826	0.838
36 Hour	6.38	2300	0.839	0.856
42 Hour	6.38			
48 Hour	6.38			
60 Hour	6.38			
72 Hour	6.38			

Scenario 2 (Both Rhythm Types)

Arm	Mean Weighted mRS
6 Hour	4.85
12 Hour	5.27
18 Hour	5.62
24 Hour	*5.95*
30 Hour	*6.38*
36 Hour	*6.38*
42 Hour	*6.38*
48 Hour	6.38
60 Hour	6.38
72 Hour	6.38

Total Sample Size	Power: Clinically Acceptable Duration	Power: Positive Duration-Response
1500	0.750	0.894
1600	0.768	0.909
1700	0.771	0.924
**1800**	**0.781**	**0.937**
1900	0.785	0.946
2000	0.793	0.952
2100	0.803	0.952
2200	0.814	0.963
2300	0.818	0.970

Scenario 3 (Both Rhythm Types)

Arm	Mean Weighted mRS
6 Hour	4.85
12 Hour	5.06
18 Hour	5.19
24 Hour	5.49
30 Hour	5.79
36 Hour	*6.09*
42 Hour	*6.21*
48 Hour	*6.38*
60 Hour	*6.38*
72 Hour	6.38

Total Sample Size	Power: Clinically Acceptable Duration	Power: Positive Duration-Response
1500	0.717	0.935
1600	0.709	0.939
1700	0.726	0.951
**1800**	**0.718**	**0.959**
1900	0.728	0.966
2000	0.721	0.975
2100	0.708	0.977
2200	0.721	0.983
2300	0.712	0.978

Power is high for both components of the primary analysis across the range of sample sizes in each scenario above. As the total number of patients increase, power tends to increase, but the gains in power are small. For example, in scenario 3, power is very similar for both components of the primary analysis across the range of sample sizes. In scenario 2, all sample sizes provided at least 80% power for the component of determining a positive duration response. The sample size of 1800 patients was selected to balance the trade-off

between additional patients and the correspondingly small increase in power in these scenarios.

Additional simulation results showed that this choice of sample size would perform well in scenarios where the treatment effect is smaller. Scenarios 4 and 5 below show the power for both components of the primary endpoint analysis for two such scenarios.

Scenario 4 (Both Rhythm Types)

Arm	Mean Weighted mRS
6 Hour	4.85
12 Hour	5.12
18 Hour	5.39
24 Hour	*5.66*
30 Hour	*5.93*
36 Hour	*5.93*
42 Hour	*5.93*
48 Hour	5.93
60 Hour	5.93
72 Hour	5.93

Total Sample Size	Power	
	**Clinically Acceptable Duration**	**Positive Duration-Response**
1500	0.637	0.657
1600	0.648	0.687
1700	0.653	0.723
**1800**	**0.662**	**0.746**
1900	0.660	0.749
2000	0.680	0.800
2100	0.682	0.797
2200	0.687	0.822
2300	0.690	0.816

Scenario 5 (Both Rhythm Types)

Arm	Mean Weighted mRS
6 Hour	4.85
12 Hour	5.39
18 Hour	*5.93*
24 Hour	*5.93*
30 Hour	*5.93*
36 Hour	5.93
42 Hour	5.93
48 Hour	5.93
60 Hour	5.93
72 Hour	5.93

Total Sample Size	Power	
	**Clinically Acceptable Duration**	**Positive Duration-Response**
1500	0.607	0.464
1600	0.622	0.476
1700	0.654	0.522
**1800**	**0.640**	**0.552**
1900	0.670	0.566
2000	0.674	0.579
2100	0.682	0.599
2200	0.687	0.619
2300	0.711	0.652

Power is lower for both components of the primary analysis in scenarios 4 and 5 than scenarios 1, 2, and 3 described above because the maximum treatment effect is smaller. In Scenario 5, 1800 patients provides 64% power for selecting a clinically acceptable duration and 55% power for showing a positive duration-response curve. However, this is a challenging scenario for power regardless of the maximum sample size.

This figure illustrates data from the prior tables. It shows the effect of varying sample size from 1,500 to 2,300 on the probability of identifying a positive duration response and the probability of detecting the best duration in simulations in 3 reference scenarios with strong treatment effects, and two illustrative scenarios with weak treatment effects.

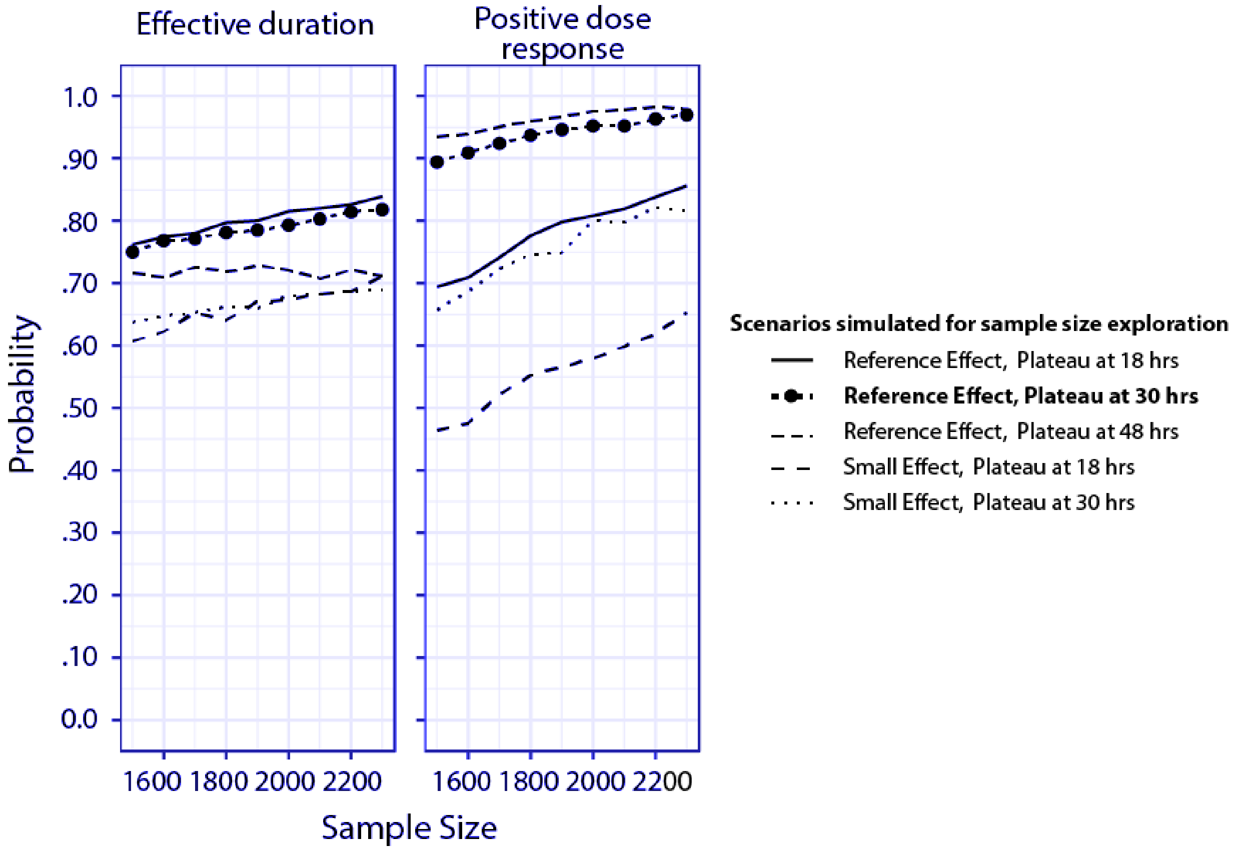

The 3 reference scenarios plateaus at 18, 30, or 48 hours. In the middle reference scenario, a sample size of 1800 provides a probability of 0.94 for identifying a positive duration response and 0.78 for correctly identifying the shortest effective duration. In general, it is harder to identify the positive duration response in early plateau scenarios because less of the curve is positive. In the early (18 hour) plateau reference scenario, a sample size of 1800 is the minimum required to provide a probability of 0.78 for identifying a positive duration response and 0.80 for correctly identifying the shortest effective duration In general it is harder to detect the best duration, at which the treatment effect has plateaued, in late plateau scenarios, because there are fewer data at the plateau. The trial is not designed to detect the small treatment effects, but still frequently identifies these.

4.8 Simulating Virtual Subjects

In order to simulate the design we need to create a methodology for simulating individual subjects, including their longitudinal results. These assumptions do not affect the analysis described above and are only made to simulate virtual subjects.

The approach to simulate a subject is to simulate their 90-day mRS outcome, then conditional on this value simulate their 30-day mRS outcome. This reverse order approach allows the probabilities of the 90-day mRS outcomes to be set explicitly, and the truth of the durations to be controlled easier.

In order to simulate subjects a probability vector for each of the 90-day outcomes is assumed. Let the probability of the 7 outcomes be $p_0, \ldots, p_6$. The true assumed mean weight for the treatment arm is

$$\sum_{m=0}^{6} p_m W(m).$$

The design uses the 30-day mRS outcome in the longitudinal model. The following probabilities are used to simulate the 30-day mRS outcome (columns) conditional on the 90-day mRS outcome (row).

$$\begin{array}{c} \\ 0 \\ 1 \\ 2 \\ 3 \\ 4 \\ 5 \\ 6 \end{array} \begin{array}{c} \begin{array}{ccccccc} 0 & 1 & 2 & 3 & 4 & 5 & 6 \end{array} \\ \begin{bmatrix} .3 & .3 & .2 & .15 & .04 & .01 & 0 \\ .1 & .5 & .2 & .15 & .04 & .01 & 0 \\ .05 & .05 & .5 & .25 & .14 & .01 & 0 \\ .01 & .05 & .05 & .5 & .35 & .04 & 0 \\ .01 & .01 & .01 & .07 & .7 & .2 & 0 \\ .01 & .01 & .01 & .01 & .26 & .7 & 0 \\ .01 & .01 & .01 & .01 & .06 & .4 & .5 \end{bmatrix} \end{array}.$$

Therefore, by specifying the 7 mRS outcomes creates a true mean response for each duration. For example, for the null hypothesis, the probabilities for the 7 outcomes are assumed to be:

mRS	0	1	2	3	4	5	6
Probability	0.20	0.15	0.5	0.05	0.05	0.10	0.30

The mean weighted truth for this scenario is 4.85. The truth for each of the 10 durations can be varied to create many different relative effects as well as different true duration

response relationships. For each true mean weighted mRS used in the scenarios above, we show the assumed probabilities across the 7 outcomes.

**mRS Assumptions**					
**Mean Weighted mRS**	**mRS 0**	**mRS 1**	**mRS 2**	**mRS 3**	**mRS 4-6**
4.25	0.18	0.13	0.13	0.04	0.47
4.85	0.2	0.15	0.15	0.05	0.45
5.015	0.205	0.155	0.155	0.055	0.43
5.18	0.21	0.16	0.16	0.06	0.41
5.2	0.22	0.16	0.15	0.06	0.41
5.27	0.21	0.17	0.16	0.06	0.4
5.34	0.215	0.17	0.17	0.05	0.395
5.345	0.215	0.165	0.165	0.065	0.39
5.39	0.22	0.17	0.17	0.05	0.39
5.51	0.22	0.17	0.17	0.07	0.37
5.615	0.225	0.175	0.175	0.065	0.36
5.62	0.22	0.18	0.18	0.06	0.36
5.675	0.225	0.175	0.175	0.075	0.35
5.77	0.25	0.17	0.15	0.09	0.34
5.84	0.23	0.18	0.18	0.08	0.33
5.93	0.24	0.19	0.19	0.05	0.33
5.95	0.23	0.19	0.19	0.07	0.32
6.08	0.26	0.18	0.15	0.11	0.3
6.14	0.24	0.19	0.19	0.085	0.295
6.24	0.27	0.18	0.15	0.12	0.28
6.38	0.25	0.2	0.2	0.08	0.27
6.44	0.25	0.2	0.2	0.09	0.26
6.59	0.29	0.19	0.15	0.13	0.24
6.9	0.3	0.2	0.15	0.15	0.2
7.04	0.27	0.22	0.22	0.1	0.19

Visualizations of distributions of mortality and mRS in virtual subjects

The following graphics illustrate the distributions of mRS scores (including the mortality) assumed for the virtual subjects assigned to each duration of cooling, and the relationship of those mRS distributions to the mean weighted mRS at each duration. Such distributions are used to generate virtual subjects for all simulated scenarios. Here, we present the three reference scenarios included in section 4.7 as examples.

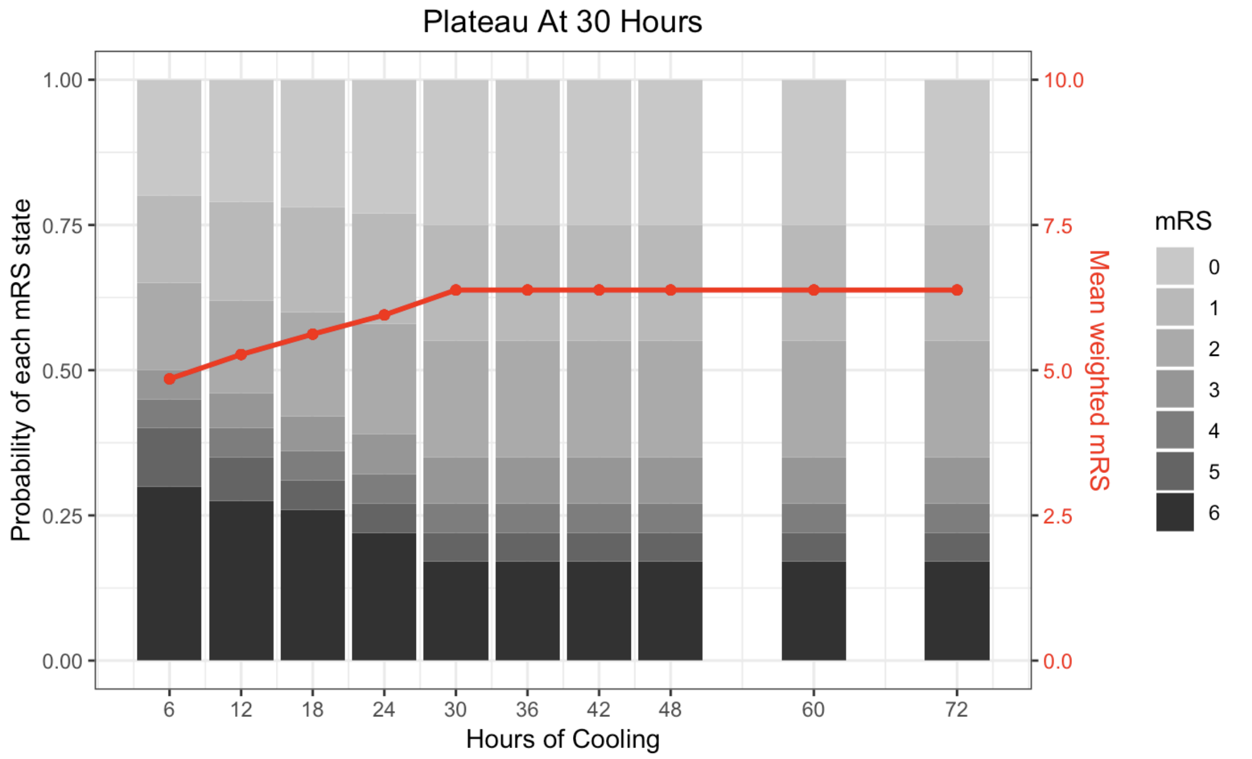

Mortality and neurological outcome (mRS) by duration of cooling group assignment
Strong Treatment Effect at 30 Hours (Scenario 32.32 described more fully in Section 4.2.2)

The 6 hour duration in all scenarios reflects the mRS distribution found in the null scenario. At the 6 hour cooling duration the assumed mRS 6 (mortality) and mRS 4-6 (bad outcome weighted the same as death) are 30% and 45% respectively, which are consistent with control groups in prior trials. The plateau durations represent mRS distributions for the effect size being evaluated in each scenario. This is a strong effect size scenario, which the trial is designed to detect, in which mRS 6 and mRS 4-6 decrease by 14% and 18%.

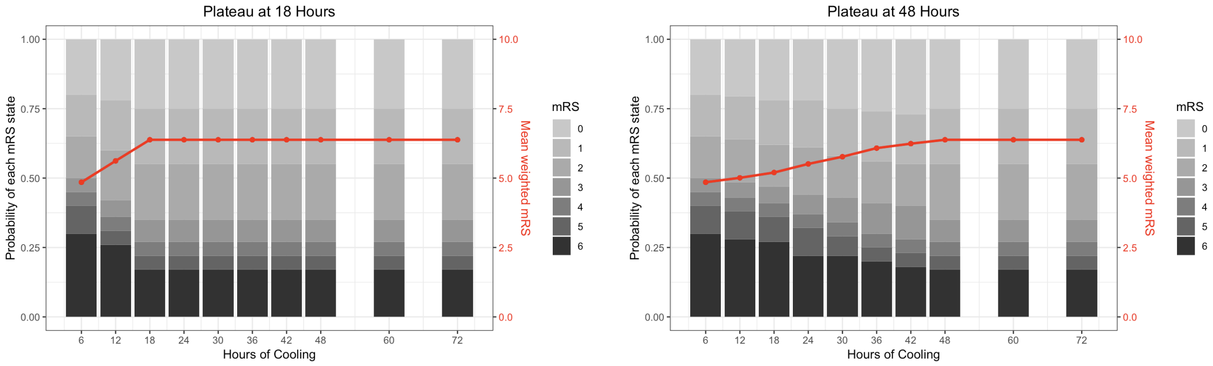

Mortality and neurological outcome (mRS) by duration of cooling group assignment Strong Effect at 18 (Scenario 31.31, section 4.2.7) or 48 (Scenario 53.53, section 4.4.5)

The mortality and mRS distributions in these two scenarios are the same in virtual subjects in the 6 hour duration and at durations with plateaued strong treatment effect, but the plateau occurs earlier or later than in the reference 30 hour plateau in the prior example.
